# Metformin directly targets the H3K27me3 demethylase KDM6A/UTX

**DOI:** 10.1111/acel.12772

**Published:** 2018-05-08

**Authors:** Elisabet Cuyàs, Sara Verdura, Laura Llorach‐Pares, Salvador Fernández‐Arroyo, Fedra Luciano‐Mateo, Noemí Cabré, Jan Stursa, Lukas Werner, Begoña Martin‐Castillo, Benoit Viollet, Jiri Neuzil, Jorge Joven, Alfons Nonell‐Canals, Melchor Sanchez‐Martinez, Javier A. Menendez

**Affiliations:** ^1^ ProCURE (Program Against Cancer Therapeutic Resistance) Metabolism & Cancer Group Catalan Institute of Oncology Girona Catalonia Spain; ^2^ Girona Biomedical Research Institute (IDIBGI) Girona Spain; ^3^ Mind the Byte Barcelona Spain; ^4^ Unitat de Recerca Biomèdica Hospital Universitari de Sant Joan IISPV Rovira i Virgili University Reus Spain; ^5^ Institute of Chemical Technology Prague Czech Republic; ^6^ Institute of Biotechnology Czech Academy of Sciences Prague‐West Czech Republic; ^7^ Unit of Clinical Research Catalan Institute of Oncology Girona Spain; ^8^ INSERM U1016 Institut Cochin Paris France; ^9^ CNRS UMR 8104 Paris France; ^10^ Université Paris Descartes Sorbonne Paris Cité Paris France; ^11^ School of Medical Science Menzies Health Institute Queensland Griffith University Southport Queensland Australia

**Keywords:** aging, cancer, chemoinformatics, computational screening, metformin

## Abstract

Metformin, the first drug chosen to be tested in a clinical trial aimed to target the biology of aging per se, has been clinically exploited for decades in the absence of a complete understanding of its therapeutic targets or chemical determinants. We here outline a systematic chemoinformatics approach to computationally predict biomolecular targets of metformin. Using several structure‐ and ligand‐based software tools and reference databases containing 1,300,000 chemical compounds and more than 9,000 binding sites protein cavities, we identified 41 putative metformin targets including several epigenetic modifiers such as the member of the H3K27me3‐specific demethylase subfamily, KDM6A/UTX. AlphaScreen and AlphaLISA assays confirmed the ability of metformin to inhibit the demethylation activity of purified KDM6A/UTX enzyme. Structural studies revealed that metformin might occupy the same set of residues involved in H3K27me3 binding and demethylation within the catalytic pocket of KDM6A/UTX. Millimolar metformin augmented global levels of H3K27me3 in cultured cells, including reversion of global loss of H3K27me3 occurring in premature aging syndromes, irrespective of mitochondrial complex I or AMPK. Pharmacological doses of metformin in drinking water or intraperitoneal injection significantly elevated the global levels of H3K27me3 in the hepatic tissue of low‐density lipoprotein receptor‐deficient mice and in the tumor tissues of highly aggressive breast cancer xenograft‐bearing mice. Moreover, nondiabetic breast cancer patients receiving oral metformin in addition to standard therapy presented an elevated level of circulating H3K27me3. Our biocomputational approach coupled to experimental validation reveals that metformin might directly regulate the biological machinery of aging by targeting core chromatin modifiers of the epigenome.

## 1 INTRODUCTION

The TAME (Targeting Aging with Metformin) clinical trial has been designed to evaluate the capacity of the antidiabetic biguanide metformin to delay the manifestation of age‐associated disorders (Barzilai, Crandall, Kritchevsky & Espeland, 2016). By enrolling patients aged 65–79 years diagnosed with one single age‐associated condition and then assessing the global impact of metformin on a composite outcome including cardiovascular events, cancer, dementia, mortality, and other functional and geriatric endpoints, this paradigm‐shifting study aimed to target the aging process per se (Newman et al., 2016; Figure S1A). If the positive consequences of metformin extend beyond an isolated impact on each separate age‐related disease, the TAME study might pave the way for the development of new healthspan‐promoting treatments aimed to promote reduction in age‐associated multimorbidity.

Metformin might exert multiple healthspan‐promoting effects by correcting deregulated nutrient/energy‐sensing metabolic axes such as insulin/IGF‐1 and AMPK/mTOR, one of the metabolic hallmarks of aging (López‐Otín, Blasco, Partridge, Serrano & Kroemer, 2013; López‐Otín, Galluzzi, Freije, Madeo & Kroemer, 2016). The ability of metformin to promote such metabolic fitness and operate as an anti‐aging tool is commonly perceived as the sum of the pleiotropic effects due to its primary action on a single master mechanism. Since the first description of inhibition of mitochondrial energy transfer by guanidines in 1963, mitochondrial complex I (mCI) of the electron transport chain has been commonly viewed as the primary target of metformin (Bridges, Jones, Pollak & Hirst, 2014; Chance & Hollunger, 1963). Although approved as an insulin‐lowering agent for type 2 diabetes or other hyperinsulinemic conditions, the ongoing use of metformin has led to the discovery of unanticipated and multifaceted actions in several chronic conditions. Indeed, metformin is an archetypical example of an approved drug that has been clinically exploited without a complete understanding of its precise therapeutic targets or chemical determinants (Sweeney, Raymer & Lockwood, 2003). Here, using a systematic chemoinformatics approach (Figure S1B) coupled to laboratory‐based confirmatory testing, we sought to computationally predict and experimentally validate new biomolecular targets through which metformin might operate as a polytherapeutic tool capable of targeting the biological machinery of aging.

## 2 RESULTS

### 2.1 Structure‐based virtual profiling of metformin targets

Thirteen structure‐based virtual profiling (VP) predicted targets of metformin (Table 1) were selected based on interaction energies (≤6.1 kcal/mol). Among others, the targets included the metalloenzymes glutamate carboxypeptidase 2 and N‐acetylated‐alpha‐linked acidic dipeptidase 2; purine nucleoside phosphorylase, an essential enzyme of the purine salvage pathway; CAD protein, a rate‐limiting trifunctional protein involved in de novo synthesis of pyrimidine nucleotides; arginases 1 and 2, two enzymes involved in L‐arginine/nitric oxide metabolism that promote vascular endothelial inflammation and senescence, atherogenesis, and cardiovascular aging; dynamin‐1, a member of the mitochondrial division machinery associated with neurodegenerative disorders; and kallikrein‐7, a serine protease involved in skin homeostasis and inflammation.

**Table 1:** Selected metformin targets from structure‐ and ligand‐based VS experiments

Uniprot ID	Protein name	PDBID	Binding energy^a^
Structure‐based			
Q9Y3Q0	N‐acetylated‐alpha‐linked acidic dipeptidase 2 (NAALAD2, GCPIII)	3FED	−6.5
Q16769	Glutaminyl‐peptide cyclotransferase (QPCT)	3PBB	−6.4
P00491	Purine nucleoside phosphorylase (PNP)	3PHB	−6.4
P05089	Arginase‐1 (ARG1)	3GN0	−6.4
P27708	CAD protein (CAD)	4C6E	−6.3
P42262	Glutamate receptor 2 (GRM2)	2XHD	−6.3
Q04609	Glutamate carboxypeptidase 2 (GCPII)	4NGS	−6.2
Q05193	Dynamin‐1 (DNM1)	2X2E	−6.2
P78540	Arginase‐2, mitochondrial (ARG2)	4IXU	−6.2
P14618	Pyruvate kinase (PKM)	1T5A	−6.2
P28845	Corticosteroid 11‐beta‐dehydrogenase isozyme 1 (HSD11B1)	3PDJ	−6.1
P34998	Corticotropin‐releasing factor receptor 1 (CRHR1)	3EHU	−6.1
P49862	Kallikrein‐7 (KLK7)	2QXG	−6.1
Ligand‐based			
P27487	Adenosine deaminase complexing protein 2 (ADCP‐2)		
O15244	Solute carrier family 22 member 2 (SLC22A2, OCT2)		
Q92830	Histone acetyltransferase GCN5 (KAT2A)		
O75496	Geminin (GMNN)		
O15245	Solute carrier family 22 member 1 (SLC22A1, OCT1)		
Q9Y6L6	Solute carrier organic anion transporter family member 1B1 (SLCO1B1)		
P02545	Prelamin‐A/C (LMNA)		
O94956	Solute carrier organic anion transporter family member 2B1 (SLCO2B1)		
Q96FL8	Multidrug and toxin extrusion protein 1 (MATE1, SLC47A1)		
Q9NPD5	Solute carrier organic anion transporter family member 1B3 (SLCO1B3)		
O75164	Lysine‐specific demethylase 4A (KDM4A)		
O95050	Indolethylamine N‐methyltransferase (INMT)		
Q9UNA4	DNA polymerase iota (POLI)		
P51449	Nuclear receptor ROR‐gamma (RORC)		
P00915	Carbonic anhydrase I (CA1)		
P04150	Glucocorticoid receptor (GCR, NR3C1)		
P03956	Matrix metalloproteinase‐1 (MMP1)		
P08254	Matrix metalloproteinase‐3 (MMP3)		
P84022	Mothers against decapentaplegic homolog 3 (SMAD3)		
Q99720	Sigma nonopioid intracellular receptor 1 (OPRS1, SIGMAR1)		
Q7Z2H8	Proton‐coupled amino acid transporter 1 (SLC36A1)		
P63092	Guanine nucleotide‐binding protein G(s), subunit alpha (GNAS)		
P83916	Chromobox protein homolog 1 (CBX1)		
Q9H3R0	Lysine‐specific demethylase 4C (KDM4C)		
P39748	Flap endonuclease 1 (FEN1)		
P10145	Interleukin‐8 (IL‐8, CXCL8)		
P08253	Matrix metalloproteinase‐2 (MMP2)		
Q9NUW8	Tyrosyl‐DNA phosphodiesterase 1 (TDP1)		

a The binding energy is obtained during the virtual profiling experiment as it is docking‐based. The more negative the binding energy, the more plausible the interaction.

### 2.2 Ligand‐based virtual profiling of metformin

Twenty‐eight ligand‐based VP targets were selected based on the similarity of structural and physicochemical properties to metformin (Table 1). Among others, the targets included several membrane transporters known to be involved in metformin pharmacokinetics including solute carrier family 22 members SLC22A1/OCT1 and SLC22A/OCT2, the main transporters for metformin uptake and clearance, respectively, and multidrug and toxin extrusion protein MATE1/SLC47A1, which mediates metformin secretion (Pernicova & Korbonits, 2014). Other transporters included several members of the uptake organic anion transporting polypeptides (OATP) family (SLC21/SLCO) such as SLCO1B1/OATP1B1, SLCO2B1/OATP2B1, and SLCO1B3/OATP1B3, which are known to transport statins, antibiotics, chemotherapeutics, antihistaminic drugs, and diuretics (König, Müller & Fromm, 2013). In addition, the intracellular proton‐assisted amino acid transporter 1 PAT1/SLC36A1, which is an essential component of the amino acid‐sensing engine that drives mTORC1 activation from late endosomes and lysosomes, was included (Heublein et al., 2010). Several zinc‐containing matrix‐degrading endopeptidases (MMP‐1, MMP‐2, MMP‐3), which are known regulators of tissue remodeling in aging and cancer, were also included in the selected list of metformin targets.

A notable number of DNA‐interacting enzymes are listed in the ligand‐based VP for metformin (Table 1), including geminin, an inhibitor of replication initiation that prevents DNA rereplication‐driven genomic instability and cellular senescence, DNA polymerase iota, and flap endonuclease 1, a key interaction partner of the RecQ helicases involved in genome maintenance and DNA metabolism during accelerated aging genetic disorders and cancer. The ligand‐based target list for metformin also contained several chromatin‐modifying epigenetic regulators including lysine‐specific demethylases 4A (KDM4A) and 4C (KDM4C), Jumonji 2 proteins that demethylate di‐ and trimethylated histone 3 lysine 9 (H3K9me2/3) and H3K36me3, two epigenetic markers closely related to the aging process (Sen et al., 2015; Zhang et al., 2015). Moreover, when the default similarity score of 0.8 used for ligand‐based VP was reduced to 0.65–0.7, a number of histone lysine demethylases including KDM2A, KDM5A/C, KDM4B/D/E, KDM7, KDM5C, KDM6A/UTX, KDM6B/JMJD3, and LSD1 (KDM1A) emerged as new putative targets of metformin.

### 2.3 Docking and molecular dynamics validation of metformin targets

To validate the VP results, we performed in silico binding experiments using rigid docking + short molecular dynamics (MD) calculations, the latter aiming to simulate flexible docking conditions. In total, we performed docking calculations for 33 metformin targets. Docking calculations of metformin against the selected targets, which were run twice to avoid false positives, revealed low binding energies, ranging from −3.2 kcal/mol to −6.5 kcal/mol (Table S1). To add protein flexibility to the analysis and to test the stability of the selected metformin‐target complexes, allowing us to filter out poorly interacting compounds, we carried out short MD simulations of 1 ns. We then performed Molecular mechanics‐generalized born surface area (MM/GBSA) calculations to estimate the free energy of the binding of small ligands such as metformin to biological macromolecules (Genheden & Ryde, 2015). MM/GBSA‐based estimation of ligand‐binding affinities considers the dynamic nature of the protein and is therefore more reliable to provide a realistic view of metformin‐binding affinity than rigid docking estimations. The ranked energies obtained following MM/GBSA rescoring calculations over MD simulations are summarized in Tables S2 and S3.

### 2.4 New possible indications and biomolecular targets for metformin

New possible indications of metformin were evaluated by crossing the ligand‐ and structure‐based metformin targets obtained after VP with DisGeNET, a database containing 429,036 associations between 17,381 genes and 15,093 diseases, disorders, and clinical or abnormal human phenotypes (Piñero et al., 2017). A graphical summary of the diseases in which the predicted metformin targets are involved suggested an enrichment of the most prevalent aging‐related diseases, including neoplasms/carcinoma, cardiovascular, and neurodegenerative disorders (Figure S1B). Using the WEB‐based Gene SeT AnaLysis Toolkit to statistically evaluate the DisGeNet findings, we confirmed the nonrandom and unbiased distribution of such metformin target‐associated morbidities (Table S4). Gene Ontology (GO) was extracted from UniProtKB database to provide a detailed description of the molecular functions and biological processes of the ligand‐ and structure‐based metformin targets (Table S5).

We then performed a Protein ANalysis THrough Evolutionary Relationship (PANTHER) overrepresentation test using as a reference list the comprehensive GO annotations directly imported from the GO database that includes 20,972 mapped identifiers. Fourteen different classes of molecular functions were found significantly enriched in the list of metformin targets (Table S6). Beyond the expected overrepresentation of molecular functions related to transmembrane transporter activity, most of the molecular functions were related to metal ion binding and peptidase activity.

Our computational de‐orphanization of metformin further revealed that DNA/histone‐interacting proteins might be viewed as unforeseen targets for anti‐aging metformin. The best‐aligned 3D and 2D metformin poses (after MD simulations) and the key interacting residues for metformin binding to such DNA/histone‐related targets are illustrated in Figure S2 and Table S7. When considering the activity and phenotype of similar molecules against the DNA/histone‐interacting targets of metformin, one could infer that, with the exception of the predicted activation of geminin, metformin mostly behaves as an inhibitor of chromatin structure modifiers (Tables S8 and S9; Figure S3A,B).

### 2.5 Metformin specifically augments global levels of H3K27me3 irrespective of mitochondrial complex I and AMPK

**Figure 1:**
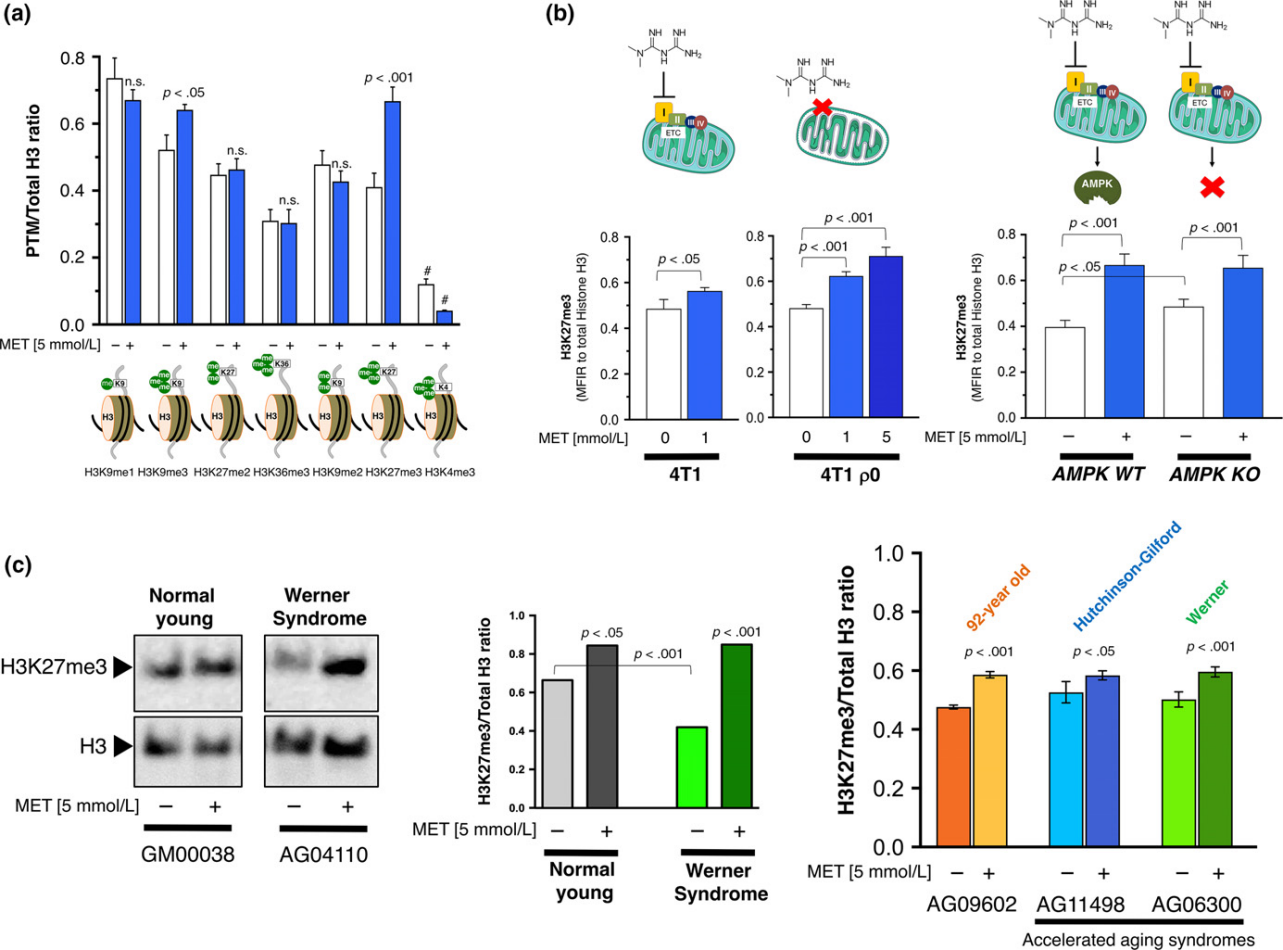
Metformin effects on histone H3 post‐translational modifications. (a) Mouse embryonic fibroblasts (MEFs) were treated with 5 mmol/L metformin for 48 hr. Acid extracts were prepared, and lysates were used to evaluate H3K9me1, H3K9me3, H3K27me2, H3K36me3, H3K9me2, H3K27me3, and H3K4m3 Ab‐conjugated beads in multiplex along with H3 Total beads for normalization to determine relative post‐translational modifications (PTM) values using the active motif histone H3 PTM multiplex assay. (b) H3K27me3 global levels in metformin‐treated 4T1/4T1 ρ0 and AMPK WT/AMPK KO isogenic pairs of cells were determined as in (a). (c) Left. Representative immunoblots for H3K27me3 histone modification in normal young fibroblasts (GM00038, 9 years old) and Werner's syndrome (WS) fibroblasts (AG04110, 13 years old). Also shown are total H3 controls. n.s. nonsignificant differences relative to untreated control cells by Student's *t* test for paired values; *p* < .05, *p* < .001 relative to control cells by Student's *t* test for paired values; # mean fluorescence intensities were below the range allowing the accurate calculation of PTM values. Right. H3K27me3 global levels in metformin‐treated old fibroblasts (AG09602, 92 years old), HPGS fibroblasts (AG11498, 14 years old), and WS fibroblasts (AG06300, 37 years old) were determined as in (a)

**Figure 2:**
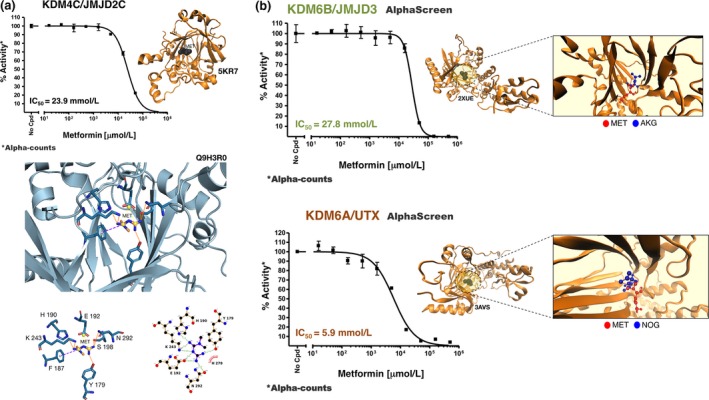
Metformin effects on the demethylation activities of KDM4C/JMJD2C, KDM6B/JMJD3, and KDM6A/UTX. (a) A dose–response curve of KDM4C activity was created with Graphpad Prism by plotting AlphaScreen signals as the function of metformin concentration. Overall structure and views of the interaction between metformin and KDM4C (PDB code 5KR7). The coordinating residues are numbered. Metformin (MET) is placed over the bottom part of the cavity defined by 6X9 (6‐ethyl‐2,5‐dimethyl‐7‐oxidanylidene‐4~{H}‐pyrazolo[1,5‐α]pyrimidine‐3‐carbonitrile), the crystallographic ligand of PDB ID 5KR7. 6X9 coordinates Fe^2+^, suggesting that metformin might share a metal‐interacting role. Metformin is hydrogen bonded to K243, H190, E192, S198, N292, and Y179. Metformin additionally shows a π‐cation interaction with F187 as well as a hydrophobic interaction with H278. Of note, metformin shares all the residues involved in the binding mode of 6X9 at the bottom part of the cavity. (b) Dose–response curves of KDM6B/JMJD3 (top) and KDM6A/UTX (bottom) demethylation activity were created with Graphpad Prism by plotting AlphaScreen signals as the function of metformin concentration. Overall structures and views of the interaction between metformin (red) and the crystallographic ligands AKG and NOG (blue) in the catalytic pockets of KDM6B/JMJD3 (PDB code 2XUE) and KDM6B/UTX (PDSB code 3AVS), respectively

To test the hypothesis that metformin might target the chromatin‐modifying activities of specific KDMs that actively remove well‐established aging‐related epigenetic changes (e.g., di‐ and trimethylation marks on histone H3 lysines 4, 9, 27, and 36; Pal & Tyler, 2016; Han & Brunet, 2012; Benayoun, Pollina & Brunet, 2015; Booth & Brunet, 2016), we first examined the global levels of several methylation marks, viz. H3K9me1, H3K9me3, H3K27me2, H3K36me3, H3K9me2, H3K27me3, and H3K4me3, in SV40‐immortalized mouse embryonic fibroblasts (MEFs). When the relative amount of each histone modification was interrogated by normalizing it to total histone H3 levels using a Luminex histone H3 post‐translational modification assay, we observed a striking and almost exclusive upregulation of H3K27me3 when MEFs were cultured in the presence of millimolar metformin (Figure 1a). While a slight increase in the global level of H3K9me3 was also noted, the remainder of histone H3 methylation marks were mostly unaltered in response to metformin (Figure 1a). The in silico prediction that metformin may target H3K9me3 demethylases such as KDM4C/JMJD2C was confirmed by AlphaScreen assays, which demonstrated the in vitro capacity of metformin to significantly decrease KDM4C/JMJD2C activity in a dose‐dependent manner (IC_50_ = 23.9 mmol/L; Figure 2a).

To evaluate whether the ability of metformin to modify H3K27m3 was hindered in the absence of its primary mitochondrial target, we employed an experimental pair of mCI‐containing 4T1 mammary carcinoma cells and a 4T1 subline depleted of mitochondrial DNA (ρ^0^ cells; Tan et al., 2015). mCI‐deficient cells were exquisitely responsive to the upregulatory actions of 1 mmol/L metformin on H3K27me3 levels and. At metformin concentrations (5 mmol/L) that were cytotoxic and precluded histone H3 PTM analyses in 4T1 parental cells, we noted a dramatic dose‐dependent augmentation of the global levels of H3K27me3 in 4T1 ρ^0^ cells (Figure 1b). Because the above experiments might fail to uncouple mCI‐dependent from AMPK‐related effects of metformin on H3K27me3, we re‐assessed the capacity of metformin to modify the global levels of H3K27me3 in isogenic MEFs deficient or not for *AMPK* α*1* and α*2* catalytic subunits *(*α*1* ^*−/−*^α*2* ^*−/−*^, AMPK double knockout [DKO]), thus containing the primary target of metformin (mCI) but lacking functional AMPK signaling (Griss et al., 2015). We observed that, while a slight but significant elevation in H3K27me3 occurred in AMPKα DKO fibroblasts (Wan et al., 2018), they still remained responsive to the upregulatory actions of metformin on H3K27me3 (Figure 1b).

We then assessed the impact of metformin treatment on the global status of H3K27me3 in dermal fibroblasts obtained from normal old donors and from patients with Werner's syndrome (WS). Immunoblotting analysis revealed the ability of metformin to promote the restoration of the extremely low level of H3K27me3 in prematurely aging WS fibroblasts to the normal level found in normal young donors (Figure 1c, left). Luminex‐based quantification of the H3K27me3 mark confirmed the ability of metformin to significantly augment the global levels of H3K27me3 in human fibroblasts from advanced age (but normal) donors as well as from individual with accelerated aging syndromes including Hutchinson–Gilford progeria syndrome (HGPS) (Figure 1c, right).

### 2.6 Metformin directly targets the demethylation activity of KDM6A/UTX

**Figure 3:**
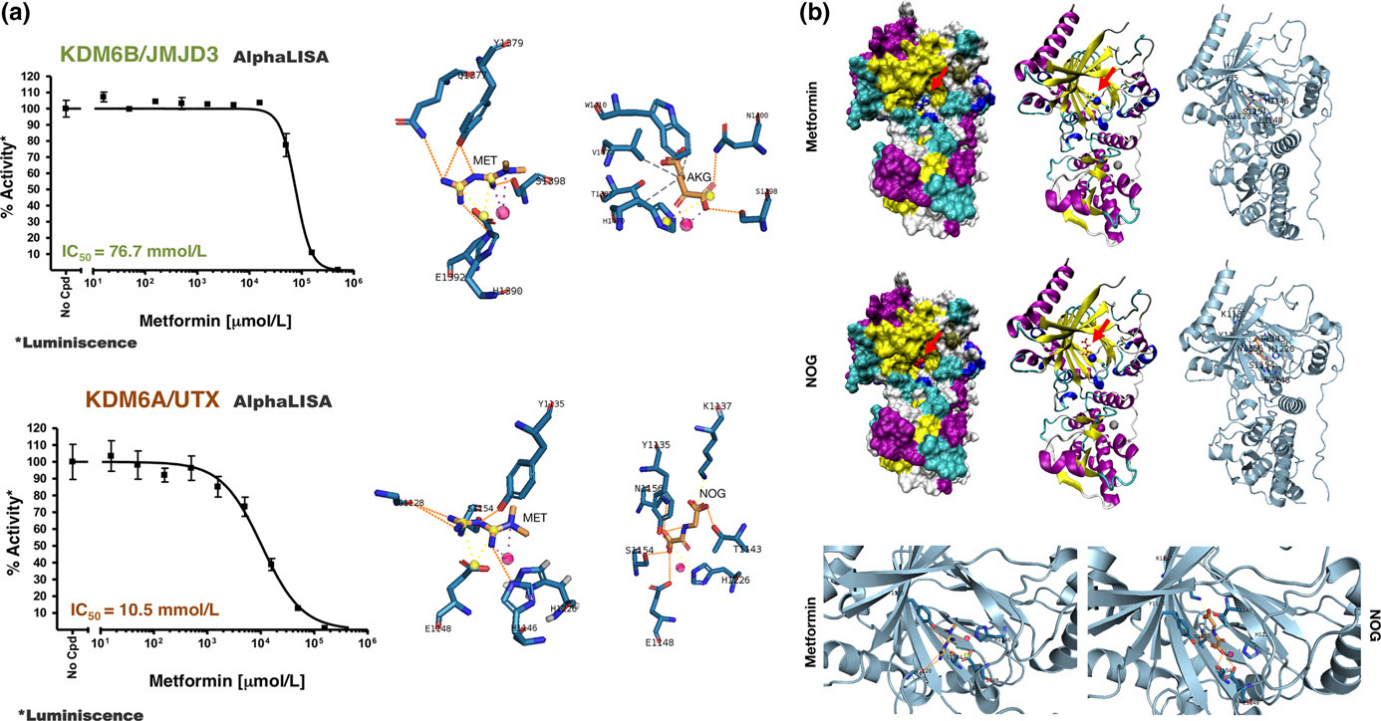
Metformin effects on the demethylation activities of KDM6B/JMJD3 and KDM6A/UTX. (a) Left panels. Dose–response curves of KDM6B/JMJD3 (top) and KDM6A/UTX (bottom) demethylation activity were created with Graphpad Prism by plotting AlphaLISA signals as the function of metformin concentration. Right panels. Detailed interactions between metformin and the crystallographic ligands AKG and NOG (red arrows) in the catalytic pockets of KDM6B/JMJD3 (top) and KDM6A/UTX (bottom), respectively. (b) Comparison of metformin and NOG coordination in the KDM6B/UTX structure. The coordinating residues are numbered

We utilized AlphaScreen assays to explore whether metformin directly inhibited the H3K27m3 demethylation activities of KDM6A/UTX and KDM6B/JMJD3. Whereas the IC_50_ of metformin for KDM6B/JMJD3 was >20 mmol/L (Figure 2b, top), it was noteworthy that metformin had an IC_50_ of 5.9 mmol/L for KDM6A/UTX (Figure 2b, bottom), a concentration similar to that employed to promote a significant augmentation of H3K27me3 global levels in cell‐based experiments. Moreover, while the demethylase activity of purified KDM6A/UTX was almost completely inhibited in the presence of 15 mmol/L metformin (>80% reduction; Figure 2b, bottom), an equivalent concentration reduced the demethylase activities of KMD4C/JMJD2C (Figure 2a) and KDM6B/JMJD3 (Figure 2b, top) by only 20%–30%. Such an ability of metformin to preferentially inhibit KDM6A/UTX over KDM6B/JMJD3 was further confirmed by AlphaLISA immunoassays, which demonstrated IC_50_ values of 10.5 and 76.7 mmol/L metformin for KDM6A/UTX and KDM6B/JMJD3, respectively (Figure 3a).

To gain insight into the inhibitory mechanism of metformin against KDM6A/UTX, we resolved the binding mode of metformin with the fragment corresponding to the C‐terminal region of KDM6A/UTX, which is known to exhibit demethylase activity against H3K27me3 (Sengoku & Yokoyama, 2011; Kruidenier et al., 2012). The metformin‐binding mode to KDM6A/UTX employed the crystal structure 3AVS in complex with N‐oxalylglycine (NOG, a nonreactive analog of AKG and Ni^2+^, which coordinates the Fe^2+^‐binding site but does not support catalysis). Despite their structural similarity, metformin accommodates in a slightly different manner to NOG by occupying the bottom‐left corner of the Ni^2+^‐coordinated binding site (Figure 3a,b). Thus, while residues K1137, T1143, and N1156 interacting with NOG in the upper part of the pocket are not shared with metformin, the interactions with Y1135, E1148, and S1154 are shared with NOG and are involved in the binding of metformin to the catalytic site of KDM6A/UTX. Although metformin does not mimic the binding mode of NOG, it maintains its metal coordination. Moreover, metformin hydrogen bonds with G1228, S1154, Y1135, and H1146, and charge–charge interacts with E1148, with all of these residues involved in H3K27me3 binding and catalysis (Figure 3a,b).

Metformin poses in a significantly different manner to the KDM6B/JMJD3 crystallographic ligand AKG (PDBID 2XUE; Figure 3a). Metformin hydrogen bonds with Y1379, Q1377, S1398, and H1390, and charge–charge interacts with E1392. Thus, only the S1398 residue is shared with the binding mode of AKG, which instead involves W1410, V1472, T1387, and H1470. Despite the different binding mode with respect to AKG, metformin occupies the bottom‐left corner of the Fe^2+^‐coordinated binding site at the H3 binding site of KDM6B/JMJD3 (Figure 3a).

### 2.7 Pharmacologically relevant biguanides inhibit the demethylation activity of KDM6A/UTX

**Figure 4:**
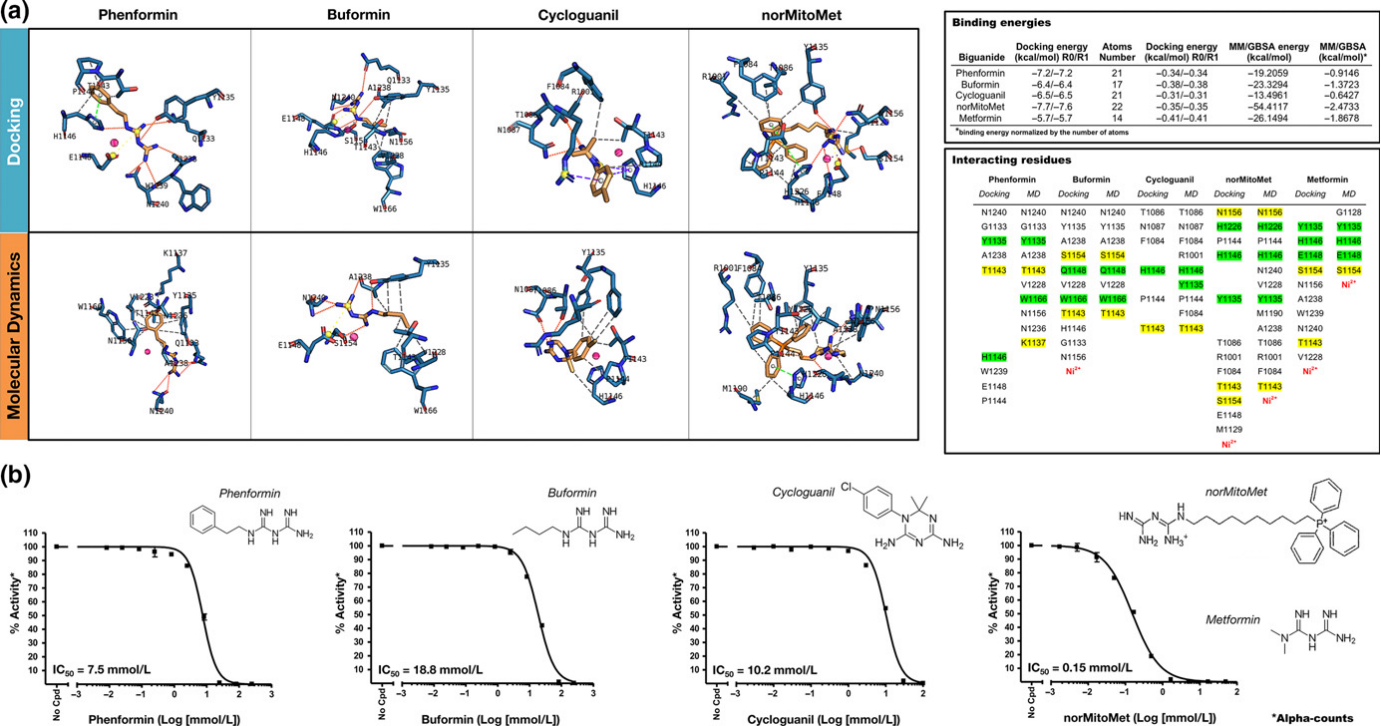
Effects of pharmacologically relevant biguanides on the demethylation activity of KDM6A/UTX. (a) Left panels. Detailed interactions between biguanides and the crystallographic ligand NOG (red arrows) in the catalytic pockets of KDM6A/UTX. Right panels. Docking binding energies and Molecular mechanics‐generalized born surface area (MM/GBSA)‐based energy rescoring calculation over molecular dynamics simulation of biguanides against KDM6A/UTX. The more negative the binding energy, the more plausible the interaction. Interacting residues that are meant to establish key interactions with the crystallographic ligand NOG (PDB 3AVS) are highlighted in a yellow box. Key catalytic residues contributing to the mechanism of action of KDM6A/UTX proposed in previous studies are highlighted in a green box. (b) Dose–response curves of KDM6A/UTX demethylation activity were created with Graphpad Prism by plotting AlphaLISA signals as the function of pharmacological relevant biguanides beyond metformin (dimethyl biguanide), including the antidiabetic and potentially antineoplastic biguanides phenformin (phenylethyl biguanide) and buformin (butyl biguanide), the antimalarial biguanide cycloguanil (the active metabolite of proguanil), and norMitoMet (a metformin‐TTP^+^ derivative)

The evaluation of the binding mode of several metformin‐related biguanides to the catalytic site of KDM6A/UTX revealed a differential but overlapping utilization of interacting residues shared with the crystallographic ligand NOG (K1137, T1143, S1154, and N1156) and other key interacting residues reported in the literature (H1146, E1148, W1166, and H1226) (Figure 4a). norMitoMet showed the largest number of contact residues (up to 13 in MD studies) and bound to the catalytic site of KDM6A/UTX with the highest energy value among all the biguanides tested (−54.4117 kcal/mol, Figure 4), which was notably higher than those binding energies found when using buformin, phenformin or cycloguanil (that ranged from −13.4961 to −23.3294 kcal/mol), all of them lower than those initially observed with metformin (−26.1494 kcal/mol, Table S3). AlphaScreen assays confirmed that norMitoMet worked as the most potent inhibitor of the demethylase activity of purified KDM6A/UTX among all the biguanides tested (IC_50_ = 0.15 mmol/L, ≅40‐fold lower than metformin IC_50_), whereas phenformin, cycloguanil, and buformin were slightly less potent than metformin at inhibiting the enzymatic activity of KDM6A/UTX (Figure 4b).

**Figure 5:**
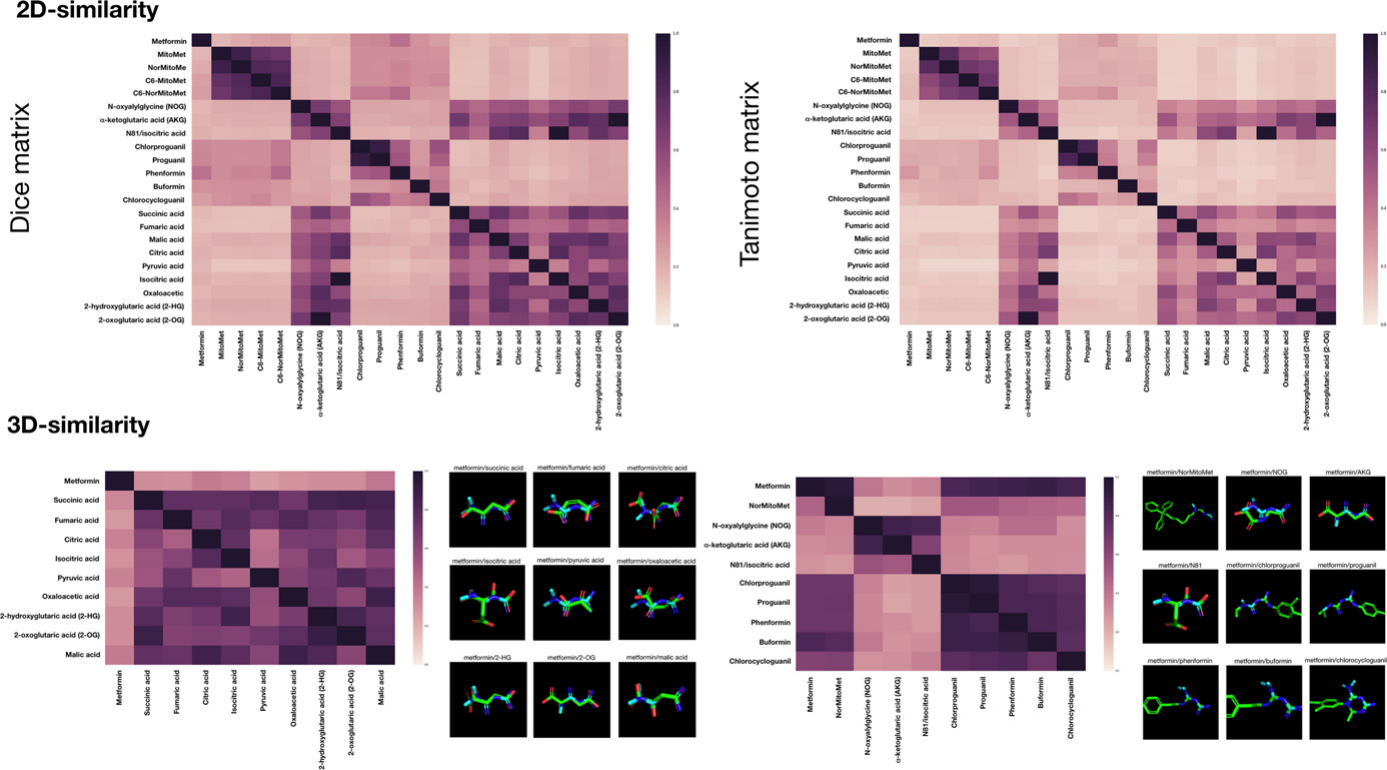
Evaluation of molecular similarity relationships between biguanides and KDM‐targeting metabolites. Left. 2D molecular similarity matrices for pairwise comparisons between biguanides, KDM crystallographic ligands, and TCA intermediates. Dice (left) and Tanimoto (right) similarity coefficients were calculated as the weighted average of the outcomes of the Atom pair, topological torsion, and Morgan/circular molecular fingerprints employed to computational encode the structure and properties in a given compound. Right. 3D molecular similarity matrices for pairwise comparisons between biguanides, KDM crystallographic ligands, and TCA intermediates. 3D similarity coefficients were calculated based on a combination of molecular shape complementarity and pharmacophoric features in the 3D space

On the basis of the similar property principle (Nigsch & Mitchell, 2008), which states that structural similar molecules are more likely to have similar properties and biological activities, we used 2D and 3D molecular fingerprints to disentangle whether biguanides functioned as KDM inhibitors by mimicking KDM crystallographic ligands and/or tricarboxylic acid cycle (TCA) intermediates that have been shown to interact with KDMs (Tarhonskaya et al., 2017) and extend lifespan (Mishur et al., 2016). When pairwise similarity calculations were measured using Dice and Tanimoto coefficients, we failed to observe a significant 2D structural resemblance between biguanides and KDM‐targeted metabolites (Figure 5, top panels). 3D similarity searches failed also to identify significant physicochemical similarities between biguanides and KDM metabolites (Figure 5, bottom panels). 2D and 3D searches concluded that biguanides grouped closer between them while KDM‐targeted metabolites structurally and physicochemical behaved as distant molecules compared to biguanides in the molecular space.

### 2.8 Pharmacological dosage of metformin augments global H3K27me3 in vivo

**Figure 6:**
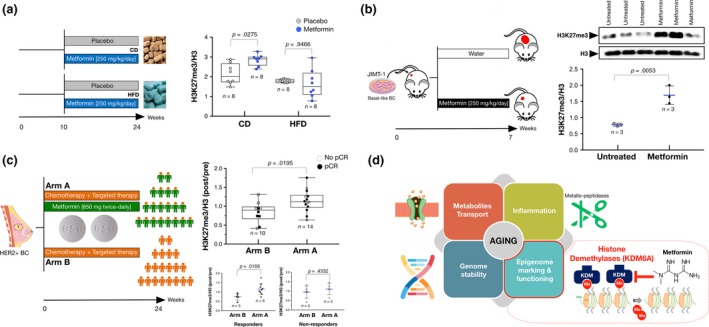
Effects of physiological dosage of metformin on the global expression of H3K27me3 in vivo. (a) Box plots indicating median, interquartile ranges, whiskers, and ranges for H3K27me3 in hepatic tissues obtained in *Lldr* ^*−/−*^ female mice upon feeding with experimental diets and use for metformin for 14 weeks; CD, chow diet; HFD, high‐fat diet. (b) Representative immunoblots for H3K27me3 histone modification in tumor tissues obtained from JIMT‐1 xenograft‐bearing mice treated with 250 mg/kg/day metformin during 7 weeks (Cufi et al., 2012). Also shown are total H3 controls. (c) Box plots indicating median, interquartile ranges, whiskers, and ranges for circulating H3K27me3 in nondiabetic breast cancer patients who were randomly assigned to receive daily metformin (850 mg twice‐daily) for 24 weeks concurrently with 12 cycles of weekly paclitaxel plus trastuzumab followed by four cycles of 3‐weekly fluorouracil, epirubicin, cyclophosphamide plus trastuzumab (arm A), or equivalent sequential chemotherapy plus trastuzumab without metformin, followed by surgery (arm B). Pathological complete response (pCR) was defined as absence of invasive tumor cells in breast and axilla following the completion of treatment at the time of surgery. (d) Biomolecular targets for metformin: A four‐faceted approach to aging. The ability of metformin to operate as a polytherapeutic tool functioning as a bona fide anti‐aging medicine might involve multifaceted mechanisms of action, including substrate competition phenomena with metabolites or nutrients, metal‐interactive regulation of protein functioning (e.g., inflammation‐related proteases), defense of the integrity/stability of the genome, and epigenome marking/functioning, including the H3K27me3 demethylase KDM6A/UTX

We evaluated the effects of 14‐week treatment of metformin dissolved in drinking water (5 mg/ml) on the global status of H3K27me3 in the hepatic tissue of low‐density lipoprotein receptor‐deficient (*Ldlr* ^*−/−*^) female mice. Mice treated with metformin showed a significant augmentation of hepatic H3K27me3 when fed with normal chow diet, but not in animals fed a high‐fat diet (Figure 6a). We evaluated also the effects of metformin treatment on the global status of H3K27me3 in highly aggressive human breast cancer xenografts (JIMT‐1 cell line) subcutaneously injected into female athymic nude mice (Cufi et al., 2012). Immunoblotting analyses of tumor tissues obtained from animals receiving 250 mg kg^−1^ day^−1^ metformin given intraperitoneally (i.p.) revealed a striking augmentation of the global levels of H3K27me3 when compared with vehicle‐administered mice (Figure 6b). We finally assessed the levels of circulating H3K27me3 in nondiabetic HER2‐positive breast cancer patients treated with preoperative metformin (Martin‐Castillo et al., 2010; Pernas et al., 2017). When we compared post‐treatment and pretreatment serum, circulating H3K27me3 was found to be significantly augmented in those patients that, in addition to the standard neoadjuvant regimen including anthracycline/taxane‐based chemotherapy and trastuzumab, simultaneously received 850 mg metformin twice‐daily for 24 weeks before surgery (Figure 6c). Such differential effects of metformin were found in the serum of patients whose tumors responded favorably to treatment (achieving a pathological complete response) when compared to those who did not respond (Figure 6c).

## 3 DISCUSSION

We provide biocomputational evidence to suggest that the capacity of metformin to operate as a polytherapeutic anti‐aging tool likely involves diverse mechanisms of action, such as substrate competition phenomena with metabolites or nutrients, metal‐interactive regulation of protein functioning (e.g., inflammation‐related proteases), genome stability, and epigenome marking and functioning (Figure 6d).

Metformin is a highly hydrophilic drug and is understood to require transporters to cross membranes (Pernicova & Korbonits, 2014). Consistent with this view, our ligand‐based VP, involving in silico screening, accurately predicted up to eight different membrane transporters interacting with metformin. These findings support the notion that since transporters involved in uptake and extrusion of metformin were designed by nature for endogenous substrates, the metabolic effects of metformin might involve substrate competition phenomena with metabolites or nutrients (Glossmann & Reider, 2013). Metformin and related biguanides are known to bind endogenous metals such as Zn^2+^, Cu^2+^, Fe^3+^ that independently inhibit pro‐inflammatory proteases (Glossmann & Reider, 2013; Lockwood, 2010; Logie et al., 2012; Sweeney et al., 2003; Thorne & Lockwood, 1991). Accordingly, our systematic chemoinformatics approach confirmed that metallopeptidases and proteins with transition metal ion‐binding properties were significantly overrepresented among the predicted targets for metformin. These findings lend weight to the notion that metal‐interactive regulation of protein functioning should be viewed as a central mechanism of action of metformin and, consequently, that zinc, copper, iron, and other metals might be viewed as primary targets of metformin. The metal‐binding properties of metformin appear to be involved not only in its capacity to regulate a number of proteases (Lockwood, 2010; Sweeney et al., 2003), but also to interact with and regulate the activity of a significant number of aging‐ and cancer‐related DNA/chromatin‐interacting enzymes. Interestingly, our systematic chemoinformatics approach coupled to confirmatory experimental testing revealed for the first time that metformin can directly target the chromatin‐modifying activity of aging‐related histone demethylases such as KMD6A/UTX.

The structural basis for metformin‐induced inhibition of H3 Lys27 demethylation might rely on its capacity to interact with the same set of residues that are known to fix the orientation of one of the methyl groups such that it can be subjected to demethylation in the catalytic pocket of KDM6A/UTX (Sengoku & Yokoyama, 2011). Indeed, we verified that an entire family of five pharmacologically relevant biguanides sharing a common functional group comprising two guanidiniums joined by a common nitrogen, targeted, albeit with different inhibitory potencies, the H3K27me3 demethylase activity of KDM6A/UTX. The greater similarity of metformin to the crystallographic ligand of KDM6A/UTX might explain how metformin discriminates KDM6A/UTX from KDM6B/JMJD3 to more potently and specifically inhibit the former. Beyond distortion of H3 binding, the metal‐binding properties of metformin might mimic the metal‐coordination functions of KDM6A/UTX and KDM6B/JMJD3 ligands, thereby disrupting the sequence‐specific demethylation mechanism of both proteins. In this regard, norMitoMet, a novel metformin derivative tagged with the mitochondrial vector triphenylphosphonium (TPP^+^), which has been shown to notably increase the antineoplastic efficacy of parental metformin (Boukalova et al., 2016), behaved as the most potent KDM6A/UTX inhibitor—in the low micromolar range—likely because it was the sole biguanide that shared with metformin the ability to coordinate with catalytic metal ions of KDM6A/UTX. Thus, the unique side chains that are known to determine the pharmacological differences of biguanides including mitochondrial accumulation and mCI inhibition (Boukalova et al., 2016; Bridges, Sirviö, Agip & Hirst, 2016; Bridges et al., 2014) might also influence the ability of the biguanides to interfere with KDM6A/UTX activity. Our discovery that biguanides do not exhibit either 2D structural‐topological analogies or 3D nonanalogous bioisosteric physicochemical relationships with KDM‐targeted metabolites (Mishur et al., 2016; Tarhonskaya et al., 2017) strongly suggest that different biguanides might be viewed as a new family of pharmacologically active KDM6A/UTX regulators.

The unforeseen capacity of biguanides to directly inhibit the demethylation activity of KDM6A/UTX might provide mechanistic support for recent studies showing that metformin efficiently prevents fatty acid/high‐fat diet‐induced metabolic memory (Tikoo, Sharma, Amara, Pamulapati & Dhawale, 2016), which is epigenetically driven by decreased abundance of H3K27me3 on the FOXO1 promoter (Kumar, Pamulapati & Tikoo, 2016), as well as the synergistic interactions between metformin and the KDM6 inhibitor GSK‐J4 in a panel of non‐small cell lung carcinoma cell lines (Watarai et al., 2016). However, one might argue that the in silico capacity of metformin to bind the catalytic site of KDM6A/UTX occurs with rather high binding energies, whereas metformin‐driven reduction in KDM6A/UTX enzymatic activity in vitro and global augmentation of the H3K27me3 mark in cellulo were obtained by applying high, nonphysiological millimolar concentrations in excess of the therapeutic levels achieved in human patients. However, it should be acknowledged that the in silico energy calculations can be explained by metformin being a very small molecule and by docking calculations being performed against cavities that, in most cases, are biased toward the ligand to which the target structure is cocrystallized. Despite its small size, however, metformin was capable of simultaneously interacting, in the crystal structure of KDM6A/UTX (3AVS), with the residues required not only for iron binding in the demethylase reaction but also with the methyl groups of the H3K27me3 side chain as well as NOG—an analog of the cofactor AKG (H1146, E1148, Y1135; Sengoku & Yokoyama, 2011; Shpargel, Sengoku, Yokoyama & Magnuson, 2012). Moreover, metformin‐induced inhibition of catalysis by isolated mCI, which is believed to be the primary target of metformin, requires even higher concentrations of metformin (i.e., 20–100 mmol/L; Bridges et al., 2014, 2016) than those required to inhibit the demethylation activity of the purified KDM6A/UTX enzyme. We experimentally confirmed that neither mCI nor AMPK seem to be required to elicit specific augmentation of the global levels of the KDM6A/UTX substrate H3K27me3 in mouse and human cells growing in the presence of millimolar concentrations of metformin. Wan et al. (2018) recently suggested that mCI inhibition by metformin might attenuate H3K27me3 via downstream activation of AMPK‐catalyzed inhibitory phosphorylation of EZH2, a core component of the H3K27me3 methyltransferase PRC2. The exacerbated augmentation of endogenous H3K27me3 occurring in mitochondria‐deficient (Rho 0) cells, in which the expected mCI (inhibition)/AMPK (activation) coupling cannot be established in response to metformin (Cuyàs et al., 2017), and the preliminary observation that biguanides might reduce H3K27me3 levels in KDM6A/UTX‐deficient cells compared to KDM6A/UTX‐proficient isogenic counterparts, favors a more complex regulation of the metformin‐regulated H3K27me3 status in which the output of the AMPK‐H3K27me3 methyltransferase EZH2 regulatory axis largely depends on the status of the mCI‐/AMPK‐independent KDM6A/UTX demethylase activity. Perhaps more importantly, we verified the physiological relevance of the in vitro capacity of metformin to inhibit the demethylase activity of KDM6A/UTX by assessing its capacity to promote H3K27me3 methylation in animals and in human‐derived samples when used at therapeutic dosages commonly employed to treat T2D, as a surrogate of metformin‐induced changes in KDM6A/UTX activity in vivo.

*Ldlr* ^*−/−*^ mice, a robust animal platform to assess the effect of nutritional changes in the context of subclinical chronic inflammation, exhibited elevated global levels of hepatic H3K27me3 when receiving metformin in their drinking water ad libitum to deliver 250 mg/kg body weight/day for more than 2 months. That metformin could not entirely restore the abundance of H3K27me3 occurring in the liver of HFD‐fed animals (Vella et al., 2013) might reflect the epigenetic incapacity of HFD‐damaged tissues to fully benefit from the expected response to metformin unless combined with diet reversal (Riera‐Borrull et al., 2017). At a dose of 250 mg kg^−1^ day^−1^ i.p., a route of administration that is equivalent to 1,200 mg/day metformin for a 60 kg individual (Reagan‐Shaw, Nihal & Ahmad, 2008) and yields plasma concentrations in the low micromolar range (Chandel et al., 2016; Dowling et al., 2016; Memmott et al., 2010; Menendez, Martin‐Castillo & Joven, 2016), metformin treatment resulted in a significant twofold reduction in the tumor volume in xenograft‐bearing mice highly enriched with tumor‐initiating cancer stem cells (CSC) (Cufi et al., 2012; Martin‐Castillo et al., 2013; Oliveras‐Ferraros et al., 2012) that was accompanied by a noteworthy twofold augmentation of global H3K27me3 in tumor tissues. Because decreased abundance of H3K27me3 is a predictor of cancer aggressiveness independently of the expression of the H3K27me3 methyltransferase EZH2 (Holm et al., 2012; Wei et al., 2008) closely related to the maintenance of poorly differentiated CSC (Sakaki et al., 2015; Yan et al., 2017), our findings suggest that metformin might exert a suppressive influence on the CSC phenotype by enhancing deposition of the repressive H3K27me3 mark. Nonetheless, the post‐treatment serum levels of circulating H3K27me3 were significantly higher in nondiabetic HER2‐positive breast cancer patients who received diabetic doses of metformin (850 mg twice a day, which yielded median values of metformin concentrations in blood in the range of 5–20 μmol/L—unpublished observations) for 6 months, in addition to standard‐of‐care neoadjuvant treatment. While it remains to be established whether the release of H3K27me3 into the circulation might directly reflect greater exchanges between activating/repressive epigenetics marks in target (e.g., hepatic and/or tumor) tissues, it was noteworthy that the significant augmentation of circulating H3K27me3 promoted by adding metformin to standard neoadjuvant treatment occurred solely in those cancer patients who achieved a complete tumor response in both the breast and axillary lymph nodes. Given that the release of post‐translational modifications of histone proteins from cells into the blood circulation has been suggested to mirror cell‐specific and disease‐related processes (Deligezer et al., 2010; Gezer et al., 2015; McAnena, Brown & Kerin, 2017), our findings might reveal a novel, noninvasive method to dynamically monitor the capacity of metformin to correct or reset the abnormal chromatin state of cancer and/or aging cells.

Changes in chromatin‐associated enzymes are beginning to be viewed as a key driving force in the remodeling of the epigenomic landscape of aging cells. Given the exquisite responsiveness of chromatin modifiers to metabolic cues, the metabolic control of healthspan should reasonably involve a similar regulation of the epigenome (López‐Otín et al., 2016). Our current findings suggest that the healthspan‐promoting capacity of metformin might involve not only an indirect regulation of metabolo‐epigenetic fluxes, but also a direct targeting of key aging/senescence‐related chromatin regulators such as the H3K27me3 histone demethylase KDM6A/UTX (Jin et al., 2011; Maures, Greer, Hauswirth & Brunet, 2011; McCauley & Dang, 2014; Shah et al., 2013). A decrease in repression‐associated H3K27me3 (and an increased activity of the H3K27me3 demethylase KDM6A/UTX) is a key feature of the global chromatin reconfiguration occurring not only in somatic cells during the normal aging process but also in prematurely aging cells in HGPS and WS (Scaffidi & Misteli, 2005; Shah et al., 2013; Shumaker et al., 2006). In addition, landmark observations in *Caenorhabditis elegans* have linked gain of H3K27me3 (and loss of the H3K27me3 demethylase UTX‐1) to extended longevity, strongly suggesting that preserving high levels of H3K27me3 by inhibiting KDM6/UTX may be critical for maintaining youthfulness (Jin et al., 2011; Maures et al., 2011; McCord et al., 2013; Shah et al., 2013). Despite conflicting data from different model systems, there is a trend for increases in activating histone marks (e.g., H3K4m2/3, H3K36me3) and decreases in repressive histone marks (e.g., H3K9m2/3, H3K27me3) indicative of a more actively transcribed genome, which is consistent with a well‐recognized open chromatin conformation in aging cells and organisms that culminates in the so‐called heterochromatin loss model of aging (Pal & Tyler, 2016). Metformin's ability to robustly restore the global levels of H3K27me3 in fibroblasts obtained from aged individuals or from patients with premature aging syndromes supports the notion that metformin could directly regulate the biological machinery of human aging by directly modifying aging‐associated histone mark changes and adds a new epigenetic dimension to the recent discovery of its capacity to alleviate the pathological defects of accelerated aging (Egesipe et al., 2016). Recent studies using naturally long‐lived and cancer‐resistant organisms have provided key clues to suggest that engineering more stable, H3K27me3‐enriched epigenomes might prevent cancer and extend human lifespan (Tan et al., 2017). Against this background, it might be tempting to suggest that metformin could drive phenotypes of cancer resistance and extended healthspan by promoting more resilient epigenomes that resist aging‐related loss of cell fate and dedifferentiation (Vazquez‐Martin et al., 2012). Yet it remains to be determined the relative weight of such direct effect of metformin to influence the epigenome in comparison with the widely accepted multifaceted capacity of metformin to indirectly target a number of aging‐related metabolic mechanisms (Barzilai et al., 2016), our mechanistic insights might be relevant not only to assist in deconvoluting the epigenetic mechanisms of action of metformin, but also in developing a new generation of KDM6A‐targeted biguanides.

We are currently lacking epigenetic treatments capable of specifically targeting the chromatin features that are most affected with age. Indeed, a major concern in future planning and therapeutic management of the aging epigenome would be the safety of epigenetic drugs that, when used in the long term and in patients with noncancer diseases, might result in significant side effects including the epigenetic promotion of malignancies. The present discovery that metformin operates as a selective regulator of chromatin‐modifying histone demethylase activities (e.g., KDM6A/UTX) and epigenetic changes that track with aging (e.g., H3K27me3) together with the extensive clinical experience with millions of type 2 diabetics who have been prescribed metformin, and its well‐characterized and modest toxicity profile, certainly presents a testable clinical scenario in which metformin or next‐generation metformin derivatives could directly regulate the biological machinery of aging per se by targeting core chromatin modifiers of the aging epigenome.

## 4 EXPERIMENTAL PROCEDURES

### 4.1 Virtual profiling

Virtual profiling was performed with the ligand‐ and structure‐based software tools (http://www.mindthebyte.com) Cabrakan (2D), Hurakan (3D), and Ixchel (3D target‐based), using the chemical structure of metformin as a seed. Cabrakan is a 2D ligand‐based VP tool that compares molecules through the use of 2D fingerprints and the assignment of biological activity with an internal algorithm. Hurakan is a 3D VP tool that compares a query molecule with the structures present in the reference database using CoMSIA fields on a 3D grid. Hurakan can compare molecules according to their relationship with their environment, thus obtaining biomimetic compounds with different structures. Cabrakan and Hurakan employ an in‐house modified version of chembl as reference database, which contains around 1,300,000 chemical compounds with detailed information including target data. Cabrakan and Hurakan can therefore predict the biological profile of a query molecule based on 2D and 3D similarity. A target was counted once when it appeared as both 2D and 3D hit during ligand‐based VP experiments. Ixchel is a structure‐based VP tool that performs docking calculations of a molecule (SDF or SMILE file) against an in‐house developed database comprising almost 9,000 binding sites protein cavities curated from RSCB PDB according to UniProtKB human entries. Ixchel returns the binding energy of every possible interaction, which allows the classification and prediction of the targets.

### 4.2 Structure modeling

The metformin structure was modeled from the chembl database using the chembl 1431 entry. All predicted targets were modeled from their crystal structures with the exception of those for which structures were not available, in which case homology modeling was carried out using SWISS‐MODEL.

### 4.3 Docking calculations

All docking calculations were performed using Itzamna and Kin (http://www.mindthebyte.com), classical docking, and blind‐docking software tools. Protein structures from RSCB PDB were directly employed for docking calculations when crystallographic ligands were available; otherwise, blind‐docking calculations involving cavity searching and best cavity selection were performed to allow further studies (MD simulations). When crystal structures exhibited more than one ligand, all of them were considered. Two runs were carried out for each calculation to avoid false positives.

### 4.4 Molecular dynamics calculations

Short (1 ns) MD simulations were performed using namd version 2.10 over the best‐docked complexes, which were selected based on the interaction energy. The Ambers99SB‐ILDN and the GAFF force‐field set of parameters were employed for receptors and ligands, respectively. The ligand parameters were obtained using Acpype software, whereas the receptor structures were modeled using the leap module of Amber Tools. Simulations were carried out in explicit solvent using the TIP3P water model with the imposition of periodic boundary conditions via a cubic box. Electrostatic interactions were calculated by the particle‐mesh Ewald method using constant pressure and temperature conditions. Each complex was solvated with a minimum distance of 10 Å from the surface of the complex to the edge of the simulation box. Na^+^ or Cl^−^ ions were also added to the simulation to neutralize the overall charge of the systems. The temperature was maintained at 300 K using a Langevin thermostat, and the pressure was maintained at 1 atm using a Langevin Piston barostat. The time step employed was 2 fs. Bond lengths to hydrogens were constrained with the SHAKE algorithm. Before production runs, the structure was energy minimized followed by a slow heating‐up phase, using harmonic position restraints on the heavy atoms of the protein. Subsequently, the system was energy minimized until volume equilibration followed by the production run without any position restraints.

### 4.5 Molecular Mechanics/Generalized Born Surface Area

Molecular mechanics‐generalized born surface area rescoring was performed using the MMPBSA.py algorithm within AmberTools. The snapshots generated in the 1 ns MD simulation were imputed into the postsimulation MM/GBSA calculations of binding free energy. Graphical representations were prepared using vmd version 1.9.1, LigPlot^+^ and plip version 1.3.0.

See Supporting Information for full description of details concerning cell lines, enzymatic assays, determination of histone modifications, and molecular similarity searches.

## AUTHOR CONTRIBUTIONS

J.A.M. and M. S‐M. conceived the idea, directed the project, and wrote the manuscript. L. L‐P., M. S‐M., and A. N‐C. performed virtual profiling, docking and molecular dynamics calculations, and Molecular Mechanics‐Generalized Born/Surface Area scoring. B. M‐C, B. V., J. S., L. W., and J. N. provided essential materials necessary for the study. E. C., S. F‐A., S. V., B. M‐C., and J. J. examined the chemoinformatics data, provided intellectual insight, and critically read the manuscript. F. L‐M., N. C., and P. N‐R. performed experiments involving *Ldlr* ^*−/−*^ animals. E. C. and S. V. were involved in the design, development, and analysis of all the cell‐based, enzymatic, and xenograft tissue experiments.

## CONFLICT OF INTEREST

The authors declare that they have no conflict of interest.
