# Supplementary material for: Metformin directly targets the H3K27me3 demethylase KDM6A/UTX
Source: Aging Cell. 2018 May 8;17(4):e12772. doi: 10.1111/acel.12772 (PMC6052472; doi:10.1111/acel.12772)
Supplement: Supplementary file 1 [file ACEL-17-na-s001.docx]

**Metformin directly targets the**

**H3K27me3 demethylase KDM6A/UTX**

**SUPPORTING INFORMATION**

**SUPPLEMENTARY MATERIALS AND METHODS**

**Cell lines.** For the preparation of ρ0 cells, the 4T1 parental tumor cell line was cultured for 10–12 weeks in low-dose EtBr (50–100 ng/mL) supplemented with 1 mmol/L pyruvate and 50 μg/mL uridine, followed by transfer to medium lacking EtBr as described previously (Tan et al., 2015). SV40 T-antigen-immortalized *ampk^+/+^* and *ampk^-/-^* MEFs, the latter bearing a double knockout of the α1 and α2 subunits of AMPK, were generated as described previously (Laderoute et al., 2006). Normal skin primary fibroblasts (GM00038, AG09602), HGPS skin primary fibroblasts (AF11498), and WS skin primary fibroblasts (AG06300, AG04110) were obtained from Coriell Cell Repository (NJ, USA).

**Enzymatic assays.** The enzymatic studies of recombinant human CBX1 (BPS#50012, lot#121101-D), GCN5 (BPS#50074, lot#140423), KDM4C/JMJD2C (BPS#50105, lot#121107), KDM6A/UTX (BPS#50116, lot#150805-A), and KDM6B/JMJD3 (BPS#50115, lot#140925) were outsourced to BPS Bioscience. For AlphaScreen format assays, all of the enzymatic reactions were performed in duplicate at room temperature (RT) for 60 minutes in a 10 μL mixture containing assay buffer, histone H3 peptide substrate or histone substrate, enzyme, and metformin or the reference compounds. The 10 μL reactions were carried out in 384-well Optiplates (PerkinElmer). The serial dilution of the compounds was first performed in either assay buffer (CBX1) or 3.3% DMSO/assay buffer (KDM4C/JMJD2C, KDM6A/UTX, KDM6B/JMJD3). From this step, 3 μL of compound (2.5 μL in the case of CBX1) is added to 4 μL (2.5 μL in the case of CBX1) of enzyme and is incubated for 30 minutes at RT. After this incubation, 3 μL of substrate (5 μL in the case of CBX1) is added to initiate the reaction. The final DMSO concentration is 1%. After enzymatic reactions, 5 μL of anti-mouse acceptor beads (PerkinElmer, diluted 1:500 with 1× detection buffer; 10 μL of GSH-acceptor beads diluted 1:250 with 1× detection buffer in the case of CBX1) or 5 μL of anti-rabbit acceptor beads (PerkinElmer, diluted 1:500 with 1× detection buffer) and 5 μL of primary antibody (BPS#52140E,F) were added to the reaction mix. After brief shaking, the plate was incubated for 30 minutes. Finally, 10 μL of AlphaScreen streptavidin-conjugated donor beads or Nickel Chelate Alpha Donor beads (Perkin, diluted 1:125 with 1× detection buffer) were added. After 30 minutes (10 minutes in the case of CBX1), the samples were measured in the AlphaScreen microplate reader (EnSpire Alpha 2390 Multilabel reader, PerkinElmer).

For AlphaLISA assays, all of the enzymatic reactions were conducted in duplicate at RT for 60 minutes in a 50 μL mixture containing assay buffer, enzyme (KDM6A/UTX, KDM6B/JMJD3), and the test compound. The 50 μL reactions were carried out in wells of a substrate pre-coated plate. The final DMSO concentration was 1% in all wells.

For GCN5 HAT assays, the enzymatic reactions were conducted in duplicate at 37 ^o^C for 30 minutes in a 50 μL mixture containing 50 mmol/L HEPES, pH 7.4, 0.08% Triton-X100, 100 μmol/L acetyl-CoA (Sigma-Aldrich #A2056), 50 μmol/L Histone H3 peptide (1-21; BPS#52010), GCN5 enzyme, and the test compound. After enzymatic reactions, 50 μL of stop solution was added to stop the reaction, followed by addition of 100 μL of developer solution to each well. The reaction mixtures were incubated at RT for 10 minutes. Fluorescence intensity was measured at an excitation of 360 nm and an emission of 460 nm using a Tecan Infinite M1000 microplate reader.

The alpha-screen/fluorescent intensity data were analyzed and compared using Graphpad Prism software (GraphPad Software Inc.). In the absence of either metformin or reference compounds, the A-screen or fluorescent intensity (F_t_) in each data set was defined as 100% activity. In the absence of enzyme, the intensity (F_b_) in each data set was defined as 0% activity. The percent activity in the presence of each compound was calculated according to the following equation: %activity = (F-F_b_)/(F_t_-F_b_), where F=the A-screen or fluorescent intensity in the presence of the compound. The values of % activity versus a series of compound concentrations were then plotted using non-linear regression analysis of a sigmoidal dose-response curve generated with the equation Y=B+(T-B)/1+10^((LogEC50-X)×Hill Slope)^, where Y=percent activity, B=minimum percent activity, T=maximum percent activity, X= logarithm of compound and Hill Slope=slope factor or Hill coefficient. The IC_50_ value was determined as the concentration causing a half-maximal percent activity.

**Histone modifications.** The study of H3 methylation using the Luminex Histone H3 post-translational modification (PTM) assay was outsourced to Active Motif. Briefly, cells were incubated in the absence or presence of metformin (48 h) and then samples were subjected to a two-step lysis procedure in which the cell pellet was suspended in a hypotonic buffer containing Nonidet NP-40 for 30 minutes on ice. Nuclei were pelleted and acid extraction was performed for two hours at 4 ^o^C. Cellular debris was pelleted, and lysate aliquots were frozen in a methanol-dry ice bath and stored at -80 ^o^C until testing. Protein concentrations (A280) were determined and multiplex assays were performed with beads grouped according to relative abundance. Beads for H3 Total, H3K9me1, H3K9me2, H3K9me3, H3K27me3, H3K27me2 and H3K26me3 were multiplexed, while H3 Total, and H3K4me3 were tested as a separate multiplex. Samples were tested in duplicate using three amounts/sample, read in a Magpix instrument and data exported as CSV files. Data sets with equivalent H3 Total signals were selected for downstream analysis. Net median fluorescent Intensity (Net MFI) associated with each PTM-specific bead was expressed as a ratio relative to Histone H3 Total signals for each well. Ratio values were averaged for each sample input amount and percent change in the ratio relative to the reference samples was determined. A control lysate was also included to verify successful execution of the multiplex assay.

For western blot analyses of H3K27me3 and total H3, histones were extracted using the EpiQuik Total Histone Extraction Kit (Epigentek, Brooklyn, NY, USA); 1 μg of purified histones were electrophoresed on 17% SDS-PAGE gels, transferred to nitrocellulose membranes, and incubated with antibodies against histone H3 (Abcam; cat.#ab1791, 1:5,000 dilution) and H3K27me3 (Upstate-Millipore; cat.#07-449, 1:2,000 dilution), followed by horseradish peroxidase-conjugated secondary and chemilumiscence detection.

For ELISA-based analyses of H3K27me3 and total H3, histones were extracted from frozen mouse tissue (liver), using the EpiQuik Total Histone Extraction Kit (Epigentek); 100 ng of total histone extracts were analyzed in duplicate using the EpiQuik Global Tri-methyl Histone H3-K27 and EpiQuik Total Histone H3 Quantification ELISA Kits (EpiGentek). Details of animals, animal care, dietary details, and metformin provision have been described previously (Riera-Borrull et al., 2017).

Profiling of circulating H3K27me3 and total H3 levels was performed on pre- and post-treatment serum obtained from women with primary, non-metastatic HER2-positive breast cancer who were randomized (1:1) to receive metformin (850 mg twice-daily) for 24 weeks concurrently with 12 cycles of weekly paclitaxel plus trastuzumab, followed by four cycles of 3-weekly fluorouracil, epirubicin, cyclophosphamide plus trastuzumab (arm A), or equivalent regimen without metformin (arm B), followed by surgery (Martin-Castillo et al., 2010; Pernas et al., 2017). The study (METTEN-01 trial) was registered with the EU Clinical Trials Register (EudraCT number 2011-000490-30).

Thirty μl of serum samples were analyzed in duplicate using the EpiQuik Circulating Trimethyl Histone H3K27 and EpiQuik Circulating Total Histone H3 Quantification ELISA Kits (EpiGentek).

**Molecular similarity.** 2D and 3D molecular similarity calculations (Eckert and Bajorath, 2007; Freitas et al., 2010; Maggiora et al., 2014; Nigsch and Mitchell, 2008; Willlett, 2006) and heatmap representations were performed using RDKit (RDKit: Open-Source Cheminformatics Software; <http://www.rdkit.org>) and Searbon (seaborn: statistical data visualization, v.0.8.1. https://seaborn.pydata.org), respectively.

**BIBLIOGRAPHY**

Eckert H, Bajorath J (2007) Molecular similarity analysis in virtual screening: foundations, limitations and novel approaches. *Drug Discovery Today* **12**, 225-233.

[Freitas RF](https://www.ncbi.nlm.nih.gov/pubmed/?term=Freitas%20RF%5BAuthor%5D&cauthor=true&cauthor_uid=20055489), [Bauab RL](https://www.ncbi.nlm.nih.gov/pubmed/?term=Bauab%20RL%5BAuthor%5D&cauthor=true&cauthor_uid=20055489), [Montanari CA](https://www.ncbi.nlm.nih.gov/pubmed/?term=Montanari%20CA%5BAuthor%5D&cauthor=true&cauthor_uid=20055489) (2010) Novel application of 2D and 3D-similarity searches to identify substrates among cytochrome P450 2C9, 2D6, and 3A4. [*J Chem Inf Model*.](https://www.ncbi.nlm.nih.gov/pubmed/?term=Freitas+RF%2C+Bauab+RL),**50**, 97-109.

Maggiora G, Vogt M, Stumpfe D, Bajorath J (2014) [Molecular similarity in medicinal chemistry.](https://www.ncbi.nlm.nih.gov/pubmed/24151987) *J Med Chem*., **57**, 3186-3204.

Martin-Castillo B, Dorca J, Vazquez-Martin A, Oliveras-Ferraros C, Lopez-Bonet E, Garcia M, Del Barco S, Menendez JA (2010) [Incorporating the antidiabetic drug metformin in HER2-positive breast cancer treated with neo-adjuvant chemotherapy and trastuzumab: an ongoing clinical-translational research experience at the Catalan Institute of Oncology.](https://www.ncbi.nlm.nih.gov/pubmed/19884247) *Ann Oncol.,* **21**, 187-189.

Nigsch F, Mitchell JB (2008) How to winnow actives from inactives: introducing molecular orthogonal sparse bigrams (MOSBs) and multiclass Winnow. *J Chem Inf Model*., **48**, 306-318.

Riera-Borrull M, García-Heredia A, Fernández-Arroyo S, Hernández-Aguilera A, Cabré N, Cuyàs E, Luciano-Mateo F, Camps J, Menendez JA, Joven J (2017) [Metformin Potentiates the Benefits of Dietary Restraint: A Metabolomic Study.](https://www.ncbi.nlm.nih.gov/pubmed/29143783) *Int J Mol Sci.* **18**, pii: E2263.

Willett P (2006) [Similarity-based virtual screening using 2D fingerprints.](https://www.ncbi.nlm.nih.gov/pubmed/17129822) *Drug Discov Today* **11**, 1046-1053.

**Collaborators (METTEN-01 clinical trial investigators):** Sonia Pernas (Department of Medical Oncology, Breast Unit, Catalan Institute of Oncology-Hospital Universitari de Bellvitge-Bellvitge Research Institute (IDIBELL), L’Hospitalet de Llobregat, Barcelona, Spain), Joan Dorca (Medical Oncology, Catalan Institute of Oncology, Girona, Spain), Isabel Álvarez-López (Medical Oncology Service, Hospital Donostia, Donostia-San Sebastián, Spain), Susana Martínez (Medical Oncology Department, Hospital de Mataró, Mataró, Barcelona, Spain), Jose Manuel Pérez-García (Instituto Oncológico Baselga (IOB), Hospital Quiron, Barcelona, Spain), Norberto Batista López (Medical Oncology Service, Hospital Universitario de Canarias, La Laguna, Tenerife, Spain), César A. Rodríguez-Sánchez (Instituto de Investigación Biomédica de Salamanca (IBSAL), Salamanca, Spain; ^1^Medical Oncology Service, Hospital Universitario de Salamanca, Salamanca, Spain), Kepa Amillano (Medical Oncology, Sant Joan de Reus University Hospital, Reus, Spain), Severina Domínguez Fernández (Medical Oncology Service, Hospital de Txagorritxu, Vitoria-Gasteiz, Spain), Maria Luque-Cabal (Department of Medical Oncology, Hospital Universitario Central de Asturias, Oviedo, Spain), Idoia Morilla (Department of Medical Oncology, Breast Unit, Catalan Institute of Oncology-Hospital Universitari de Bellvitge-Bellvitge Research Institute (IDIBELL), L’Hospitalet de Llobregat, Barcelona, Spain), Gemma Viñas (Medical Oncology, Catalan Institute of Oncology, Girona, Spain), and Javier Cortés (Department of Medical Oncology, Ramón y Cajal University Hospital, Madrid, Spain).

**SUPPLEMENTARY FIGURES**

**Figure S1**

**
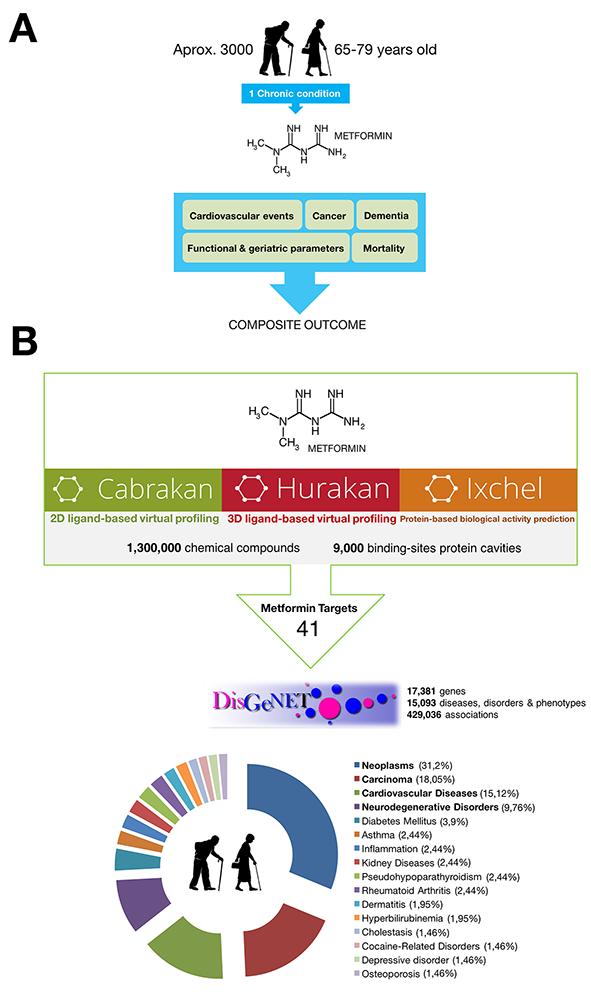
**

**Figure S1. A. Targeting Aging with Metformin (TAME): A first-in-class study to target aging in humans.** Metformin has been used successfully to treat type 2 diabetes (T2DM) for over 60 years with an exceptional safety record. Metformin has been shown to delay onset of T2DM, and in those individuals with T2DM its usage has been associated with lower rates of cancer, cardiovascular disease, all-cause mortality, and possibly less cognitive decline. TAME is a placebo controlled, multi-center study in approximately 3,000 elderly persons aged 65–79, with a novel primary outcome of delaying the incidence of a composite of multiple age-related diseases (rather than each one separately) and other geriatric syndromes (Barzilai et al., 2016). By targeting fundamental facets of aging biology, TAME researchers hope to delay or prevent the onset of all age-related diseases simultaneously, rather than treat each one individually as they arise and accumulate. **B. Chemoinformatics de-orphanization of metformin targets.** *Top.* Metformin was initially used as a seed to run 2D and 3D ligand-based and structure-based virtual profiling (VP) experiments using proprietary software tools (Cabrakan, Hurakan, and Ixchel; [www.mindthebyte.com)](http://www.mindthebyte.com)). The reference databases contained around 1,300,000 chemical compounds with detailed information including target data as well as more than 9,000 well-characterized binding-site protein cavities. We selected 28 (10 from 2D and 18 from 3D) and 13 top-ranked VP hits from the ligand-based and the structure-based VP approaches, respectively. *Bottom.* The DisGeNET discovery platform ([www.disgenet.org)](http://www.disgenet.org)), a relational database that integrates gene-disease associations from curated databases and the literature, and additional information such as pathways and SNPs, was employed to associate the chemoinformatically-discovered metformin biomolecular targets with human diseases (**Table S4**). UniprotID O95050 was the sole target for which no associated disease could be found in DisGeNET.

**Figure S2.**

**
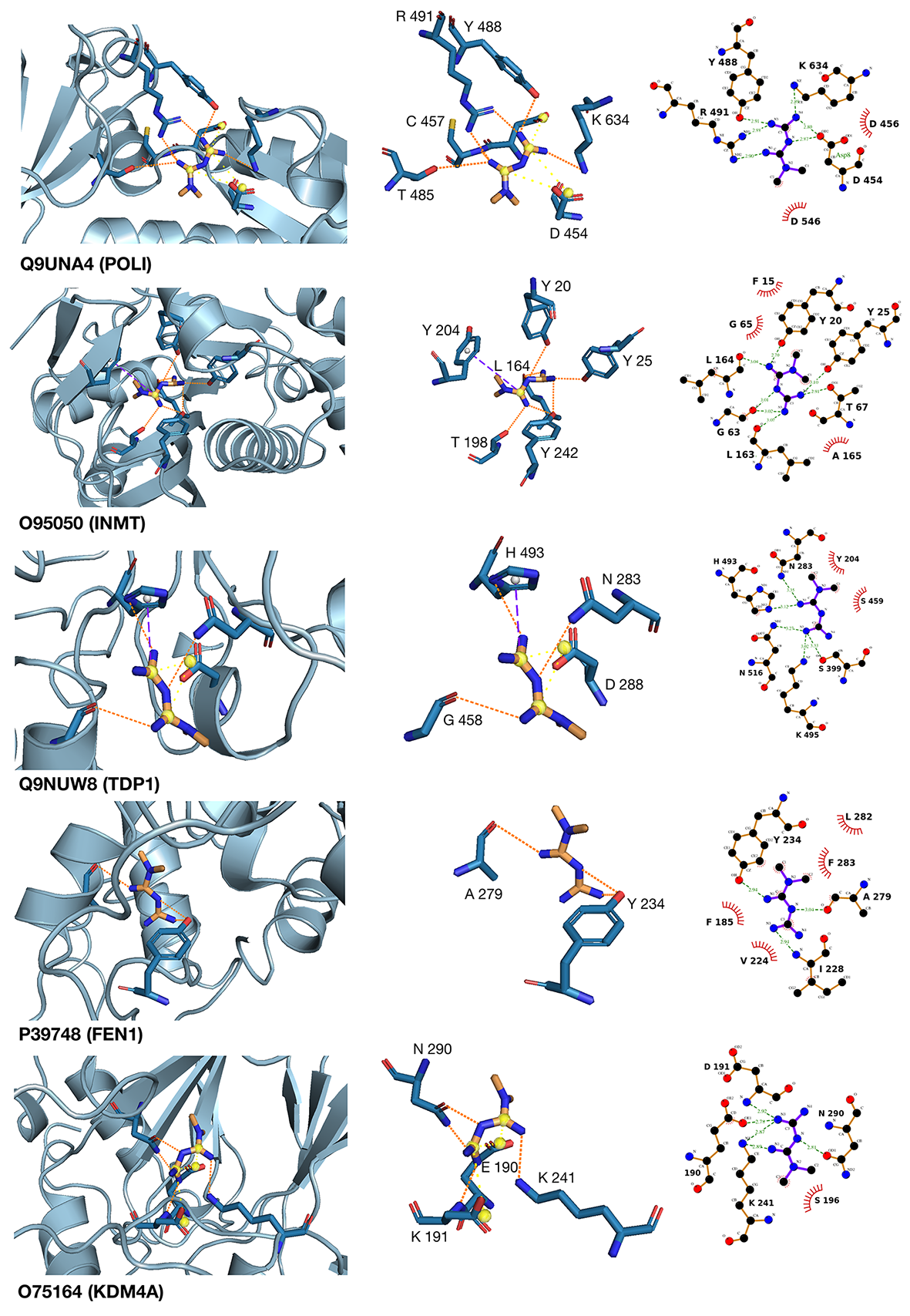
**

**Figure S2 (cont.)**

**
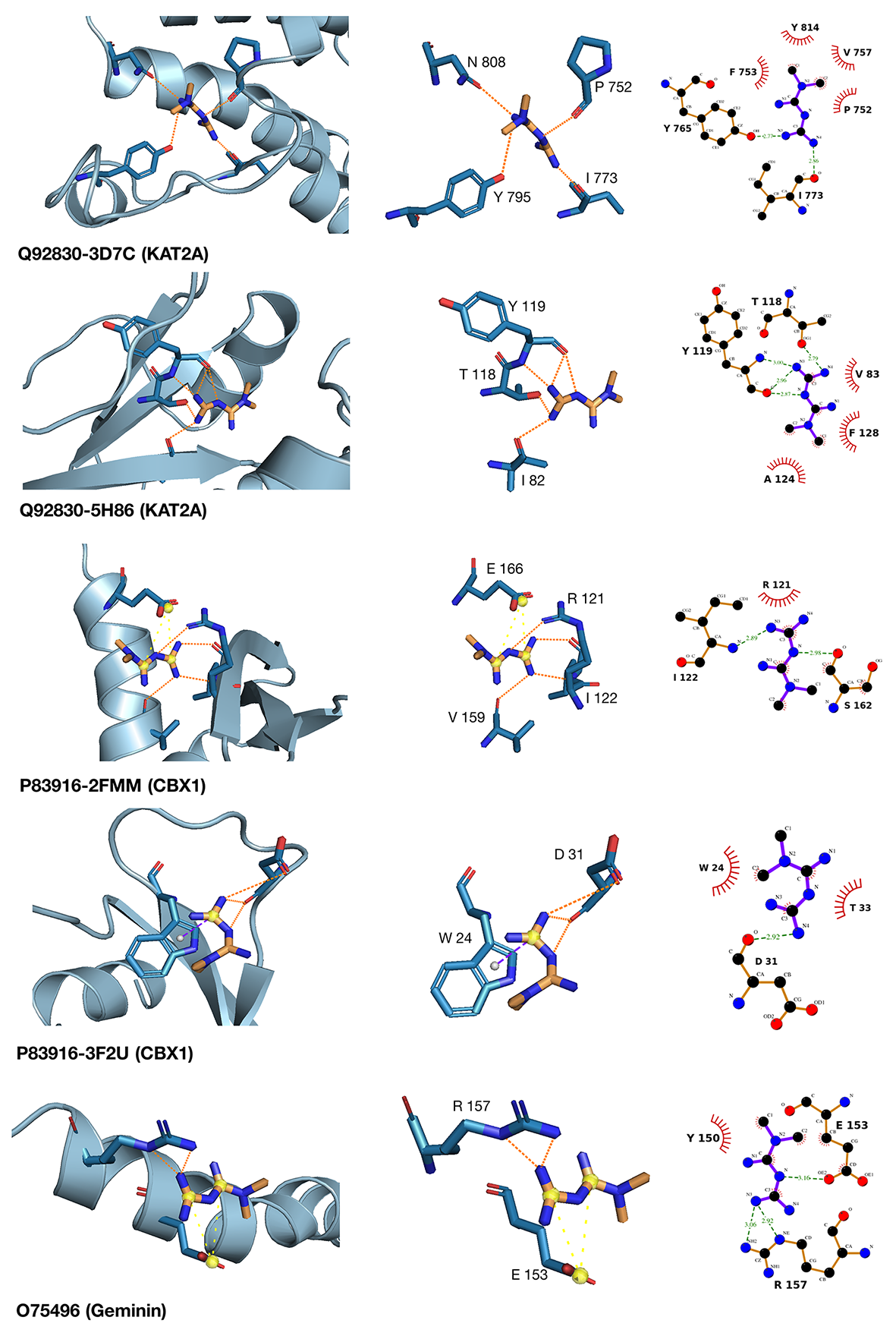
**

**Figure S2. Structural analysis of metformin binding to DNA/histone-interacting targets.** *Left panels* show in sticks the interaction residues between metformin and each target represented in the cartoon using PLIP. *Middle panels* show all the residues involved in metformin binding after MD simulation using PLIP. Hydrogen bond interactions are represented by orange dashed lines; salt bridges are represented by yellow dashed lines and charge centers by yellow spheres; Cation-π interactions are represented by blue dashed lines and white spheres represent the center of the aromatic ring. *Right panels* show all the residues involved in metformin binding after MD simulation using LigPlot^+^. Large spoked arcs represent protein residues making non-bonded contacts with the ligand whereas the small spoked arcs around atoms correspond to those of the ligand involved in these interactions; green dashed lines represent hydrogen bonds and their length is highlighted in green. Differences in the residues observed between central and right panels are easily explained since each software tool incorporates different distances schema that ultimately define which residues or interactions are shown. Thus, the combination of both observations gives a wider and complete view of metformin binding. The residue numbers shown correspond to the original PDB file numbering.

**POLI** (DNA polymerase iota)**:** Metformin is placed over the bottom portion of the thymidine-5’-triphosphate, the crystallographic ligand of PDB ID 1T3N, specifically over the phosphates with which metformin shares the interacting residues. Remarkably, phosphates are coordinating Mg^2+^, suggesting that metformin might share a similar role, further highlighting its metal-binding character. Metformin is stabilized by hydrogen bond interactions established with residues C457, T485, Y488, R491, K634 and T485. It should be noted that there are 3 salt bridge interactions between D454 and K634 and metformin, as well as two hydrophobic contacts established with D456 and D546 that might help metformin bind to POLI.

**INMT** (Indolethylamine N-methyltransferase)**:** The interaction analysis shows that the metformin binding site is placed at the bottom part of the cavity defined by S-adenosylhomocysteine (SAH), the crystallographic ligand of PDBID 2A14, specifically over the carboxyl and amino terminal groups. Of note, most of the key residues involved in this SAH pocket region (Y20, Y25, G63, L163 and L164) are shared by metformin, which is hydrogen bonded to L164, Y20, Y25, Y242, T198, T67, L163, and G63. Moreover, metformin establishes a π-cation interaction with Y204 as well as hydrophobic interactions with F15, G65, and A165.

**TDP1** (tyrosyl-DNA phosphodiesterase 1)**:** N283, H493, K495 and N516 are the key residues related to DNA cleavage in TDP1. Metformin binds to the cavity where DNA cleavage takes place by sharing all of these residues. Metformin is hydrogen bonded to N283, G458 and H493, S399, K495, and N516. Metformin additionally establishes a π-cation interaction with H493 as well as hydrophobic contacts with Y204 and S459.

**FEN1** (flap endonuclease 1)**:** The binding mode of metformin to the active site of FEN1 shares only one residue, Y234. Because such an interaction relates to the Mg^2+^ ion, the ability of metformin to coordinate with Mg^2+^ highlights its metal-interacting nature. Metformin is hydrogen bonded to Y234, A279, and I228 and establishes hydrophobic contacts with L282, F283, F185, and V224.

**KDM4A** (lysine demethylase): Metformin is placed over the bottom part of the cavity defined by N81 (3-carboxy-2,3-dideoxy-D-erythro-pentaric acid), the crystallographic ligand of the PDBID 5FY8, near where the histone binds. With the exception of S288, metformin shares all the residues involved in the binding mode of N81. Metformin is stabilized inside the binding pocket by hydrogen bonds with N290, D191, E190, and K241 as well as by establishing salt bridges with D191 and E190 and hydrophobic contacts with S196. Because N81 coordinates Ni^2+^, the emplacement of metformin suggests a similar role, highlighting its metal-interacting role.

**KAT2A/GCN5** (histone acetyltransferase)**:** Metformin is placed over and shares most of the residues of the acetyl-lysine-binding site of bromodomain-containing GCN5 (PDB ID 3D7C). Metformin is hydrogen bonded to Y765, N808, P752 and I773 and additionally shows hydrophobic contacts with F753, V757, and Y814. Metformin is also placed over the acyl-CoA N-acyltransferase domain of GCN5 (according to Interpro definition) at the cavity defined by butyryl coenzyme A (BCO), the crystallographic ligand of PDB ID 5H86 but without sharing any residue of the BCO binding mode. None of the residues identified as coenzyme A binding are shared by metformin. Metformin is placed near C79, the key residue for CoA binding. Metformin is hydrogen bonded to I82, T118, and Y119 and establishes hydrophobic contacts with V83, A124, and F128. The translation of the amino acid positions from the PDB to the actual amino acid numbers (I576, T612, Y613, V577, A618 and F622) suggests that metformin by some means binds around the CoA binding pocket without entering it. The fact that BCO is an extremely large ligand that defines a large cavity might explain why a small molecule such as metformin cannot be placed inside the CoA binding pocket. Accordingly, metformin prefers the acetylysine binding pocket rather than the CoA binding site in the KAT2A protein.

**CBX1** (chromobox protein homolog 1)**:** Metformin binds to the C-terminal chromo shadow domain (CSD) by interacting with the region where CSD interacts with other chromatin-associated nonhistone proteins such as ENT1 (PDB ID 2FMM); such binding occurs through residues that are near to but are not involved in these protein:protein interactions. Metformin shares the I221 and E166 binding residues with the CSD-B1 region that interacts with CSD-A1. Metformin is hydrogen bonded to V159, I122, E166, R121, and S162. Metformin is bound also to the histone-binding site (PDB ID 3F2U), where the N-terminal part of the H3 acetylated histone is recognized by CBX1. Two of the three residues involved in the metformin-binding mode are shared with the pocket. Metformin is hydrogen bonded to D31 and establishes hydrophobic interactions with T33. According to PDB numbering, metformin shows a π-cation interaction with W24. The translation of the amino acid positions from the PDB to the actual amino acid numbers suggests that the interaction of metformin is stabilized via D49, T51, and W42.

**GEMININ:** Metformin binds to the region of interaction between geminin and CDT1 (chromatin licensing and DNA replication factor 1). Metformin shares E153, Y150, and R157 in the geminin:CDT1 tertiary interaction interface. Metformin is hydrogen bonded to R157 and E154 and establishes two salt bridge interactions with E153 as well as hydrophobic contacts with Y150.

**Figure S3**


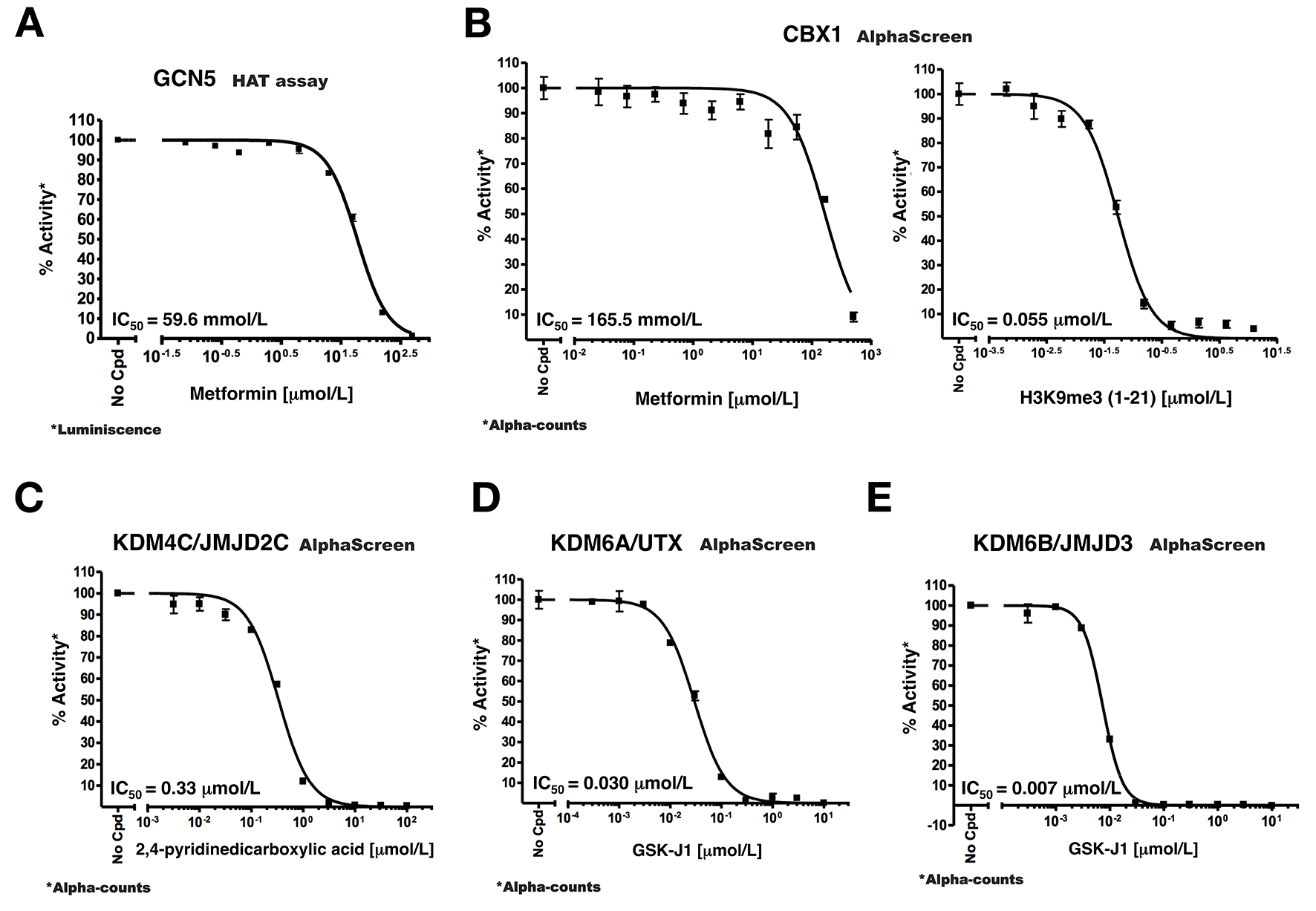


**Figure S3. Profiling of GCN5, CBX1, KDM4C/JMJD2C, KDM6A/UTX, and KDM6B/JMJD3 enzymatic activities.** The ligand-based target list for metformin contained several chromatin-modifying epigenetic regulators including histone acetyltransferase GCN5/KAT2A, a member of the SAGA acetylation complex related to mitochondrial respiratory metabolism and the histone methylation-related factor chromobox protein homolog 1 (CBX1), a key component of chromatin-associated complexes recognizing repressive epigenetic marks such as histone H3 trimethylation of lysine 9 (H3K9me3). To validate such predictions of the *in silico* screening, we performed GCN5/KAT2A and CBX1 enzyme activity assays in the presence of escalated concentrations of metformin. In a standard histone acetyltransferase (HAT) assay using acetyl-CoA and a 20-residue histone H3 peptide, the inhibitory concentration 50% (IC_50_) of metformin against KAT2A (GCN5) was 59.6 mmol/L. In an amplified luminescent proximity homogeneous assay (AlphaScreen), the IC_50_ of metformin against the epigenetic reader CBX1, however, was as high as 165.5 mmol/L, an inhibitory concentration notably higher than that observed with the H3K9(1–21)me3 peptide. Figure shows dose-response curves of GCN5 (**A**), CBX1 (**B**), KDM4C/JMJD2C (**C**), KDM6A/UTX (**D**), and KDM6B/JMJD3 (**E**) activities were created with Graphpad Prism by plotting luminescence/AlphaScreen signals as the function of metformin, H3K9me3 (1–21) peptide, 2,4-pyridinedicarboxylic acid, and GSK-J1 concentrations. The IC_50_ of the life-prolonging α-ketoglutarate (AKG) mimetic 2,4-pyridinedicarboxylic acid (2,4-PDCA; Mishur et al., 2016) was 0.33 μmol/L. GSK-J1, a competitive inhibitor of the AKG and Fe^2+^ cofactors but not of the substrate in jumonji demethylases (Kruidenier et al., 2012; Heinemann et al., 2014), was found to potently inhibit KMD6A/UTX and KDM6B/JMJD3 activities (IC_50_ values = 30 and 7 nmol/L, respectively).

**BIBLIOGRAPHY**

Mishur RJ, Khan M, Munkácsy E, Sharma L, Bokov A, Beam H, Radetskaya O, Borror M, Lane R, Bai Y, Rea SL (2016) [Mitochondrial metabolites extend lifespan.](https://www.ncbi.nlm.nih.gov/pubmed/26729005) *Aging Cell*. **15**, 336-348.

Kruidenier L, Chung CW, Cheng Z, Liddle J, Che K, Joberty G, Bantscheff M, Bountra C, Bridges A, Diallo H, Eberhard D, Hutchinson S, Jones E, Katso R, Leveridge M, Mander PK, Mosley J, Ramirez-Molina C, Rowland P, Schofield CJ, Sheppard RJ, Smith JE, Swales C, Tanner R, Thomas P, Tumber A, Drewes G, Oppermann U, Patel DJ, Lee K, Wilson DM (2012) [A selective jumonji H3K27 demethylase inhibitor modulates the proinflammatory macrophage response.](https://www.ncbi.nlm.nih.gov/pubmed/22842901) *Nature* **488**, 404-408.

[Heinemann B](https://www.ncbi.nlm.nih.gov/pubmed/?term=Heinemann%20B%5BAuthor%5D&cauthor=true&cauthor_uid=25279926), [Nielsen JM](https://www.ncbi.nlm.nih.gov/pubmed/?term=Nielsen%20JM%5BAuthor%5D&cauthor=true&cauthor_uid=25279926), [Hudlebusch HR](https://www.ncbi.nlm.nih.gov/pubmed/?term=Hudlebusch%20HR%5BAuthor%5D&cauthor=true&cauthor_uid=25279926), [Lees MJ](https://www.ncbi.nlm.nih.gov/pubmed/?term=Lees%20MJ%5BAuthor%5D&cauthor=true&cauthor_uid=25279926), [Larsen DV](https://www.ncbi.nlm.nih.gov/pubmed/?term=Larsen%20DV%5BAuthor%5D&cauthor=true&cauthor_uid=25279926), [Boesen T](https://www.ncbi.nlm.nih.gov/pubmed/?term=Boesen%20T%5BAuthor%5D&cauthor=true&cauthor_uid=25279926), [Labelle M](https://www.ncbi.nlm.nih.gov/pubmed/?term=Labelle%20M%5BAuthor%5D&cauthor=true&cauthor_uid=25279926), [Gerlach LO](https://www.ncbi.nlm.nih.gov/pubmed/?term=Gerlach%20LO%5BAuthor%5D&cauthor=true&cauthor_uid=25279926), [Birk P](https://www.ncbi.nlm.nih.gov/pubmed/?term=Birk%20P%5BAuthor%5D&cauthor=true&cauthor_uid=25279926), [Helin K](https://www.ncbi.nlm.nih.gov/pubmed/?term=Helin%20K%5BAuthor%5D&cauthor=true&cauthor_uid=25279926) (2014) Inhibition of demethylases by GSK-J1/J4. *Nature* **514**, E1-2.

**SUPPLEMENTARY TABLES**

| **Table S1. Docking binding energies of metformin against selected targets** | |  |  |  |
| --- | --- | --- | --- | --- |
|  |  | **Ligand/** | **Binding Energy** | **Binding Energy** |
| **Uniprot ID** | **PDB ID*** | **Cavity ID** | **(kcal/mol) R0^±^** | **(kcal/mol) R1^±^** |
| **Q9Y3Q0** | 3FED *Glutamate carboxypeptidase III* (**NAALAD2, GCPIII**) | BIX | -6.5 | -6.5 |
| **Q16769** | 3PBB *Glutaminyl-peptide cyclotransferase* (**QPCT**) | PBD | -6.3 | -6.3 |
| **P00491** | 3PHB *Purine nucleoside phosphorylase* (**PNP**) | IM5 | -4.2 | -3.9 |
| **P05089** | 3GN0 *Arginase-1* (**ARG1**) | DMO | -6.1 | -6.1 |
| **P27708** | 4C6E *CAD protein* (**CAD**) | NCD | -6.1 | -6.1 |
| **P42262** | 2XH *Glutamate receptor 2* (**GRM2**) | 7T9 | -4.2 | -4.2 |
| **Q04609** | 4NGS *Glutamate carboxypeptidase II* (**GCPII**) | J34 | -6.2 | -6.2 |
| **Q05193** | 2X2E *Dynamin-1* (**DNM1**) | GDP | -6.0 | -6.0 |
| **P78540** | 4IXU *Arginase-2, mitochondrial* (**ARG2**) | 38I | -5.7 | -5.7 |
| **P14618** | 1T5A *Pyruvate Kinase M2* (**PKM2**) | FBP | -6.0 | -6.0 |
| **P28845** | 3PDJ *Corticosteroid 11-beta-dehydrogenase isoenzyme 1* (**HSD11B1**) | NAP | -4.9 | -5.0 |
| **P28845** | 3PDJ *Corticosteroid 11-beta-dehydrogenase isoenzyme 1* (**HSD11B1**) | 3PJ | -4.9 | -4.5 |
| **P34998** | 3EHU *Corticotropin-releasing factor receptor 1* (**CRHR1**) | MAL | -4.9 | -4.9 |
| **P34998** | 3EHU *Corticotropin-releasing factor receptor 1* (**CRHR1**) | BTB | -4.9 | -4.5 |
| **P49862** | 2QXG *Kallikrein 7* (**KLK7**) | K7I | -4.0 | -4.0 |
| **P27487** | 1WCY *Adenosine deaminase complexing protein 2* (**ADCP-2**) | 4 | -5.6 | -5.6 |
| **Q92830** | 5H86 *Histone acetyltransferase GCN5* (**KAT2A**) | BCO | -5.0 | -5.0 |
| **Q92830** | 3D7C *Histone acetyltransferase GCN5* (**KAT2A**) | 0 | -4.9 | -4.9 |
| **O75496** | 2WVR *Geminin* (**GMNN**) | 0 | -3.5 | -3.5 |
| **O75164** | 5FY8.A *Lysine-specific demethylase 4A* (**KDM4A**) | N81 | -5.1 | -5.1 |
| **O95050** | 2A14.A *Indolethylamine N-methyltransferase* (**INMT**) | SAH | -5.3 | -5.3 |
| **Q9UNA4** | 1T3N.B *DNA polymerase iota* (**POLI**) | TTP | -5.2 | -5.2 |
| **P51449** | 5AYG.B *Nuclear receptor ROR-gamma* (**RORC**) | 4LQ | -5.0 | -5.0 |
| **P00915** | 1BZM.A *Carbonic anhydrase I* (**CA1**) | MZM | -4.6 | -4.6 |
| **P04150** | 1M2Z.A *Glucocorticoid Receptor* (**GCR**, **NR3C1**) | BOG | -3.5 | -3.5 |
| **P03956** | 1SU3.B *Matrix metalloproteinase-1* (**MMP1**) | EPE | -4.3 | -4.3 |
| **P08254** | 1B3D.B *Stromelysin-1/Matrix metalloproteinase-3* (**MMP3**) | S27 | -5.6 | -5.5 |
| **P84022** | 1U7F *Mothers against decapentaplegic homolog 3* (**SMAD3**) | 0 | -4.6 | -4.6 |
| **P84022** | 1OZJ.A *Mothers against decapentaplegic homolog 3* (**SMAD3**) | 1 | -4.3 | -4.3 |
| **Q99720** | 5HK1.C *Sigma non-opioid intracellular receptor 1* (**OPRS1**, **SIGMAR1**) | 61W | -5.1 | -5.1 |
| **P63092** | HM *Guanine nucleotide-binding protein G(s) subunit alpha* (**GNAS**) | 0 | -4.9 | -5.2 |
| **P83916** | 2FMM.A *Chromobox protein homolog 1* (**CBX1**) | 0 | -4.0 | -4.0 |
| **P83916** | 3F2U.A *Chromobox protein homolog 1* (**CBX1**) | 0 | -3.2 | -3.1 |
| **Q9H3R0** | 5KR7.A *Lysine-specific demethylase 4C* (**KDM4C**) | 6X9 | -5.1 | -5.1 |
| **P39748** | 1UL1.Z *Flap endonuclease 1* (**FEN1**) | 0 | -4.7 | -4.6 |
| **P10145** | 1IL8.A *Interleukin 8* (**IL-8**, **CXCL8**) | 1 | -3.8 | -3.8 |
| **P08253** | 1GXD.A *Matrix metalloproteinase-2* (**MMP2**) | 3 | -5.9 | -5.9 |
| **Q9NUW8** | 1QZQ.A *Tyrosyl DNA phosphodiesterase 1* (**TDP1**) | 0 | -4.7 | -4.7 |

Each target entry contains its UniprotID, the PDBID (with the sole exception of P63092 in which the homology model developed is designed as HM), the ligand (as indicator of the crystallographic cavity) or cavity number (when blind docking calculations were performed) against which the docking calculations were realized and the binding energies of the docking calculations. Each calculation was performed twice (R0, R1) to avoid false positives.

*If the PDB ID is followed by a dot and a capital letter, it indicates the chain of the structure where the crystallographic cavity used is located. If this information is not shown, it is because there is only one chain within the PDB file.

*If more than one PDB ID within a UNIPROT entry is shown, it indicates that different regions of the amino acid sequence were covered

±The more negative the binding energy, the more plausible the interaction.

**Table S2. MM/GBSA-based binding energy rescoring calculations over MD simulations**

| **Uniprot ID** | **PDB ID*** | **Ligand/Cavity number** | **MM/GBSA Energy (kcal/mol) ^±^** |
| --- | --- | --- | --- |
| **Q9Y3Q0** | 3FED *Glutamate carboxypeptidase III* (**NAALAD2, GCPIII**) | BIX | -53.8000 |
| **Q9UNA4** | 1T3N.B *DNA polymerase iota* (**POLI**) | TTP | -42.6918 |
| **P27708** | 4C6E *CAD protein* (**CAD**) | NCD | -42.6691 |
| **P51449** | 5AYG.B *Nuclear receptor ROR-gamma* (**RORC**) | 4LQ | -41.1213 |
| **O95050** | 2A14.A *Indolethylamine N-methyltransferase* (**INMT**) | SAH | -37.7325 |
| **Q05193** | 2X2E *Dynamin-1* (**DNM1**) | GDP | -36.3854 |
| **P00915** | 1BZM.A *Carbonic anhydrase I* (**CA1**) | MZM | -36.3236 |
| **P08253** | 1GXD.A *Matrix metalloproteinase-2* (**MMP2**) | 3 | -35.9904 |
| **Q9NUW8** | 1QZQ.A Tyrosyl DNA phosphodiesterase (**TDP1**) | 0 | -34.8231 |
| **P14618** | 1T5A *Pyruvate Kinase M2* (**PKM2**) | FBP | -31.2832 |
| **Q04609** | 4NGS *Glutamate carboxypeptidase II* (**GCPII**) | J34 | -29.5409 |
| **P39748** | 1UL1.Z *Flap endonuclease 1* (**FEN1**) | 0 | -29.4901 |
| **P28845** | 3PDJ *Corticosteroid 11-beta-dehydrogenase isoenzyme 1* (**HSD11B1**) | NAP | -29.0065 |
| **P34998** | 3EHU *Corticotropin-releasing factor receptor 1* (**CRHR1**) | BTB | -28.3458 |
| **P34998** | 3EHU *Corticotropin-releasing factor receptor 1* (**CRHR1**) | MAL | -28.1045 |
| **Q9H3R0** | 5KR7.A *Lysine-specific demethylase 4C* (**KDM4C**) | 6X9 | -27.2795 |
| **P28845** | 3PDJ *Corticosteroid 11-beta-dehydrogenase isoenzyme 1* (**HSD11B1**) | 3PJ | -25.6190 |
| **Q92830** | 3D7C *Histone acetyltransferase GCN5* (**KAT2A**) | 0 | -25.5712 |
| **O75164** | 5FY8.A *Lysine-specific demethylase 4A* (**KDM4A**) | N81 | -25.5269 |
| **Q99720** | 5HK1.C *Sigma non-opioid intracellular receptor 1* (**OPRS1**, **SIGMAR1**) | 61W | -25.2010 |
| **P49862** | 2QXG *Kallikrein 7* (**KLK7**) | K7I | -22.9065 |
| **P84022** | 2FMM.A *Mothers against decapentaplegic homolog 3* (**SMAD3**) | 1 | -15.5008 |
| **P08254** | 1B3D.B *Stromelysin-1/Matrix metalloproteinase-3* (**MMP3**) | S27 | -21.4462 |
| **Q92830** | 5H86 *Histone acetyltransferase GCN5* (**KAT2A**) | BCO | -21.2802 |
| **P83916** | 2FMM.A *Chromobox protein homolog 1* (**CBX1**) | 0 | -21.2802 |
| **Q16769** | 3PBB *Glutaminyl-peptide cyclotransferase* (**QPCT**) | PBD | -21,0137 |
| **P03956** | 1SU3.B *Matrix metalloproteinase-1* (**MMP1**) | EPE | -18.8926 |
| **P10145** | 1IL8.A *Interleukin 8* (**IL-8**, **CXCL8**) | 1 | -18.0282 |
| **P63092** | HM *Guanine nucleotide-binding protein G(s) subunit alpha* (**GNAS)** | 0FI | -17.0555 |
| **P04150** | 1M2Z.A *Glucocorticoid Receptor* (**GCR**, **NR3C1**) | BOG | -16.6065 |
| **P84022** | 1U7F.A *Mothers against decapentaplegic homolog 3* (**SMAD3**) | 0 | -15.5008 |
| **P42262** | 2XH *Glutamate receptor 2* (**GRM2**) | 7T9 | -14.7116 |
| **P00491** | 3PHB *Purine nucleoside phosphorylase* (**PNP**) | IM5 | -14.5027 |
| **P05089** | 3GN0 *Arginase-1* (**ARG1**) | DMO | -13.5001 |
| **P27487** | 1WCY  *Adenosine deaminase complexing protein 2* (**ADCP-2**) | 4 | -11.2007 |
| **P83916** | 3F2U.A *Chromobox protein homolog 1* (**CBX1**) | 0 | -9.1778 |
| **P78540** | 4IXU *Arginase-2, mitochondrial* (**ARG2**) | 38I | -6.9485 |
| **O75496** | 2WVR *Geminin* (**GMNN**) | 0 | -6.4592 |

*If the PDB ID is followed by a dot and a capital letter, it indicates the chain of the structure where the crystallographic cavity used is located.

If this information is not shown, it is because there is only one chain within the PDB file.

±The more negative the binding energy, the more plausible the interaction.

**Table S3. Docking binding energies and MM/GBSA-based energy rescoring calculations of metformin against selected histone demethylases (KDMs)**

|  |  |  | **Docking** | **MM/GBSA** |
| --- | --- | --- | --- | --- |
|  |  |  | **Binding Energy** | **Binding Energy** |
| **Uniprot ID** | **Protein name** | **PDBID** | **(kcal/mol) R0/R1** | **(kcal/mol) ^±^** |
| **O15550** | **KDM6A/UTX** | 3AVS | -5.2/-5.3 | -26.1494 |
| **O15054** | **KDM6B/JMJD3** | 2XUE | -5.1/-5.1 | -22.9444 |
| **O75164** | **KDM4A** | 5FY8 | -5.1/-5.1 | -25.5269 |
| **O94953** | **KDM4B** | 4LXL | -5.7/-5.7 | -10.4384 |
| **Q9H3R0** | **KDM4C** | 5KR7 | -5.1/-5.1 | -27.2795 |
| **Q6B0I6** | **KDM4D** | 4D6Q | -5.0/-4.9 | -26.8297 |
| **B2RXH2** | **KDM4E** | 2W2I | -5.1/-5.1 | -14.4764 |
| **O60341** | **LSD1** | 3ZN0 | -5.2/-5.3 | -37.6179 |

±The more negative the binding energy, the more plausible the interaction.

**Table S4. WEB-based Gene SeT Analysis Toolkit evaluation of metformin target-associated morbidities**

| **DataSets^a^** | **GSEA** | **ORA** |
| --- | --- | --- |
| **VS targets** | - Mammary neoplasms | - Leishmaniasis, Cutaneous - Cocaine-Related Disorders - Neoplasm Invasiveness - Oral Submucous Fibrosis - Liver Cirrhosis, Alcoholic - Myopathy - Cerebral Hemorrhage - Pulmonary Hypertension - Hyperactive behavior - Hypertensive disease |
| **Random #1** | - | - Ataxia, Spinocerebellar - Non-alcoholic Fatty Liver Disease - Ulcerative Colitis - Crohn Disease - Peripheral Neuropathy - Mammary Neoplasms - IGA Glomerulonephritis |
| **Random #2** | - | - Electrocardiogram: P-R interval - Immunologic Deficiency Syndromes - Fanconi Anemia - Testicular Germ Cell Tumor - IGA Glomerulonephritis - Cardiomyopathies - Polycystic Ovary Syndrome - Peripheral Neuropathy |
| **Random #3** | - | - Ventricular tachycardia, catecholaminergic polymorphic, 1 (disorder) - Hanot's cirrhosis - Tachycardia, Ventricular - Diabetic Cardiomyopathies - Long QT Syndrome - Schizophrenia, Childhood - Tachycardia - Uterine Cervical Neoplasm - Primary biliary cirrhosis - Colitis |
| **Random #4** | - | - Ear, patella, short stature syndrome - Bone neoplasms - Diabetic Retinopathy - Dermatitis, Atopic - Cocaine-Related Disorders - Degenerative polyarthritis - Seizures - Juvenile arthritis - Schizophrenia - IGA Glomerulonephritis |
| **Random #5** | - | - Cryptorchidism - Erectile dysfunction - Renal Insufficiency - Diabetic Neuropathies - Parkinson disease, late-onset - Lung Injury - Leber Congenital Amaurosis - Head and Neck Neoplasms - Neurotoxicity Syndromes - Cerebral Hemorrhage |

**^a^Datasets (Uniprot IDs)**

| **VS targets** | **Random #1** | **Random #2** | **Random #3** | **Random #4** | **Random #5** |
| --- | --- | --- | --- | --- | --- |
| P27487 | Q6SWD5 | 05166 | Q8NGE8 | Q6FZM0 | P0DJU3 |
| O15244 | Q13263 | P0CE40 | Q8N112 | Q5PD60 | Q90X44 |
| Q92830 | Q5GS59 | Q3I5I7 | Q62048 | Q96A61 | Q9H920 |
| O75496 | Q5XXA9 | Q9HAP6 | Q1DUU8 | Q01813 | Q62186 |
| O15245 | P56484 | Q18BI4 | Q1E3S4 | A6LIG0 | Q5PL41 |
| Q9Y6L6 | A3R4N4 | Q8BVE8 | P67252 | Q1J9Z9 | P21917 |
| P02545 | Q7Z3Y8 | Q9DSQ0 | O14894 | Q08C75 | P40286 |
| O94956 | O95810 | B9E9E3 | P55065 | Q2GFR2 | Q9UN88 |
| Q96FL8 | A1AJF5 | Q8BM96 | Q800C4 | P53675 | Q2GGZ1 |
| Q9NPD5 | P82650 | O60318 | Q400C7 | O00469 | Q5PDC3 |
| O75164 | P09251 | Q9QUG2 | Q18CI7 | Q1JHJ1 | Q8N5U1 |
| O95050 | Q86306 | Q9ZMM1 | Q6A814 | Q2GI32 | A6QYC8 |
| Q9UNA4 | P11823 | Q5PLX6 | Q9Y6X2 | Q2YHJ5 | Q86UW2 |
| P51449 | Q9Y512 | O15533 | P59509 | Q8N6S4 | Q07817 |
| P00915 | Q5SPH9 | Q9GLX9 | O43916 | Q2GKG2 | Q6XTL0 |
| P04150 | Q1JP34 | Q5PFL9 | A1YG01 | F5HCJ2 | Q83FZ3 |
| P03956 | Q96IQ7 | Q9Y6I3 | Q13477 | Q6ZQ08 | A6NJ78 |
| P08254 | Q5PFG2 | A0A0B5ADV0 | Q03252 | Q9WKC4 | Q9HC97 |
| P84022 | Q96I25 | P50826 | Q1J449 | Q59L12 | Q9ZM57 |
| Q99720 | Q8VHK9 | P0CJ77 | Q9UKN7 | Q5GS62 | P68340 |
| Q7Z2H8 | O43927 | F1QCC6 | P68874 | A1XBS5 | P0C793 |
| P63092 | Q8IZP0 | P35263 | A4GDR3 | P82944 | P0CM87 |
| P83916 | Q55P57 | Q1JLD9 | Q5PE68 | F5HA10 | Q8G756 |
| Q9H3R0 | F5HA10 | Q8G5R2 | A7H5V3 | P43361 | P21103 |
| P39748 | Q0BUJ8 | Q96KN4 | C4ZAY9 | O43929 | Q4L6H9 |
| P10145 | P58170 | Q9HCE0 | A0A0C4DH72 | Q9Z282 | Q1J8N2 |
| P08253 | D2Q9C9 | Q9BUG6 | A7GZP8 | J7H670 | Q6PEY1 |
| Q9NUW8 | Q8NHB8 | Q8VC49 | P0DMN0 | Q5MNV8 | P04593 |
| Q9Y3Q0 | Q86UF2 | C0ME07 | P97751 | A5UNC8 | Q182F4 |
| Q16769 | Q8ND76 | Q5VTT5 | Q92736 | Q02261 | P52452 |
| P00491 | P0CO35 | Q9NVI1 | O42222 | Q05329 | P16721 |
| P05089 | Q658T7 | A6NGN9 | Q9H6R0 | Q8WVE7 | Q0BSK3 |
| P27708 | Q4L6G1 | Q9H706 | Q6SW62 | Q92764 | A4GE42 |
| P42262 | P0DKA1 | Q02920 | Q5GS22 | Q9ZN52 | P08473 |
| Q04609 | Q9Y3A6 | Q7Z4L0 | Q8WUA7 | A6NHT5 | Q9BSY4 |
| Q05193 | P0CH87 | Q8DXC1 | B7XHR6 | B2RXH8 | Q7Z3Z2 |
| P78540 | Q9TU27 | Q9ULM0 | P56747 | Q5PH01 | Q8N5U6 |
| P14618 | Q64518 | Q18CT5 | P04492 | Q6G0N8 | Q9H9F9 |
| P28845 | Q5AL36 | C4K014 | Q16633 | O14753 | P58062 |
| P34998 | E9PQX1 | Q8TAD4 | P0C7T3 | E9QHE3 | B1NRQ8 |
| P49862 | Q1JDL6 | P10266 | O02773 | Q6J868 | P50151 |

**Table S5. Disease-related and Gene Ontology (GO) analyses of the target list for metformin**

| UniprotID | | DisGeNET | Gene Ontology | |
| --- | --- | --- | --- | --- |
| Q9Y3Q0 | | - Tobacco Use - Disorder - Schizophrenia | **GO - Molecular function**   1. [carboxypeptidase activity](http://www.ebi.ac.uk/QuickGO/GTerm?id=GO:0004180) 2. [dipeptidase activity](http://www.ebi.ac.uk/QuickGO/GTerm?id=GO:0016805) 3. [dipeptidyl-peptidase activity](http://www.ebi.ac.uk/QuickGO/GTerm?id=GO:0008239) 4. [metal ion binding](http://www.ebi.ac.uk/QuickGO/GTerm?id=GO:0046872) 5. [metallopeptidase activity](http://www.ebi.ac.uk/QuickGO/GTerm?id=GO:0008237) 6. [N-formylglutamate deformylase activity](http://www.ebi.ac.uk/QuickGO/GTerm?id=GO:0050129) 7. [serine-type peptidase activity](http://www.ebi.ac.uk/QuickGO/GTerm?id=GO:0008236)   **GO - Biological process**   1. [cellular amino acid biosynthetic process](http://www.ebi.ac.uk/QuickGO/GTerm?id=GO:0008652) 2. [neurotransmitter catabolic process](http://www.ebi.ac.uk/QuickGO/GTerm?id=GO:0042135) 3. [proteolysis](http://www.ebi.ac.uk/QuickGO/GTerm?id=GO:0006508) | |
| Q16769 | | - Melanoma - Schizophrenia - Chronic Lymphocytic Leukemia - Dermatitis - Allergic Contact - Osteoporosis - Germ cell tumor - granulosa cell tumor | **GO - Molecular function**   1. [glutaminyl-peptide cyclotransferase activity](http://www.ebi.ac.uk/QuickGO/GTerm?id=GO:0016603) 2. [zinc ion binding](http://www.ebi.ac.uk/QuickGO/GTerm?id=GO:0008270)   **GO - Biological process**   1. [cellular protein modification process](http://www.ebi.ac.uk/QuickGO/GTerm?id=GO:0006464) 2. [peptidyl-pyroglutamic acid biosynthetic process, using glutaminyl-peptide cyclotransferase](http://www.ebi.ac.uk/QuickGO/GTerm?id=GO:0017186) | |
| P00491 | | - Purine-nucleoside phosphorylase deficiency - Osteoporosis - Arsenic Poisoning - Drug-Induced Liver Injury - Alzheimer's Disease - Neurodegenerative Disorders - Malignant neoplasm of prostate - Prostatecarcinoma - Severe Combined Immunodeficiency - Immunologic Deficiency Syndromes | **GO - Molecular function**   1. [drug binding](http://www.ebi.ac.uk/QuickGO/GTerm?id=GO:0008144) 2. [nucleoside binding](http://www.ebi.ac.uk/QuickGO/GTerm?id=GO:0001882) 3. [phosphate ion binding](http://www.ebi.ac.uk/QuickGO/GTerm?id=GO:0042301) 4. [purine nucleobase binding](http://www.ebi.ac.uk/QuickGO/GTerm?id=GO:0002060) 5. [purine-nucleoside phosphorylase activity](http://www.ebi.ac.uk/QuickGO/GTerm?id=GO:0004731)   **GO - Biological process**   1. [immune response](http://www.ebi.ac.uk/QuickGO/GTerm?id=GO:0006955) 2. [inosine catabolic process](http://www.ebi.ac.uk/QuickGO/GTerm?id=GO:0006148) 3. [interleukin-2 secretion](http://www.ebi.ac.uk/QuickGO/GTerm?id=GO:0070970) 4. [NAD biosynthesis via nicotinamide riboside salvage pathway](http://www.ebi.ac.uk/QuickGO/GTerm?id=GO:0034356) 5. [nicotinamide riboside catabolic process](http://www.ebi.ac.uk/QuickGO/GTerm?id=GO:0006738) 6. [nucleobase-containing compound metabolic process](http://www.ebi.ac.uk/QuickGO/GTerm?id=GO:0006139) 7. [positive regulation of alpha-beta T cell differentiation](http://www.ebi.ac.uk/QuickGO/GTerm?id=GO:0046638) 8. [positive regulation of T cell proliferation](http://www.ebi.ac.uk/QuickGO/GTerm?id=GO:0042102) 9. [purine-containing compound salvage](http://www.ebi.ac.uk/QuickGO/GTerm?id=GO:0043101) 10. [purine nucleotide catabolic process](http://www.ebi.ac.uk/QuickGO/GTerm?id=GO:0006195) 11. [response to drug](http://www.ebi.ac.uk/QuickGO/GTerm?id=GO:0042493) 12. [urate biosynthetic process](http://www.ebi.ac.uk/QuickGO/GTerm?id=GO:0034418) | |
| P05089 | | - Hyperargininemia - Silicosis - Asthma - Precancerous Conditions - Amino Acid Metabolism - Inborn Errors - Leishmaniasis - Hair Diseases - Myopathy - Immune System Diseases - Drug-Induced Liver Injury | **GO - Molecular function**   1. [arginase activity](http://www.ebi.ac.uk/QuickGO/GTerm?id=GO:0004053) 2. [manganese ion binding](http://www.ebi.ac.uk/QuickGO/GTerm?id=GO:0030145)   **GO - Biological process**   1. [aging](http://www.ebi.ac.uk/QuickGO/GTerm?id=GO:0007568) 2. [arginine catabolic process](http://www.ebi.ac.uk/QuickGO/GTerm?id=GO:0006527) 3. [arginine catabolic process to ornithine](http://www.ebi.ac.uk/QuickGO/GTerm?id=GO:0019547) 4. [cellular response to dexamethasone stimulus](http://www.ebi.ac.uk/QuickGO/GTerm?id=GO:0071549) 5. [cellular response to glucagon stimulus](http://www.ebi.ac.uk/QuickGO/GTerm?id=GO:0071377) 6. [cellular response to hydrogen peroxide](http://www.ebi.ac.uk/QuickGO/GTerm?id=GO:0070301) 7. [cellular response to interleukin-4](http://www.ebi.ac.uk/QuickGO/GTerm?id=GO:0071353) 8. [cellular response to lipopolysaccharide](http://www.ebi.ac.uk/QuickGO/GTerm?id=GO:0071222) 9. [cellular response to transforming growth factor beta stimulus](http://www.ebi.ac.uk/QuickGO/GTerm?id=GO:0071560) 10. [collagen biosynthetic process](http://www.ebi.ac.uk/QuickGO/GTerm?id=GO:0032964) 11. [liver development](http://www.ebi.ac.uk/QuickGO/GTerm?id=GO:0001889) 12. [lung development](http://www.ebi.ac.uk/QuickGO/GTerm?id=GO:0030324) 13. [mammary gland involution](http://www.ebi.ac.uk/QuickGO/GTerm?id=GO:0060056) 14. [maternal process involved in female pregnancy](http://www.ebi.ac.uk/QuickGO/GTerm?id=GO:0060135) 15. [positive regulation of endothelial cell proliferation](http://www.ebi.ac.uk/QuickGO/GTerm?id=GO:0001938) 16. [protein homotrimerization](http://www.ebi.ac.uk/QuickGO/GTerm?id=GO:0070207) 17. [regulation of L-arginine import](http://www.ebi.ac.uk/QuickGO/GTerm?id=GO:0010963) 18. [response to amine](http://www.ebi.ac.uk/QuickGO/GTerm?id=GO:0014075) 19. [response to amino acid](http://www.ebi.ac.uk/QuickGO/GTerm?id=GO:0043200) 20. [response to axon injury](http://www.ebi.ac.uk/QuickGO/GTerm?id=GO:0048678) 21. [response to cadmium ion](http://www.ebi.ac.uk/QuickGO/GTerm?id=GO:0046686) 22. [response to drug](http://www.ebi.ac.uk/QuickGO/GTerm?id=GO:0042493) 23. [response to herbicide](http://www.ebi.ac.uk/QuickGO/GTerm?id=GO:0009635) 24. [response to manganese ion](http://www.ebi.ac.uk/QuickGO/GTerm?id=GO:0010042) 25. [response to methylmercury](http://www.ebi.ac.uk/QuickGO/GTerm?id=GO:0051597) 26. [response to selenium ion](http://www.ebi.ac.uk/QuickGO/GTerm?id=GO:0010269) 27. [response to vitamin A](http://www.ebi.ac.uk/QuickGO/GTerm?id=GO:0033189) 28. [response to vitamin E](http://www.ebi.ac.uk/QuickGO/GTerm?id=GO:0033197) 29. [response to zinc ion](http://www.ebi.ac.uk/QuickGO/GTerm?id=GO:0010043) 30. [urea cycle](http://www.ebi.ac.uk/QuickGO/GTerm?id=GO:0000050) | |
| P27708 | | - Anemia - Sickle Cell - Congenital hypoplastic anemia - Acute Erythroblastic Leukemia - Hereditary spherocytosis - Beta Thalassemia - Liver carcinoma - Liver Neoplasms - Fetal Growth Retardation - Diabetes Mellitus - Mammary Neoplasms | **GO - Molecular function**   1. [aspartate binding](http://www.ebi.ac.uk/QuickGO/GTerm?id=GO:0070335) 2. [aspartate carbamoyltransferase activity](http://www.ebi.ac.uk/QuickGO/GTerm?id=GO:0004070) 3. [ATP binding](http://www.ebi.ac.uk/QuickGO/GTerm?id=GO:0005524) 4. [carbamoyl-phosphate synthase (ammonia) activity](http://www.ebi.ac.uk/QuickGO/GTerm?id=GO:0004087) 5. [carbamoyl-phosphate synthase (glutamine-hydrolyzing) activity](http://www.ebi.ac.uk/QuickGO/GTerm?id=GO:0004088) 6. [dihydroorotase activity](http://www.ebi.ac.uk/QuickGO/GTerm?id=GO:0004151) 7. [enzyme binding](http://www.ebi.ac.uk/QuickGO/GTerm?id=GO:0019899) 8. [identical protein binding](http://www.ebi.ac.uk/QuickGO/GTerm?id=GO:0042802) 9. [protein kinase activity](http://www.ebi.ac.uk/QuickGO/GTerm?id=GO:0004672) 10. [zinc ion binding](http://www.ebi.ac.uk/QuickGO/GTerm?id=GO:0008270)   **GO - Biological process**   1. ['de novo' pyrimidine nucleobase biosynthetic process](http://www.ebi.ac.uk/QuickGO/GTerm?id=GO:0006207) 2. ['de novo' UMP biosynthetic process](http://www.ebi.ac.uk/QuickGO/GTerm?id=GO:0044205) 3. [arginine biosynthetic process](http://www.ebi.ac.uk/QuickGO/GTerm?id=GO:0006526) 4. [cellular response to drug](http://www.ebi.ac.uk/QuickGO/GTerm?id=GO:0035690) 5. [cellular response to epidermal growth factor stimulus](http://www.ebi.ac.uk/QuickGO/GTerm?id=GO:0071364) 6. [drug metabolic process](http://www.ebi.ac.uk/QuickGO/GTerm?id=GO:0017144) 7. [female pregnancy](http://www.ebi.ac.uk/QuickGO/GTerm?id=GO:0007565) 8. [glutamine metabolic process](http://www.ebi.ac.uk/QuickGO/GTerm?id=GO:0006541) 9. [heart development](http://www.ebi.ac.uk/QuickGO/GTerm?id=GO:0007507) 10. [lactation](http://www.ebi.ac.uk/QuickGO/GTerm?id=GO:0007595) 11. [liver development](http://www.ebi.ac.uk/QuickGO/GTerm?id=GO:0001889) 12. [organ regeneration](http://www.ebi.ac.uk/QuickGO/GTerm?id=GO:0031100) 13. [peptidyl-threonine phosphorylation](http://www.ebi.ac.uk/QuickGO/GTerm?id=GO:0018107) 14. [protein autophosphorylation](http://www.ebi.ac.uk/QuickGO/GTerm?id=GO:0046777) 15. [pyrimidine nucleoside biosynthetic process](http://www.ebi.ac.uk/QuickGO/GTerm?id=GO:0046134) 16. [response to amine](http://www.ebi.ac.uk/QuickGO/GTerm?id=GO:0014075) 17. [response to caffeine](http://www.ebi.ac.uk/QuickGO/GTerm?id=GO:0031000) 18. [response to cortisol](http://www.ebi.ac.uk/QuickGO/GTerm?id=GO:0051414) 19. [response to testosterone](http://www.ebi.ac.uk/QuickGO/GTerm?id=GO:0033574) 20. [urea cycle](http://www.ebi.ac.uk/QuickGO/GTerm?id=GO:0000050) 21. [UTP biosynthetic process](http://www.ebi.ac.uk/QuickGO/GTerm?id=GO:0006228) | |
| P42262 | | - Status Epilepticus - Hyperactive behavior - Cocaine-Related Disorders - Substance Withdrawal Syndrome - Schizophrenia - Amyotrophic Lateral Sclerosis - Epilepsy - Temporal Lobe - Alzheimer's Disease - Bipolar Disorder - Weight Gain Adverse Event | **GO - Molecular function**   1. [AMPA glutamate receptor activity](http://www.ebi.ac.uk/QuickGO/GTerm?id=GO:0004971) 2. [excitatory extracellular ligand-gated ion channel activity](http://www.ebi.ac.uk/QuickGO/GTerm?id=GO:0005231) 3. [extracellular-glutamate-gated ion channel activity](http://www.ebi.ac.uk/QuickGO/GTerm?id=GO:0005234) 4. [ionotropic glutamate receptor activity](http://www.ebi.ac.uk/QuickGO/GTerm?id=GO:0004970)   **GO - Biological process**   1. [ionotropic glutamate receptor signaling pathway](http://www.ebi.ac.uk/QuickGO/GTerm?id=GO:0035235) 2. [signal transduction](http://www.ebi.ac.uk/QuickGO/GTerm?id=GO:0007165) 3. [synaptic transmission](http://www.ebi.ac.uk/QuickGO/GTerm?id=GO:0007268) | |
| Q04609 | | - Prostatic Neoplasms - Malignant neoplasm of prostate - Prostate carcinoma - Colorectal Cancer - Hyperhomocysteinemia - Kidney Failure - Cardiovascular Diseases - Neural Tube Defects - Coronary heart disease - Adenocarcinoma | **GO - Molecular function**   1. [Ac-Asp-Glu binding](http://www.ebi.ac.uk/QuickGO/GTerm?id=GO:1904492) 2. [dipeptidase activity](http://www.ebi.ac.uk/QuickGO/GTerm?id=GO:0016805) 3. [metal ion binding](http://www.ebi.ac.uk/QuickGO/GTerm?id=GO:0046872) 4. [metallocarboxypeptidase activity](http://www.ebi.ac.uk/QuickGO/GTerm?id=GO:0004181) 5. [peptidase activity](http://www.ebi.ac.uk/QuickGO/GTerm?id=GO:0008233) 6. [tetrahydrofolyl-poly(glutamate) polymer binding](http://www.ebi.ac.uk/QuickGO/GTerm?id=GO:1904493)   **GO - Biological process**   1. [cellular amino acid biosynthetic process](http://www.ebi.ac.uk/QuickGO/GTerm?id=GO:0008652) 2. [C-terminal protein deglutamylation](http://www.ebi.ac.uk/QuickGO/GTerm?id=GO:0035609) 3. [folic acid-containing compound metabolic process](http://www.ebi.ac.uk/QuickGO/GTerm?id=GO:0006760) 4. [proteolysis](http://www.ebi.ac.uk/QuickGO/GTerm?id=GO:0006508) | |
| Q05193 | | - Early infantile epileptic encephalopathy with suppression bursts - Lennox-Gastaut syndrome - Idiopathic generalized epilepsy - Schizophrenia - Cytomegalovirus Infections - Attention deficit hyperactivity disorder - Tobacco Use Disorder | **GO - Molecular function**   1. [GTPase activity](http://www.ebi.ac.uk/QuickGO/GTerm?id=GO:0003924) 2. [GTP binding](http://www.ebi.ac.uk/QuickGO/GTerm?id=GO:0005525) 3. [identical protein binding](http://www.ebi.ac.uk/QuickGO/GTerm?id=GO:0042802) 4. [poly(A) RNA binding](http://www.ebi.ac.uk/QuickGO/GTerm?id=GO:0044822) 5. [protein kinase binding](http://www.ebi.ac.uk/QuickGO/GTerm?id=GO:0019901)   **GO - Biological process**   1. [adult locomotory behavior](http://www.ebi.ac.uk/QuickGO/GTerm?id=GO:0008344) 2. [clathrin-mediated endocytosis](http://www.ebi.ac.uk/QuickGO/GTerm?id=GO:0072583) 3. [endocytosis](http://www.ebi.ac.uk/QuickGO/GTerm?id=GO:0006897) 4. [endosome organization](http://www.ebi.ac.uk/QuickGO/GTerm?id=GO:0007032) 5. [ephrin receptor signaling pathway](http://www.ebi.ac.uk/QuickGO/GTerm?id=GO:0048013) 6. [G-protein coupled receptor internalization](http://www.ebi.ac.uk/QuickGO/GTerm?id=GO:0002031) 7. [positive regulation of synaptic vesicle recycling](http://www.ebi.ac.uk/QuickGO/GTerm?id=GO:1903423) 8. [protein tetramerization](http://www.ebi.ac.uk/QuickGO/GTerm?id=GO:0051262) 9. [receptor-mediated endocytosis](http://www.ebi.ac.uk/QuickGO/GTerm?id=GO:0006898) 10. [sensory perception of sound](http://www.ebi.ac.uk/QuickGO/GTerm?id=GO:0007605) 11. [synaptic transmission, GABAergic](http://www.ebi.ac.uk/QuickGO/GTerm?id=GO:0051932) 12. [toxin transport](http://www.ebi.ac.uk/QuickGO/GTerm?id=GO:1901998) | |
| P78540 | | - Asthma - Prostatic Neoplasms - Carcinoma - Pancreatic Ductal - Disease Progression - Necrosis - Pulmonary Hypertension - Hypertensivedisease - Hyperoxia - Ureteral obstruction - Sepsis | **GO - Molecular function**   1. [arginase activity](http://www.ebi.ac.uk/QuickGO/GTerm?id=GO:0004053) 2. [metal ion binding](http://www.ebi.ac.uk/QuickGO/GTerm?id=GO:0046872)   **GO - Biological process**   1. [arginine metabolic process](http://www.ebi.ac.uk/QuickGO/GTerm?id=GO:0006525) 2. [nitric oxide biosynthetic process](http://www.ebi.ac.uk/QuickGO/GTerm?id=GO:0006809) 3. [striated muscle contraction](http://www.ebi.ac.uk/QuickGO/GTerm?id=GO:0006941) 4. [urea cycle](http://www.ebi.ac.uk/QuickGO/GTerm?id=GO:0000050) 5. [ureteric bud development](http://www.ebi.ac.uk/QuickGO/GTerm?id=GO:0001657) | |
| P14618 | | - Liver carcinoma - Osteoporosis - Neoplasm Invasiveness - Mammary Neoplasms - Animal Mammary Neoplasms - Colorectal Neoplasms - Carcinogenesis - Mammary Neoplasms - Lung Neoplasms - Granulosa cell tumor | **GO - Molecular function**   1. [ADP binding](http://www.ebi.ac.uk/QuickGO/GTerm?id=GO:0043531) 2. [ATP binding](http://www.ebi.ac.uk/QuickGO/GTerm?id=GO:0005524) 3. [kinase activity](http://www.ebi.ac.uk/QuickGO/GTerm?id=GO:0016301) 4. [magnesium ion binding](http://www.ebi.ac.uk/QuickGO/GTerm?id=GO:0000287) 5. [MHC class II protein complex binding](http://www.ebi.ac.uk/QuickGO/GTerm?id=GO:0023026) 6. [poly(A) RNA binding](http://www.ebi.ac.uk/QuickGO/GTerm?id=GO:0044822) 7. [potassium ion binding](http://www.ebi.ac.uk/QuickGO/GTerm?id=GO:0030955) 8. [pyruvate kinase activity](http://www.ebi.ac.uk/QuickGO/GTerm?id=GO:0004743)   **GO - Biological process**   1. [ATP biosynthetic process](http://www.ebi.ac.uk/QuickGO/GTerm?id=GO:0006754) 2. [canonical glycolysis](http://www.ebi.ac.uk/QuickGO/GTerm?id=GO:0061621) 3. [liver development](http://www.ebi.ac.uk/QuickGO/GTerm?id=GO:0001889) 4. [organ regeneration](http://www.ebi.ac.uk/QuickGO/GTerm?id=GO:0031100) 5. [programmed cell death](http://www.ebi.ac.uk/QuickGO/GTerm?id=GO:0012501) 6. [pyruvate biosynthetic process](http://www.ebi.ac.uk/QuickGO/GTerm?id=GO:0042866) 7. [response to gravity](http://www.ebi.ac.uk/QuickGO/GTerm?id=GO:0009629) 8. [response to hypoxia](http://www.ebi.ac.uk/QuickGO/GTerm?id=GO:0001666) 9. [response to insulin](http://www.ebi.ac.uk/QuickGO/GTerm?id=GO:0032868) 10. [response to muscle inactivity](http://www.ebi.ac.uk/QuickGO/GTerm?id=GO:0014870) 11. [response to nutrient](http://www.ebi.ac.uk/QuickGO/GTerm?id=GO:0007584) 12. [skeletal muscle tissue regeneration](http://www.ebi.ac.uk/QuickGO/GTerm?id=GO:0043403) | |
| P28845 | | - Obesity - cortisone reductase deficiency 2 - hypertensive disease - Cortisone reductase deficiency - Obesity - Abdominal - Rheumatoid Arthritis - Hyperglycemia - Diabetes Mellitus - Dermatitis - Allergic Contact - Insulin Resistance | **GO - Molecular function**   1. [11-beta-hydroxysteroid dehydrogenase (NADP+) activity](http://www.ebi.ac.uk/QuickGO/GTerm?id=GO:0070524) -EC 2. [11-beta-hydroxysteroid dehydrogenase [NAD(P)] activity](http://www.ebi.ac.uk/QuickGO/GTerm?id=GO:0003845)   **GO - Biological process**   1. [glucocorticoid biosynthetic process](http://www.ebi.ac.uk/QuickGO/GTerm?id=GO:0006704) 2. [lung development](http://www.ebi.ac.uk/QuickGO/GTerm?id=GO:0030324) | |
| P34998 | | - Cocaine-Related Disorders - Major Depressive Disorder - Anxiety Disorders - Parkinson Disease - Contact Dermatitis - Anorexia - Hyperactive behavior - Depressive disorder - Hypertensive disease - Irritable Bowel Syndrome | **GO - Molecular function**   1. [corticotrophin-releasing factor receptor activity](http://www.ebi.ac.uk/QuickGO/GTerm?id=GO:0015056)   **GO - Biological process**   1. [activation of adenylate cyclase activity](http://www.ebi.ac.uk/QuickGO/GTerm?id=GO:0007190) 2. [cell surface receptor signaling pathway](http://www.ebi.ac.uk/QuickGO/GTerm?id=GO:0007166) 3. [cellular response to corticotropin-releasing hormone stimulus](http://www.ebi.ac.uk/QuickGO/GTerm?id=GO:0071376) 4. [corticotropin secretion](http://www.ebi.ac.uk/QuickGO/GTerm?id=GO:0051458) 5. [female pregnancy](http://www.ebi.ac.uk/QuickGO/GTerm?id=GO:0007565) 6. [immune response](http://www.ebi.ac.uk/QuickGO/GTerm?id=GO:0006955) 7. [negative regulation of voltage-gated calcium channel activity](http://www.ebi.ac.uk/QuickGO/GTerm?id=GO:1901386) 8. [parturition](http://www.ebi.ac.uk/QuickGO/GTerm?id=GO:0007567) 9. [positive regulation of adenylate cyclase activity involved in G-protein coupled receptor signaling pathway](http://www.ebi.ac.uk/QuickGO/GTerm?id=GO:0010579) 10. [regulation of adenylate cyclase activity involved in G-protein coupled receptor signaling pathway](http://www.ebi.ac.uk/QuickGO/GTerm?id=GO:0010578) 11. [regulation of corticosterone secretion](http://www.ebi.ac.uk/QuickGO/GTerm?id=GO:2000852) | |
| P49862 | | - Dermatitis - Atopic - Eczema - Ovarian neoplasm - Pancreatic Neoplasm - Dementia - Malignant neoplasm of prostate - Dermatitis and eczema - Malignant neoplasm of ovary - Ovarian Carcinoma - Carcinogenesis | **GO - Molecular function**   1. [metalloendopeptidase activity](http://www.ebi.ac.uk/QuickGO/GTerm?id=GO:0004222) 2. [peptidase activity](http://www.ebi.ac.uk/QuickGO/GTerm?id=GO:0008233) 3. [serine-type endopeptidase activity](http://www.ebi.ac.uk/QuickGO/GTerm?id=GO:0004252) 4. [serine-type peptidase activity](http://www.ebi.ac.uk/QuickGO/GTerm?id=GO:0008236)   **GO - Biological process**   1. [epidermis development](http://www.ebi.ac.uk/QuickGO/GTerm?id=GO:0008544) 2. [extracellular matrix disassembly](http://www.ebi.ac.uk/QuickGO/GTerm?id=GO:0022617) 3. [positive regulation of antibacterial peptide production](http://www.ebi.ac.uk/QuickGO/GTerm?id=GO:0002803) | |
| P27487 | | - Rheumatoid Arthritis - Proteinuria - Prostatic Neoplasms - Adenocarcinoma - Diabetes Mellitus - Non-Insulin-Dependent - Severe Combined Immunodeficiency - Glioma - Metabolic Syndrome X - Hepatitis C Chronic - Adult T-Cell Lymphoma/Leukemia | **GO - Molecular function**   1. [dipeptidyl-peptidase activity](http://www.ebi.ac.uk/QuickGO/GTerm?id=GO:0008239) 2. [identical protein binding](http://www.ebi.ac.uk/QuickGO/GTerm?id=GO:0042802) 3. [protease binding](http://www.ebi.ac.uk/QuickGO/GTerm?id=GO:0002020) 4. [protein homodimerization activity](http://www.ebi.ac.uk/QuickGO/GTerm?id=GO:0042803) 5. [receptor binding](http://www.ebi.ac.uk/QuickGO/GTerm?id=GO:0005102) 6. [serine-type endopeptidase activity](http://www.ebi.ac.uk/QuickGO/GTerm?id=GO:0004252) 7. [serine-type peptidase activity](http://www.ebi.ac.uk/QuickGO/GTerm?id=GO:0008236) 8. [virus receptor activity](http://www.ebi.ac.uk/QuickGO/GTerm?id=GO:0001618)   **GO - Biological process**   1. [behavioral fear response](http://www.ebi.ac.uk/QuickGO/GTerm?id=GO:0001662) 2. [endothelial cell migration](http://www.ebi.ac.uk/QuickGO/GTerm?id=GO:0043542) 3. [locomotory exploration behavior](http://www.ebi.ac.uk/QuickGO/GTerm?id=GO:0035641) 4. [negative regulation of extracellular matrix disassembly](http://www.ebi.ac.uk/QuickGO/GTerm?id=GO:0010716) 5. [positive regulation of cell proliferation](http://www.ebi.ac.uk/QuickGO/GTerm?id=GO:0008284) 6. [psychomotor behavior](http://www.ebi.ac.uk/QuickGO/GTerm?id=GO:0036343) 7. [regulation of cell-cell adhesion mediated by integrin](http://www.ebi.ac.uk/QuickGO/GTerm?id=GO:0033632) 8. [response to hypoxia](http://www.ebi.ac.uk/QuickGO/GTerm?id=GO:0001666) 9. [T cell activation](http://www.ebi.ac.uk/QuickGO/GTerm?id=GO:0042110) 10. [T cell costimulation](http://www.ebi.ac.uk/QuickGO/GTerm?id=GO:0031295) | |
| O15244 | | - Kidney Diseases - Chronic Kidney Diseases - Finding of creatinine level - Hyperuricemia - Kidney Failure Chronic - Renal Insufficiency - Cholestasis - Extrahepatic - Endotoxemia - Acute kidney injury - Intrahepatic Cholestasis | **GO - Molecular function**   1. [choline transmembrane transporter activity](http://www.ebi.ac.uk/QuickGO/GTerm?id=GO:0015220) 2. [dopamine transmembrane transporter activity](http://www.ebi.ac.uk/QuickGO/GTerm?id=GO:0005329) 3. [neurotransmitter transporter activity](http://www.ebi.ac.uk/QuickGO/GTerm?id=GO:0005326) 4. [organic cation transmembrane transporter activity](http://www.ebi.ac.uk/QuickGO/GTerm?id=GO:0015101) 5. [quaternary ammonium group transmembrane transporter activity](http://www.ebi.ac.uk/QuickGO/GTerm?id=GO:0015651) 6. [steroid binding](http://www.ebi.ac.uk/QuickGO/GTerm?id=GO:0005496)   **GO - Biological process**   1. [body fluid secretion](http://www.ebi.ac.uk/QuickGO/GTerm?id=GO:0007589) 2. [choline transport](http://www.ebi.ac.uk/QuickGO/GTerm?id=GO:0015871) 3. [dopamine transport](http://www.ebi.ac.uk/QuickGO/GTerm?id=GO:0015872) 4. [drug transmembrane transport](http://www.ebi.ac.uk/QuickGO/GTerm?id=GO:0006855) 5. [histamine transport](http://www.ebi.ac.uk/QuickGO/GTerm?id=GO:0051608) 6. [neurotransmitter biosynthetic process](http://www.ebi.ac.uk/QuickGO/GTerm?id=GO:0042136) 7. [neurotransmitter secretion](http://www.ebi.ac.uk/QuickGO/GTerm?id=GO:0007269) 8. [organic cation transport](http://www.ebi.ac.uk/QuickGO/GTerm?id=GO:0015695) | |
| Q92830 | | - Spinocerebellar Ataxia Type 7 - Malignant neoplasm of breast - Breast Carcinoma - Dosage-sensitive sex reversal - Cancer Cell Growth - Colon Carcinoma - Neurodegenerative Disorders - Pre B-cell acute lymphoblastic leukemia - Adenocarcinoma of colon - gastric cancer | **GO - Molecular function**   1. [chromatin binding](http://www.ebi.ac.uk/QuickGO/GTerm?id=GO:0003682) 2. [H3 histone acetyltransferase activity](http://www.ebi.ac.uk/QuickGO/GTerm?id=GO:0010484) 3. [histone acetyltransferase activity](http://www.ebi.ac.uk/QuickGO/GTerm?id=GO:0004402) 4. [histone acetyltransferase activity (H4-K12 specific)](http://www.ebi.ac.uk/QuickGO/GTerm?id=GO:0043997) 5. [histone deacetylase binding](http://www.ebi.ac.uk/QuickGO/GTerm?id=GO:0042826) 6. [transcription coactivator activity](http://www.ebi.ac.uk/QuickGO/GTerm?id=GO:0003713) 7. [transcription factor binding](http://www.ebi.ac.uk/QuickGO/GTerm?id=GO:0008134)   **GO - Biological process**   1. [alpha-tubulin acetylation](http://www.ebi.ac.uk/QuickGO/GTerm?id=GO:0071929) 2. [cardiac muscle cell differentiation](http://www.ebi.ac.uk/QuickGO/GTerm?id=GO:0055007) 3. [cell proliferation](http://www.ebi.ac.uk/QuickGO/GTerm?id=GO:0008283) 4. [cellular response to nerve growth factor stimulus](http://www.ebi.ac.uk/QuickGO/GTerm?id=GO:1990090) 5. [cellular response to tumor necrosis factor](http://www.ebi.ac.uk/QuickGO/GTerm?id=GO:0071356) 6. [chromatin remodeling](http://www.ebi.ac.uk/QuickGO/GTerm?id=GO:0006338) 7. [histone deubiquitination](http://www.ebi.ac.uk/QuickGO/GTerm?id=GO:0016578) 8. [histone H3 acetylation](http://www.ebi.ac.uk/QuickGO/GTerm?id=GO:0043966) 9. [histone H3-K14 acetylation](http://www.ebi.ac.uk/QuickGO/GTerm?id=GO:0044154) 10. [intracellular distribution of mitochondria](http://www.ebi.ac.uk/QuickGO/GTerm?id=GO:0048312) 11. [in utero embryonic development](http://www.ebi.ac.uk/QuickGO/GTerm?id=GO:0001701) 12. [metencephalon development](http://www.ebi.ac.uk/QuickGO/GTerm?id=GO:0022037) 13. [midbrain development](http://www.ebi.ac.uk/QuickGO/GTerm?id=GO:0030901) 14. [multicellular organism growth](http://www.ebi.ac.uk/QuickGO/GTerm?id=GO:0035264) 15. [neural tube closure](http://www.ebi.ac.uk/QuickGO/GTerm?id=GO:0001843) 16. [positive regulation of cell projection organization](http://www.ebi.ac.uk/QuickGO/GTerm?id=GO:0031346) 17. [positive regulation of gene expression, epigenetic](http://www.ebi.ac.uk/QuickGO/GTerm?id=GO:0045815) 18. [positive regulation of gluconeogenesis by positive regulation of transcription from RNA polymerase II promoter](http://www.ebi.ac.uk/QuickGO/GTerm?id=GO:0035948) 19. [positive regulation of histone acetylation](http://www.ebi.ac.uk/QuickGO/GTerm?id=GO:0035066) 20. [positive regulation of protein targeting to mitochondrion](http://www.ebi.ac.uk/QuickGO/GTerm?id=GO:1903955) 21. [positive regulation of transcription regulatory region DNA binding](http://www.ebi.ac.uk/QuickGO/GTerm?id=GO:2000679) 22. [regulation of mitophagy](http://www.ebi.ac.uk/QuickGO/GTerm?id=GO:1903146) 23. [regulation of protein stability](http://www.ebi.ac.uk/QuickGO/GTerm?id=GO:0031647) 24. [regulation of transcription from RNA polymerase II promoter](http://www.ebi.ac.uk/QuickGO/GTerm?id=GO:0006357) 25. [response to nutrient levels](http://www.ebi.ac.uk/QuickGO/GTerm?id=GO:0031667) 26. [response to organic cyclic compound](http://www.ebi.ac.uk/QuickGO/GTerm?id=GO:0014070) 27. [somitogenesis](http://www.ebi.ac.uk/QuickGO/GTerm?id=GO:0001756) 28. [telencephalon development](http://www.ebi.ac.uk/QuickGO/GTerm?id=GO:0021537) 29. [transcription from RNA polymerase II promoter](http://www.ebi.ac.uk/QuickGO/GTerm?id=GO:0006366) 30. [viral process](http://www.ebi.ac.uk/QuickGO/GTerm?id=GO:0016032) | |
| O75496 | | - Short stature syndrome - Liver carcinoma - Pancreatic Neoplasm - Fibrosis - Chronic Lymphocytic Leukemia - Malignant neoplasm of breast - Breast Carcinoma - Neoplasm Metastasis - Multiple Myeloma - Pancreatic carcinoma | **GO - Molecular function**   1. [histone deacetylase binding](http://www.ebi.ac.uk/QuickGO/GTerm?id=GO:0042826) 2. [transcription corepressor activity](http://www.ebi.ac.uk/QuickGO/GTerm?id=GO:0003714)   **GO - Biological process**   1. [cell cycle](http://www.ebi.ac.uk/QuickGO/GTerm?id=GO:0007049) 2. [negative regulation of cell cycle](http://www.ebi.ac.uk/QuickGO/GTerm?id=GO:0045786) 3. [negative regulation of DNA replication](http://www.ebi.ac.uk/QuickGO/GTerm?id=GO:0008156) 4. [negative regulation of transcription, DNA-templated](http://www.ebi.ac.uk/QuickGO/GTerm?id=GO:0045892) 5. [organ morphogenesis](http://www.ebi.ac.uk/QuickGO/GTerm?id=GO:0009887) 6. [protein complex assembly](http://www.ebi.ac.uk/QuickGO/GTerm?id=GO:0006461) | |
| O15245 | | - Obesity - Colonic Neoplasms - Diabetes Mellitus - Acute kidney injury - Asthma - Cholestasis - Extrahepatic - Intrahepatic Cholestasis - Endotoxemia - Myeloid Leukemia - Diabetes Mellitus Non-Insulin-Dependent | **GO - Molecular function**   1. [acetylcholine transmembrane transporter activity](http://www.ebi.ac.uk/QuickGO/GTerm?id=GO:0005277) 2. [dopamine transmembrane transporter activity](http://www.ebi.ac.uk/QuickGO/GTerm?id=GO:0005329) 3. [norepinephrine transmembrane transporter activity](http://www.ebi.ac.uk/QuickGO/GTerm?id=GO:0005333) 4. [organic cation transmembrane transporter activity](http://www.ebi.ac.uk/QuickGO/GTerm?id=GO:0015101) 5. [quaternary ammonium group transmembrane transporter activity](http://www.ebi.ac.uk/QuickGO/GTerm?id=GO:0015651) 6. [secondary active organic cation transmembrane transporter activity](http://www.ebi.ac.uk/QuickGO/GTerm?id=GO:0008513)   **GO - Biological process**   1. [dopamine transport](http://www.ebi.ac.uk/QuickGO/GTerm?id=GO:0015872) 2. [drug transmembrane transport](http://www.ebi.ac.uk/QuickGO/GTerm?id=GO:0006855) 3. [epinephrine transport](http://www.ebi.ac.uk/QuickGO/GTerm?id=GO:0048241) 4. [establishment or maintenance of transmembrane electrochemical gradient](http://www.ebi.ac.uk/QuickGO/GTerm?id=GO:0010248) 5. [neurotransmitter transport](http://www.ebi.ac.uk/QuickGO/GTerm?id=GO:0006836) 6. [norepinephrine transport](http://www.ebi.ac.uk/QuickGO/GTerm?id=GO:0015874) 7. [organic cation transport](http://www.ebi.ac.uk/QuickGO/GTerm?id=GO:0015695) 8. [protein homooligomerization](http://www.ebi.ac.uk/QuickGO/GTerm?id=GO:0051260) | |
| Q9Y6L6 | | - Rotor Syndrome - Myopathy - Adverse reaction to drug - Chagas - Cardiomyopathy - Mammary Neoplasms - Hyperbilirubinemia - Hyperbilirubinemia - Neonatal - Myocardial Infarction - Hyperlipidemia - Cholelithiasis | **GO - Molecular function**   1. [bile acid transmembrane transporter activity](http://www.ebi.ac.uk/QuickGO/GTerm?id=GO:0015125) 2. [sodium-independent organic anion transmembrane transporter activity](http://www.ebi.ac.uk/QuickGO/GTerm?id=GO:0015347) 3. [thyroid hormone transmembrane transporter activity](http://www.ebi.ac.uk/QuickGO/GTerm?id=GO:0015349)   **GO - Biological process**   1. [bile acid and bile salt transport](http://www.ebi.ac.uk/QuickGO/GTerm?id=GO:0015721) 2. [bile acid metabolic process](http://www.ebi.ac.uk/QuickGO/GTerm?id=GO:0008206) 3. [organic anion transport](http://www.ebi.ac.uk/QuickGO/GTerm?id=GO:0015711) 4. [sodium-independent organic anion transport](http://www.ebi.ac.uk/QuickGO/GTerm?id=GO:0043252) | |
| P02545 | | - Progeria - Muscular dystrophy - Congenital lmna-related (disorder) - Charcot-Marie-Tooth disease - Mandibuloacral dysostosis - Muscular dystrophy   limb-girdle   - Autosomal Dominant Emery-Dreifuss Muscular Dystrophy (disorder) - Cardiomyopathy - Familial Idiopathic - Familial Partial Lipodystrophy - Lethal tight skin contracture syndrome (disorder) - Heart-hand syndrome   Slovenian type | **GO - Molecular function**   1. [structural molecule activity](http://www.ebi.ac.uk/QuickGO/GTerm?id=GO:0005198)   **GO - Biological process**   1. [cellular response to hypoxia](http://www.ebi.ac.uk/QuickGO/GTerm?id=GO:0071456) 2. [establishment or maintenance of microtubule cytoskeleton polarity](http://www.ebi.ac.uk/QuickGO/GTerm?id=GO:0030951) 3. [IRE1-mediated unfolded protein response](http://www.ebi.ac.uk/QuickGO/GTerm?id=GO:0036498) 4. [mitotic nuclear envelope disassembly](http://www.ebi.ac.uk/QuickGO/GTerm?id=GO:0007077) 5. [mitotic nuclear envelope reassembly](http://www.ebi.ac.uk/QuickGO/GTerm?id=GO:0007084) 6. [muscle organ development](http://www.ebi.ac.uk/QuickGO/GTerm?id=GO:0007517) 7. [negative regulation of adipose tissue development](http://www.ebi.ac.uk/QuickGO/GTerm?id=GO:1904178) 8. [negative regulation of extrinsic apoptotic signaling pathway](http://www.ebi.ac.uk/QuickGO/GTerm?id=GO:2001237) 9. [negative regulation of release of cytochrome c from mitochondria](http://www.ebi.ac.uk/QuickGO/GTerm?id=GO:0090201) 10. [positive regulation of cell aging](http://www.ebi.ac.uk/QuickGO/GTerm?id=GO:0090343) 11. [positive regulation of osteoblast differentiation](http://www.ebi.ac.uk/QuickGO/GTerm?id=GO:0045669) 12. [protein localization to nucleus](http://www.ebi.ac.uk/QuickGO/GTerm?id=GO:0034504) 13. [regulation of cell migration](http://www.ebi.ac.uk/QuickGO/GTerm?id=GO:0030334) 14. [regulation of protein localization to nucleus](http://www.ebi.ac.uk/QuickGO/GTerm?id=GO:1900180) 15. [response to mechanical stimulus](http://www.ebi.ac.uk/QuickGO/GTerm?id=GO:0009612) 16. [spermatogenesis](http://www.ebi.ac.uk/QuickGO/GTerm?id=GO:0007283) 17. [sterol regulatory element binding protein import into nucleus](http://www.ebi.ac.uk/QuickGO/GTerm?id=GO:0035105) 18. [ventricular cardiac muscle cell development](http://www.ebi.ac.uk/QuickGO/GTerm?id=GO:0055015) | |
| O94956 | | - Hyperbilirubinemia - Malignant neoplasm of breast - Breast Carcinoma - Prostate carcinoma - Malignant neoplasm of prostate - Carcinogenesis - Thyroid carcinoma - Malignant neoplasm of pancreas - Malignant neoplasm of liver - Liver and Intrahepatic Biliary Tract Carcinoma | **GO - Molecular function**   1. [bile acid transmembrane transporter activity](http://www.ebi.ac.uk/QuickGO/GTerm?id=GO:0015125) 2. [organic anion transmembrane transporter activity](http://www.ebi.ac.uk/QuickGO/GTerm?id=GO:0008514) 3. [sodium-independent organic anion transmembrane transporter activity](http://www.ebi.ac.uk/QuickGO/GTerm?id=GO:0015347)   **GO - Biological process**   1. [bile acid and bile salt transport](http://www.ebi.ac.uk/QuickGO/GTerm?id=GO:0015721) 2. [sodium-independent organic anion transport](http://www.ebi.ac.uk/QuickGO/GTerm?id=GO:0043252) | |
| Q96FL8 | | - Kidney Diseases - Diabetes Mellitus - Diabetes Mellitus Non-Insulin-Dependent - Diabetes - Acidosis | **GO - Molecular function**   1. [drug:proton antiporter activity](http://www.ebi.ac.uk/QuickGO/GTerm?id=GO:0015307) 2. [drug transmembrane transporter activity](http://www.ebi.ac.uk/QuickGO/GTerm?id=GO:0015238) 3. [monovalent cation:proton antiporter activity](http://www.ebi.ac.uk/QuickGO/GTerm?id=GO:0005451)   **GO - Biological process**   1. [drug transmembrane transport](http://www.ebi.ac.uk/QuickGO/GTerm?id=GO:0006855) 2. [organic cation transport](http://www.ebi.ac.uk/QuickGO/GTerm?id=GO:0015695) 3. [transmembrane transport](http://www.ebi.ac.uk/QuickGO/GTerm?id=GO:0055085) | |
| Q9NPD5 | | - Rotor Syndrome - Colonic Neoplasms - Lung Neoplasms - Hyperbilirubinemia - Neoplasm Metastasis - Cholestasis - Reperfusion Injury - Sepsis - Hepatitis - Animal Neutropenia | **GO - Molecular function**   1. [bile acid transmembrane transporter activity](http://www.ebi.ac.uk/QuickGO/GTerm?id=GO:0015125) 2. [organic anion transmembrane transporter activity](http://www.ebi.ac.uk/QuickGO/GTerm?id=GO:0008514) 3. [sodium-independent organic anion transmembrane transporter activity](http://www.ebi.ac.uk/QuickGO/GTerm?id=GO:0015347)   **GO - Biological process**   1. [bile acid and bile salt transport](http://www.ebi.ac.uk/QuickGO/GTerm?id=GO:0015721) 2. [bile acid metabolic process](http://www.ebi.ac.uk/QuickGO/GTerm?id=GO:0008206) 3. [organic anion transport](http://www.ebi.ac.uk/QuickGO/GTerm?id=GO:0015711) 4. [sodium-independent organic anion transport](http://www.ebi.ac.uk/QuickGO/GTerm?id=GO:0043252) | |
| [O75164](http://www.uniprot.org/uniprot/O75164) | - Hypertensive disease - Malignant neoplasm of breast - Breast Carcinoma - Prostate carcinoma - Carcinogenesis - Malignant neoplasm of prostate - Mammary Neoplasms - Neoplasm Metastasis - Herpesviridae Infections - Squamous cell carcinoma of esophagus | | | **GO - Molecular function**   1. [histone demethylase activity](http://www.ebi.ac.uk/QuickGO/GTerm?id=GO:0032452) 2. [histone demethylase activity (H3-K36 specific)](http://www.ebi.ac.uk/QuickGO/GTerm?id=GO:0051864) 3. [methylated histone binding](http://www.ebi.ac.uk/QuickGO/GTerm?id=GO:0035064) 4. [ubiquitin protein ligase binding](http://www.ebi.ac.uk/QuickGO/GTerm?id=GO:0031625) 5. [zinc ion binding](http://www.ebi.ac.uk/QuickGO/GTerm?id=GO:0008270)   **GO - Biological process**   1. [cardiac muscle hypertrophy in response to stress](http://www.ebi.ac.uk/QuickGO/GTerm?id=GO:0014898) 2. [histone demethylation](http://www.ebi.ac.uk/QuickGO/GTerm?id=GO:0016577) 3. [negative regulation of autophagy](http://www.ebi.ac.uk/QuickGO/GTerm?id=GO:0010507) 4. [negative regulation of gene expression](http://www.ebi.ac.uk/QuickGO/GTerm?id=GO:0010629) 5. [negative regulation of histone H3-K9 trimethylation](http://www.ebi.ac.uk/QuickGO/GTerm?id=GO:1900113) 6. [negative regulation of transcription, DNA-templated](http://www.ebi.ac.uk/QuickGO/GTerm?id=GO:0045892) 7. [transcription, DNA-templated](http://www.ebi.ac.uk/QuickGO/GTerm?id=GO:0006351) 8. [viral process](http://www.ebi.ac.uk/QuickGO/GTerm?id=GO:0016032) |
| O95050 |  | | | **GO - Molecular function**   1. [amine N-methyltransferase activity](http://www.ebi.ac.uk/QuickGO/GTerm?id=GO:0030748) 2. [thioether S-methyltransferase activity](http://www.ebi.ac.uk/QuickGO/GTerm?id=GO:0004790) -EC   **GO - Biological process**   1. [amine metabolic process](http://www.ebi.ac.uk/QuickGO/GTerm?id=GO:0009308) 2. [methylation](http://www.ebi.ac.uk/QuickGO/GTerm?id=GO:0032259) 3. [response to toxic substance](http://www.ebi.ac.uk/QuickGO/GTerm?id=GO:0009636) |
| Q9UNA4 | - Inflammatory dermatosis - Malignant neoplasm of lung - Squamous cell carcinoma - Mammary Neoplasms - Burkitt Lymphoma - Malignant neoplasm of breast - Malignant neoplasm of urinary bladder - Hematologic Neoplasms - Malignant neoplasm of prostate - Asthma | | | **GO - Molecular function**   1. [damaged DNA binding](http://www.ebi.ac.uk/QuickGO/GTerm?id=GO:0003684) 2. [DNA-directed DNA polymerase activity](http://www.ebi.ac.uk/QuickGO/GTerm?id=GO:0003887) 3. [metal ion binding](http://www.ebi.ac.uk/QuickGO/GTerm?id=GO:0046872)   **GO - Biological process**   1. [DNA repair](http://www.ebi.ac.uk/QuickGO/GTerm?id=GO:0006281) 2. [DNA replication](http://www.ebi.ac.uk/QuickGO/GTerm?id=GO:0006260) 3. [error-prone translesion synthesis](http://www.ebi.ac.uk/QuickGO/GTerm?id=GO:0042276) 4. [translesion synthesis](http://www.ebi.ac.uk/QuickGO/GTerm?id=GO:0019985) |
| P51449 | - Inflammatory Bowel Diseases - Depressive Symptoms - Inflammation - Lymphedema - Tobacco Use Disorder - Obesity - Necrotizing Enterocolitis - Malignant neoplasm of breast - Autoimmune Diseases - Breast Carcinoma | | | **GO - Molecular function**   1. [DNA binding](http://www.ebi.ac.uk/QuickGO/GTerm?id=GO:0003677) 2. [oxysterol binding](http://www.ebi.ac.uk/QuickGO/GTerm?id=GO:0008142) 3. [RNA polymerase II transcription factor activity, ligand-activated sequence-specific DNA binding](http://www.ebi.ac.uk/QuickGO/GTerm?id=GO:0004879) 4. [sequence-specific DNA binding](http://www.ebi.ac.uk/QuickGO/GTerm?id=GO:0043565) 5. [steroid hormone receptor activity](http://www.ebi.ac.uk/QuickGO/GTerm?id=GO:0003707) 6. [transcription factor activity, direct ligand regulated sequence-specific DNA binding](http://www.ebi.ac.uk/QuickGO/GTerm?id=GO:0098531) 7. [transcription factor activity, sequence-specific DNA binding](http://www.ebi.ac.uk/QuickGO/GTerm?id=GO:0003700) 8. [zinc ion binding](http://www.ebi.ac.uk/QuickGO/GTerm?id=GO:0008270)   **GO - Biological process**   1. [adipose tissue development](http://www.ebi.ac.uk/QuickGO/GTerm?id=GO:0060612) 2. [cellular response to sterol](http://www.ebi.ac.uk/QuickGO/GTerm?id=GO:0036315) 3. [circadian regulation of gene expression](http://www.ebi.ac.uk/QuickGO/GTerm?id=GO:0032922) 4. [lymph node development](http://www.ebi.ac.uk/QuickGO/GTerm?id=GO:0048535) 5. [negative regulation of thymocyte apoptotic process](http://www.ebi.ac.uk/QuickGO/GTerm?id=GO:0070244) 6. [Peyer's patch development](http://www.ebi.ac.uk/QuickGO/GTerm?id=GO:0048541) 7. [positive regulation of circadian rhythm](http://www.ebi.ac.uk/QuickGO/GTerm?id=GO:0042753) 8. [positive regulation of transcription, DNA-templated](http://www.ebi.ac.uk/QuickGO/GTerm?id=GO:0045893) 9. [regulation of fat cell differentiation](http://www.ebi.ac.uk/QuickGO/GTerm?id=GO:0045598) 10. [regulation of glucose metabolic process](http://www.ebi.ac.uk/QuickGO/GTerm?id=GO:0010906) 11. [regulation of steroid metabolic process](http://www.ebi.ac.uk/QuickGO/GTerm?id=GO:0019218) 12. [regulation of transcription involved in cell fate commitment](http://www.ebi.ac.uk/QuickGO/GTerm?id=GO:0060850) 13. [T-helper 17 cell differentiation](http://www.ebi.ac.uk/QuickGO/GTerm?id=GO:0072539) 14. [T-helper cell differentiation](http://www.ebi.ac.uk/QuickGO/GTerm?id=GO:0042093) 15. [transcription initiation from RNA polymerase II promoter](http://www.ebi.ac.uk/QuickGO/GTerm?id=GO:0006367) 16. [xenobiotic metabolic process](http://www.ebi.ac.uk/QuickGO/GTerm?id=GO:0006805) |
| P00915 | - Adenocarcinoma - Squamous cell carcinoma of esophagus - Stomach Neoplasms - Pneumoconiosis - Kidney Diseases - Ulcerative Colitis - Colon Carcinoma - Peroxisomal Disorders - Rheumatoid Arthritis - Obstructive chronic bronchitis with acute exacerbation | | | **GO - Molecular function**   1. [arylesterase activity](http://www.ebi.ac.uk/QuickGO/GTerm?id=GO:0004064) 2. [carbonate dehydratase activity](http://www.ebi.ac.uk/QuickGO/GTerm?id=GO:0004089) 3. [hydro-lyase activity](http://www.ebi.ac.uk/QuickGO/GTerm?id=GO:0016836) 4. [zinc ion binding](http://www.ebi.ac.uk/QuickGO/GTerm?id=GO:0008270)   **GO - Biological process**   1. [bicarbonate transport](http://www.ebi.ac.uk/QuickGO/GTerm?id=GO:0015701) 2. [one-carbon metabolic process](http://www.ebi.ac.uk/QuickGO/GTerm?id=GO:0006730) |
| P04150 | - Glucocorticoid Receptor Deficiency - Hypertensive disease - Depressive disorder - Bipolar Disorder - Schizophrenia - Ocular Hypertension - Psychotic Disorders - Endometriosis - Prostatic Neoplasms - Lung Diseases - Obstructive | | | **GO - Molecular function**   1. [glucocorticoid-activated RNA polymerase II transcription factor binding transcription factor activity](http://www.ebi.ac.uk/QuickGO/GTerm?id=GO:0038051) 2. [glucocorticoid receptor activity](http://www.ebi.ac.uk/QuickGO/GTerm?id=GO:0004883) 3. [RNA binding](http://www.ebi.ac.uk/QuickGO/GTerm?id=GO:0003723) 4. [RNA polymerase II core promoter proximal region sequence-specific DNA binding](http://www.ebi.ac.uk/QuickGO/GTerm?id=GO:0000978) 5. [steroid binding](http://www.ebi.ac.uk/QuickGO/GTerm?id=GO:0005496) 6. [steroid hormone binding](http://www.ebi.ac.uk/QuickGO/GTerm?id=GO:1990239) 7. [transcriptional activator activity, RNA polymerase II core promoter proximal region sequence-specific binding](http://www.ebi.ac.uk/QuickGO/GTerm?id=GO:0001077) 8. [transcription factor activity, sequence-specific DNA binding](http://www.ebi.ac.uk/QuickGO/GTerm?id=GO:0003700) 9. [zinc ion binding](http://www.ebi.ac.uk/QuickGO/GTerm?id=GO:0008270)   **GO - Biological process**   1. [apoptotic process](http://www.ebi.ac.uk/QuickGO/GTerm?id=GO:0006915) 2. [cell division](http://www.ebi.ac.uk/QuickGO/GTerm?id=GO:0051301) 3. [cellular response to steroid hormone stimulus](http://www.ebi.ac.uk/QuickGO/GTerm?id=GO:0071383) 4. [chromatin modification](http://www.ebi.ac.uk/QuickGO/GTerm?id=GO:0016568) 5. [chromosome segregation](http://www.ebi.ac.uk/QuickGO/GTerm?id=GO:0007059) 6. [mitotic nuclear division](http://www.ebi.ac.uk/QuickGO/GTerm?id=GO:0007067) 7. [positive regulation of transcription from RNA polymerase II promoter](http://www.ebi.ac.uk/QuickGO/GTerm?id=GO:0045944) 8. [regulation of transcription, DNA-templated](http://www.ebi.ac.uk/QuickGO/GTerm?id=GO:0006355) 9. [signal transduction](http://www.ebi.ac.uk/QuickGO/GTerm?id=GO:0007165) 10. [transcription, DNA-templated](http://www.ebi.ac.uk/QuickGO/GTerm?id=GO:0006351) 11. [transcription from RNA polymerase II promoter](http://www.ebi.ac.uk/QuickGO/GTerm?id=GO:0006366) 12. [transcription initiation from RNA polymerase II promoter](http://www.ebi.ac.uk/QuickGO/GTerm?id=GO:0006367) |
| P03956 | - Chronic Obstructive Airway Disease - Neoplasm Metastasis - Colorectal Neoplasms - Mammary Neoplasms - Lung Neoplasms - Atherosclerosis - Oral Submucous Fibrosis - Neoplasm Invasiveness - Non-Small Cell Lung Carcinoma - Hallopeau-Siemens Disease | | | **GO - Molecular function**   1. [calcium ion binding](http://www.ebi.ac.uk/QuickGO/GTerm?id=GO:0005509) 2. [endopeptidase activity](http://www.ebi.ac.uk/QuickGO/GTerm?id=GO:0004175) 3. [metalloendopeptidase activity](http://www.ebi.ac.uk/QuickGO/GTerm?id=GO:0004222) 4. [serine-type endopeptidase activity](http://www.ebi.ac.uk/QuickGO/GTerm?id=GO:0004252) 5. [zinc ion binding](http://www.ebi.ac.uk/QuickGO/GTerm?id=GO:0008270)   **GO - Biological process**   1. [cellular protein metabolic process](http://www.ebi.ac.uk/QuickGO/GTerm?id=GO:0044267) 2. [collagen catabolic process](http://www.ebi.ac.uk/QuickGO/GTerm?id=GO:0030574) 3. [extracellular matrix disassembly](http://www.ebi.ac.uk/QuickGO/GTerm?id=GO:0022617) 4. [leukocyte migration](http://www.ebi.ac.uk/QuickGO/GTerm?id=GO:0050900) 5. [positive regulation of protein oligomerization](http://www.ebi.ac.uk/QuickGO/GTerm?id=GO:0032461) 6. [proteolysis](http://www.ebi.ac.uk/QuickGO/GTerm?id=GO:0006508) 7. [viral process](http://www.ebi.ac.uk/QuickGO/GTerm?id=GO:0016032) |
| P08254 | - Hyperalgesia - Arthritis - Coronary Artery Disease - Mammary Neoplasms - Astrocytoma - Coronary Restenosis - Cerebral Hemorrhage - Coronary heart disease - Neoplasm Invasiveness - Peripheral Neuropathy | | | **GO - Molecular function**   1. [calcium ion binding](http://www.ebi.ac.uk/QuickGO/GTerm?id=GO:0005509) 2. [endopeptidase activity](http://www.ebi.ac.uk/QuickGO/GTerm?id=GO:0004175) 3. [metalloendopeptidase activity](http://www.ebi.ac.uk/QuickGO/GTerm?id=GO:0004222) 4. [serine-type endopeptidase activity](http://www.ebi.ac.uk/QuickGO/GTerm?id=GO:0004252) 5. [zinc ion binding](http://www.ebi.ac.uk/QuickGO/GTerm?id=GO:0008270)   **GO - Biological process**   1. [cellular response to nitric oxide](http://www.ebi.ac.uk/QuickGO/GTerm?id=GO:0071732) 2. [collagen catabolic process](http://www.ebi.ac.uk/QuickGO/GTerm?id=GO:0030574) 3. [extracellular matrix disassembly](http://www.ebi.ac.uk/QuickGO/GTerm?id=GO:0022617) 4. [negative regulation of hydrogen peroxide metabolic process](http://www.ebi.ac.uk/QuickGO/GTerm?id=GO:0010727) 5. [positive regulation of oxidative stress-induced cell death](http://www.ebi.ac.uk/QuickGO/GTerm?id=GO:1903209) 6. [positive regulation of protein oligomerization](http://www.ebi.ac.uk/QuickGO/GTerm?id=GO:0032461) 7. [proteolysis](http://www.ebi.ac.uk/QuickGO/GTerm?id=GO:0006508) |
| P84022 | - Loeys-Dietz Syndrome 3 - Crohn Disease - Loeys-Dietz Syndrome - Degenerative polyarthritis - Asthma - Coronary heart disease - Liver Cirrhosis - Fibroid Tumor - Aortic Aneurysm - Inflammatory Bowel Diseases | | | **GO - Molecular function**   1. [bHLH transcription factor binding](http://www.ebi.ac.uk/QuickGO/GTerm?id=GO:0043425) 2. [chromatin DNA binding](http://www.ebi.ac.uk/QuickGO/GTerm?id=GO:0031490) 3. [core promoter proximal region sequence-specific DNA binding](http://www.ebi.ac.uk/QuickGO/GTerm?id=GO:0000987) 4. [co-SMAD binding](http://www.ebi.ac.uk/QuickGO/GTerm?id=GO:0070410) 5. [enhancer binding](http://www.ebi.ac.uk/QuickGO/GTerm?id=GO:0035326) 6. [identical protein binding](http://www.ebi.ac.uk/QuickGO/GTerm?id=GO:0042802) 7. [phosphatase binding](http://www.ebi.ac.uk/QuickGO/GTerm?id=GO:0019902) 8. [protein homodimerization activity](http://www.ebi.ac.uk/QuickGO/GTerm?id=GO:0042803) 9. [protein kinase binding](http://www.ebi.ac.uk/QuickGO/GTerm?id=GO:0019901) 10. [RNA polymerase II activating transcription factor binding](http://www.ebi.ac.uk/QuickGO/GTerm?id=GO:0001102) 11. [RNA polymerase II core promoter proximal region sequence-specific DNA binding](http://www.ebi.ac.uk/QuickGO/GTerm?id=GO:0000978) 12. [R-SMAD binding](http://www.ebi.ac.uk/QuickGO/GTerm?id=GO:0070412) 13. [sequence-specific DNA binding](http://www.ebi.ac.uk/QuickGO/GTerm?id=GO:0043565) 14. [transcription factor activity, RNA polymerase II core promoter sequence-specific](http://www.ebi.ac.uk/QuickGO/GTerm?id=GO:0000983) 15. [transcription factor activity, sequence-specific DNA binding](http://www.ebi.ac.uk/QuickGO/GTerm?id=GO:0003700) 16. [transcription factor binding](http://www.ebi.ac.uk/QuickGO/GTerm?id=GO:0008134) 17. [transcription regulatory region DNA binding](http://www.ebi.ac.uk/QuickGO/GTerm?id=GO:0044212) 18. [transforming growth factor beta receptor, pathway-specific cytoplasmic mediator activity](http://www.ebi.ac.uk/QuickGO/GTerm?id=GO:0030618) 19. [transforming growth factor beta receptor binding](http://www.ebi.ac.uk/QuickGO/GTerm?id=GO:0005160) 20. [ubiquitin binding](http://www.ebi.ac.uk/QuickGO/GTerm?id=GO:0043130) 21. [ubiquitin protein ligase binding](http://www.ebi.ac.uk/QuickGO/GTerm?id=GO:0031625) 22. [zinc ion binding](http://www.ebi.ac.uk/QuickGO/GTerm?id=GO:0008270)   **GO - Biological process**   1. [activation of cysteine-type endopeptidase activity involved in apoptotic process](http://www.ebi.ac.uk/QuickGO/GTerm?id=GO:0006919) 2. [activation of cysteine-type endopeptidase activity involved in apoptotic signaling pathway](http://www.ebi.ac.uk/QuickGO/GTerm?id=GO:0097296) 3. [activin receptor signaling pathway](http://www.ebi.ac.uk/QuickGO/GTerm?id=GO:0032924) 4. [cell-cell junction organization](http://www.ebi.ac.uk/QuickGO/GTerm?id=GO:0045216) 5. [cell cycle arrest](http://www.ebi.ac.uk/QuickGO/GTerm?id=GO:0007050) 6. [developmental growth](http://www.ebi.ac.uk/QuickGO/GTerm?id=GO:0048589) 7. [embryonic cranial skeleton morphogenesis](http://www.ebi.ac.uk/QuickGO/GTerm?id=GO:0048701) 8. [embryonic foregut morphogenesis](http://www.ebi.ac.uk/QuickGO/GTerm?id=GO:0048617) 9. [embryonic pattern specification](http://www.ebi.ac.uk/QuickGO/GTerm?id=GO:0009880) 10. [endoderm development](http://www.ebi.ac.uk/QuickGO/GTerm?id=GO:0007492) 11. [evasion or tolerance of host defenses by virus](http://www.ebi.ac.uk/QuickGO/GTerm?id=GO:0019049) 12. [extrinsic apoptotic signaling pathway](http://www.ebi.ac.uk/QuickGO/GTerm?id=GO:0097191) 13. [heart looping](http://www.ebi.ac.uk/QuickGO/GTerm?id=GO:0001947) 14. [immune response](http://www.ebi.ac.uk/QuickGO/GTerm?id=GO:0006955) 15. [immune system development](http://www.ebi.ac.uk/QuickGO/GTerm?id=GO:0002520) 16. [in utero embryonic development](http://www.ebi.ac.uk/QuickGO/GTerm?id=GO:0001701) 17. [lens fiber cell differentiation](http://www.ebi.ac.uk/QuickGO/GTerm?id=GO:0070306) 18. [liver development](http://www.ebi.ac.uk/QuickGO/GTerm?id=GO:0001889) 19. [mesoderm formation](http://www.ebi.ac.uk/QuickGO/GTerm?id=GO:0001707) 20. [negative regulation of apoptotic process](http://www.ebi.ac.uk/QuickGO/GTerm?id=GO:0043066) 21. [negative regulation of cell growth](http://www.ebi.ac.uk/QuickGO/GTerm?id=GO:0030308) 22. [negative regulation of fat cell differentiation](http://www.ebi.ac.uk/QuickGO/GTerm?id=GO:0045599) 23. [negative regulation of inflammatory response](http://www.ebi.ac.uk/QuickGO/GTerm?id=GO:0050728) 24. [negative regulation of mitotic cell cycle](http://www.ebi.ac.uk/QuickGO/GTerm?id=GO:0045930) 25. [negative regulation of osteoblast differentiation](http://www.ebi.ac.uk/QuickGO/GTerm?id=GO:0045668) 26. [negative regulation of osteoblast proliferation](http://www.ebi.ac.uk/QuickGO/GTerm?id=GO:0033689) 27. [negative regulation of transcription from RNA polymerase II promoter](http://www.ebi.ac.uk/QuickGO/GTerm?id=GO:0000122) 28. [negative regulation of transforming growth factor beta receptor signaling pathway](http://www.ebi.ac.uk/QuickGO/GTerm?id=GO:0030512) 29. [negative regulation of wound healing](http://www.ebi.ac.uk/QuickGO/GTerm?id=GO:0061045) 30. [nodal signaling pathway](http://www.ebi.ac.uk/QuickGO/GTerm?id=GO:0038092) 31. [osteoblast development](http://www.ebi.ac.uk/QuickGO/GTerm?id=GO:0002076) 32. [paraxial mesoderm morphogenesis](http://www.ebi.ac.uk/QuickGO/GTerm?id=GO:0048340) 33. [pericardium development](http://www.ebi.ac.uk/QuickGO/GTerm?id=GO:0060039) 34. [positive regulation of alkaline phosphatase activity](http://www.ebi.ac.uk/QuickGO/GTerm?id=GO:0010694) 35. [positive regulation of bone mineralization](http://www.ebi.ac.uk/QuickGO/GTerm?id=GO:0030501) 36. [positive regulation of cell migration](http://www.ebi.ac.uk/QuickGO/GTerm?id=GO:0030335) 37. [positive regulation of chondrocyte differentiation](http://www.ebi.ac.uk/QuickGO/GTerm?id=GO:0032332) 38. [positive regulation of epithelial to mesenchymal transition](http://www.ebi.ac.uk/QuickGO/GTerm?id=GO:0010718) 39. [positive regulation of extracellular matrix assembly](http://www.ebi.ac.uk/QuickGO/GTerm?id=GO:1901203) 40. [positive regulation of focal adhesion assembly](http://www.ebi.ac.uk/QuickGO/GTerm?id=GO:0051894) 41. [positive regulation of gene expression](http://www.ebi.ac.uk/QuickGO/GTerm?id=GO:0010628) 42. [positive regulation of interleukin-1 beta production](http://www.ebi.ac.uk/QuickGO/GTerm?id=GO:0032731) 43. [positive regulation of positive chemotaxis](http://www.ebi.ac.uk/QuickGO/GTerm?id=GO:0050927) 44. [positive regulation of stress fiber assembly](http://www.ebi.ac.uk/QuickGO/GTerm?id=GO:0051496) 45. [positive regulation of transcription, DNA-templated](http://www.ebi.ac.uk/QuickGO/GTerm?id=GO:0045893) 46. [positive regulation of transcription factor import into nucleus](http://www.ebi.ac.uk/QuickGO/GTerm?id=GO:0042993) 47. [positive regulation of transcription from RNA polymerase II promoter](http://www.ebi.ac.uk/QuickGO/GTerm?id=GO:0045944) 48. [positive regulation of transforming growth factor beta3 production](http://www.ebi.ac.uk/QuickGO/GTerm?id=GO:0032916) 49. [primary miRNA processing](http://www.ebi.ac.uk/QuickGO/GTerm?id=GO:0031053) 50. [regulation of binding](http://www.ebi.ac.uk/QuickGO/GTerm?id=GO:0051098) 51. [regulation of epithelial cell proliferation](http://www.ebi.ac.uk/QuickGO/GTerm?id=GO:0050678) 52. [regulation of immune response](http://www.ebi.ac.uk/QuickGO/GTerm?id=GO:0050776) 53. [regulation of striated muscle tissue development](http://www.ebi.ac.uk/QuickGO/GTerm?id=GO:0016202) 54. [regulation of transcription from RNA polymerase II promoter](http://www.ebi.ac.uk/QuickGO/GTerm?id=GO:0006357) 55. [regulation of transforming growth factor beta2 production](http://www.ebi.ac.uk/QuickGO/GTerm?id=GO:0032909) 56. [regulation of transforming growth factor beta receptor signaling pathway](http://www.ebi.ac.uk/QuickGO/GTerm?id=GO:0017015) 57. [response to hypoxia](http://www.ebi.ac.uk/QuickGO/GTerm?id=GO:0001666) 58. [signal transduction involved in regulation of gene expression](http://www.ebi.ac.uk/QuickGO/GTerm?id=GO:0023019) 59. [SMAD protein complex assembly](http://www.ebi.ac.uk/QuickGO/GTerm?id=GO:0007183) 60. [SMAD protein signal transduction](http://www.ebi.ac.uk/QuickGO/GTerm?id=GO:0060395) 61. [somitogenesis](http://www.ebi.ac.uk/QuickGO/GTerm?id=GO:0001756) 62. [T cell activation](http://www.ebi.ac.uk/QuickGO/GTerm?id=GO:0042110) 63. [thyroid gland development](http://www.ebi.ac.uk/QuickGO/GTerm?id=GO:0030878) 64. [transcription, DNA-templated](http://www.ebi.ac.uk/QuickGO/GTerm?id=GO:0006351) 65. [transdifferentiation](http://www.ebi.ac.uk/QuickGO/GTerm?id=GO:0060290) 66. [transforming growth factor beta receptor signaling pathway](http://www.ebi.ac.uk/QuickGO/GTerm?id=GO:0007179) 67. [transport](http://www.ebi.ac.uk/QuickGO/GTerm?id=GO:0006810) 68. [ureteric bud development](http://www.ebi.ac.uk/QuickGO/GTerm?id=GO:0001657) 69. [wound healing](http://www.ebi.ac.uk/QuickGO/GTerm?id=GO:0042060) |
| Q99720 | - Amyotrophic lateral sclerosis - Spinal muscular atrophy - Amyotrophic Lateral Sclerosis - Frontotemporal Lobar Degeneration - Amnesia - Prenatal Exposure - Delayed Effects - Seizures - Memory Disorders - Learning Disorders - Cocaine-Related Disorders | | | **GO - Molecular function**   1. [drug binding](http://www.ebi.ac.uk/QuickGO/GTerm?id=GO:0008144)   [opioid receptor activity](http://www.ebi.ac.uk/QuickGO/GTerm?id=GO:0004985)  **GO - Biological process**   1. [lipid transport](http://www.ebi.ac.uk/QuickGO/GTerm?id=GO:0006869) 2. [nervous system development](http://www.ebi.ac.uk/QuickGO/GTerm?id=GO:0007399) 3. [protein homotrimerization](http://www.ebi.ac.uk/QuickGO/GTerm?id=GO:0070207) 4. [regulation of neuron apoptotic process](http://www.ebi.ac.uk/QuickGO/GTerm?id=GO:0043523) |
| Q7Z2H8 | - Malignant neoplasm of breast - Colorectal Neoplasms - Hypertensive disease - Prostatic Neoplasms - Tuberculosis - Iron deficiency - Breast Carcinoma - Chronic lung disease - Perry Syndrome | | | **GO - Molecular function**   1. [amino acid transmembrane transporter activity](http://www.ebi.ac.uk/QuickGO/GTerm?id=GO:0015171) 2. [glycine transmembrane transporter activity](http://www.ebi.ac.uk/QuickGO/GTerm?id=GO:0015187) 3. [hydrogen:amino acid symporter activity](http://www.ebi.ac.uk/QuickGO/GTerm?id=GO:0005280) 4. [hydrogen ion transmembrane transporter activity](http://www.ebi.ac.uk/QuickGO/GTerm?id=GO:0015078) 5. [L-alanine transmembrane transporter activity](http://www.ebi.ac.uk/QuickGO/GTerm?id=GO:0015180) 6. [L-proline transmembrane transporter activity](http://www.ebi.ac.uk/QuickGO/GTerm?id=GO:0015193)   **GO - Biological process**   1. [amino acid transport](http://www.ebi.ac.uk/QuickGO/GTerm?id=GO:0006865) 2. [glycine transport](http://www.ebi.ac.uk/QuickGO/GTerm?id=GO:0015816) 3. [ion transport](http://www.ebi.ac.uk/QuickGO/GTerm?id=GO:0006811) |
| P63092 | - Osteoma cutis - Pseudohypoparathyroidism - Pseudopseudohypoparathyroidism - McCune-Albright Syndrome - Pseudohypoparathyroidism - Polyostotic fibrous dysplasia - Growth Hormone-Secreting Pituitary Adenoma - Pseudohypoparathyroidism Type 1B - Pseudohypoparathyroidism Type 1C - Acth-Independent Macronodular Adrenal Hyperplasia | | | **GO - Molecular function**   1. [GTPase activity](http://www.ebi.ac.uk/QuickGO/GTerm?id=GO:0003924) 2. [GTP binding](http://www.ebi.ac.uk/QuickGO/GTerm?id=GO:0005525) 3. [metal ion binding](http://www.ebi.ac.uk/QuickGO/GTerm?id=GO:0046872) 4. [signal transducer activity](http://www.ebi.ac.uk/QuickGO/GTerm?id=GO:0004871)   **GO - Biological process**   1. [activation of adenylate cyclase activity](http://www.ebi.ac.uk/QuickGO/GTerm?id=GO:0007190) 2. [adenylate cyclase-activating adrenergic receptor signaling pathway](http://www.ebi.ac.uk/QuickGO/GTerm?id=GO:0071880) 3. [adenylate cyclase-activating dopamine receptor signaling pathway](http://www.ebi.ac.uk/QuickGO/GTerm?id=GO:0007191) 4. [adenylate cyclase-activating G-protein coupled receptor signaling pathway](http://www.ebi.ac.uk/QuickGO/GTerm?id=GO:0007189) 5. [bone development](http://www.ebi.ac.uk/QuickGO/GTerm?id=GO:0060348) 6. [cellular response to catecholamine stimulus](http://www.ebi.ac.uk/QuickGO/GTerm?id=GO:0071870) 7. [cellular response to glucagon stimulus](http://www.ebi.ac.uk/QuickGO/GTerm?id=GO:0071377) 8. [cellular response to prostaglandin E stimulus](http://www.ebi.ac.uk/QuickGO/GTerm?id=GO:0071380) 9. [cognition](http://www.ebi.ac.uk/QuickGO/GTerm?id=GO:0050890) 10. [developmental growth](http://www.ebi.ac.uk/QuickGO/GTerm?id=GO:0048589) 11. [hair follicle placode formation](http://www.ebi.ac.uk/QuickGO/GTerm?id=GO:0060789) 12. [intracellular transport](http://www.ebi.ac.uk/QuickGO/GTerm?id=GO:0046907) 13. [platelet aggregation](http://www.ebi.ac.uk/QuickGO/GTerm?id=GO:0070527) 14. [positive regulation of cAMP biosynthetic process](http://www.ebi.ac.uk/QuickGO/GTerm?id=GO:0030819) 15. [positive regulation of cAMP-mediated signaling](http://www.ebi.ac.uk/QuickGO/GTerm?id=GO:0043950) 16. [positive regulation of GTPase activity](http://www.ebi.ac.uk/QuickGO/GTerm?id=GO:0043547) 17. [regulation of insulin secretion](http://www.ebi.ac.uk/QuickGO/GTerm?id=GO:0050796) 18. [renal water homeostasis](http://www.ebi.ac.uk/QuickGO/GTerm?id=GO:0003091) 19. [sensory perception of smell](http://www.ebi.ac.uk/QuickGO/GTerm?id=GO:0007608) |
| P83916 | - Prostatic Neoplasms - Malignant neoplasm of breast - Mammary Neoplasms - Breast Carcinoma - Prostate carcinoma - Thyroid carcinoma - Malignant neoplasm of prostate - Disseminated Malignant Neoplasm - Follicular adenoma - Retinoblastoma | | | **GO - Molecular function**   1. [chromatin binding](http://www.ebi.ac.uk/QuickGO/GTerm?id=GO:0003682) 2. [enzyme binding](http://www.ebi.ac.uk/QuickGO/GTerm?id=GO:0019899) 3. [histone methyltransferase binding](http://www.ebi.ac.uk/QuickGO/GTerm?id=GO:1990226)   **GO - Biological process**   1. [negative regulation of transcription, DNA-templated](http://www.ebi.ac.uk/QuickGO/GTerm?id=GO:0045892) |
| Q9H3R0 | - Medulloblastoma - Esophageal Neoplasms - Mucosa-Associated Lymphoid Tissue - Lymphoma - Anoxia - Squamous cell carcinoma - Autistic Disorder - Tobacco Use Disorder - Pharyngeal Neoplasms - Mouth Neoplasms - Laryngeal neoplasm | | | **GO - Molecular function**   1. [androgen receptor binding](http://www.ebi.ac.uk/QuickGO/GTerm?id=GO:0050681) 2. [dioxygenase activity](http://www.ebi.ac.uk/QuickGO/GTerm?id=GO:0051213) 3. [enzyme binding](http://www.ebi.ac.uk/QuickGO/GTerm?id=GO:0019899) 4. [histone demethylase activity](http://www.ebi.ac.uk/QuickGO/GTerm?id=GO:0032452) 5. [histone demethylase activity (H3-K9 specific)](http://www.ebi.ac.uk/QuickGO/GTerm?id=GO:0032454) 6. [zinc ion binding](http://www.ebi.ac.uk/QuickGO/GTerm?id=GO:0008270)   **GO - Biological process**   1. [histone H3-K9 demethylation](http://www.ebi.ac.uk/QuickGO/GTerm?id=GO:0033169) 2. [positive regulation of cell proliferation](http://www.ebi.ac.uk/QuickGO/GTerm?id=GO:0008284) 3. [regulation of transcription from RNA polymerase II promoter](http://www.ebi.ac.uk/QuickGO/GTerm?id=GO:0006357) 4. [transcription, DNA-templated](http://www.ebi.ac.uk/QuickGO/GTerm?id=GO:0006351) |
| P39748 | - Colorectal Neoplasms - Malignant neoplasm of breast - Malignant neoplasm of lung - Lung Neoplasms - Lupus Erythematosus - Coronary Artery Disease - Huntington Disease - Neoplasms - Hematologic Neoplasms - Neoplasm Recurrence | | | **GO - Molecular function**   1. [5'-3' exonuclease activity](http://www.ebi.ac.uk/QuickGO/GTerm?id=GO:0008409) 2. [5'-flap endonuclease activity](http://www.ebi.ac.uk/QuickGO/GTerm?id=GO:0017108) 3. [damaged DNA binding](http://www.ebi.ac.uk/QuickGO/GTerm?id=GO:0003684) 4. [DNA binding](http://www.ebi.ac.uk/QuickGO/GTerm?id=GO:0003677) 5. [double-stranded DNA binding](http://www.ebi.ac.uk/QuickGO/GTerm?id=GO:0003690) 6. [double-stranded DNA exodeoxyribonuclease activity](http://www.ebi.ac.uk/QuickGO/GTerm?id=GO:0008309) 7. [endonuclease activity](http://www.ebi.ac.uk/QuickGO/GTerm?id=GO:0004519) 8. [exonuclease activity](http://www.ebi.ac.uk/QuickGO/GTerm?id=GO:0004527) 9. [magnesium ion binding](http://www.ebi.ac.uk/QuickGO/GTerm?id=GO:0000287) -HAMAP 10. [RNA-DNA hybrid ribonuclease activity](http://www.ebi.ac.uk/QuickGO/GTerm?id=GO:0004523)   **GO - Biological process**   1. [base-excision repair](http://www.ebi.ac.uk/QuickGO/GTerm?id=GO:0006284) -HAMAP 2. [DNA repair](http://www.ebi.ac.uk/QuickGO/GTerm?id=GO:0006281) 3. [DNA replication](http://www.ebi.ac.uk/QuickGO/GTerm?id=GO:0006260) 4. [DNA replication, removal of RNA primer](http://www.ebi.ac.uk/QuickGO/GTerm?id=GO:0043137) 5. [double-strand break repair](http://www.ebi.ac.uk/QuickGO/GTerm?id=GO:0006302) 6. [double-strand break repair via homologous recombination](http://www.ebi.ac.uk/QuickGO/GTerm?id=GO:0000724) 7. [memory](http://www.ebi.ac.uk/QuickGO/GTerm?id=GO:0007613) 8. [telomere maintenance via recombination](http://www.ebi.ac.uk/QuickGO/GTerm?id=GO:0000722) 9. [UV protection](http://www.ebi.ac.uk/QuickGO/GTerm?id=GO:0009650) |
| P10145 | - Inflammation - Stomach Neoplasms - Mammary Neoplasms - Prostatic Neoplasms - Rheumatoid Arthritis - Squamous cell carcinoma - Adenocarcinoma - Chronic Obstructive Airway Disease - Liver carcinoma - Pancreatic Neoplasm | | | **GO - Molecular function**   1. [chemokine activity](http://www.ebi.ac.uk/QuickGO/GTerm?id=GO:0008009) 2. [interleukin-8 receptor binding](http://www.ebi.ac.uk/QuickGO/GTerm?id=GO:0005153)   **GO - Biological process**   1. [angiogenesis](http://www.ebi.ac.uk/QuickGO/GTerm?id=GO:0001525) 2. [calcium-mediated signaling](http://www.ebi.ac.uk/QuickGO/GTerm?id=GO:0019722) 3. [cell cycle arrest](http://www.ebi.ac.uk/QuickGO/GTerm?id=GO:0007050) 4. [cellular response to fibroblast growth factor stimulus](http://www.ebi.ac.uk/QuickGO/GTerm?id=GO:0044344) 5. [cellular response to interleukin-1](http://www.ebi.ac.uk/QuickGO/GTerm?id=GO:0071347) 6. [cellular response to lipopolysaccharide](http://www.ebi.ac.uk/QuickGO/GTerm?id=GO:0071222) 7. [cellular response to tumor necrosis factor](http://www.ebi.ac.uk/QuickGO/GTerm?id=GO:0071356) 8. [chemokine-mediated signaling pathway](http://www.ebi.ac.uk/QuickGO/GTerm?id=GO:0070098) 9. [chemotaxis](http://www.ebi.ac.uk/QuickGO/GTerm?id=GO:0006935) 10. [embryonic digestive tract development](http://www.ebi.ac.uk/QuickGO/GTerm?id=GO:0048566) 11. [G-protein coupled receptor signaling pathway](http://www.ebi.ac.uk/QuickGO/GTerm?id=GO:0007186) 12. [immune response](http://www.ebi.ac.uk/QuickGO/GTerm?id=GO:0006955) 13. [induction of positive chemotaxis](http://www.ebi.ac.uk/QuickGO/GTerm?id=GO:0050930) 14. [inflammatory response](http://www.ebi.ac.uk/QuickGO/GTerm?id=GO:0006954) 15. [intracellular signal transduction](http://www.ebi.ac.uk/QuickGO/GTerm?id=GO:0035556) 16. [movement of cell or subcellular component](http://www.ebi.ac.uk/QuickGO/GTerm?id=GO:0006928) 17. [negative regulation of cell proliferation](http://www.ebi.ac.uk/QuickGO/GTerm?id=GO:0008285) 18. [negative regulation of G-protein coupled receptor protein signaling pathway](http://www.ebi.ac.uk/QuickGO/GTerm?id=GO:0045744) 19. [neutrophil activation](http://www.ebi.ac.uk/QuickGO/GTerm?id=GO:0042119) 20. [neutrophil chemotaxis](http://www.ebi.ac.uk/QuickGO/GTerm?id=GO:0030593) 21. [PERK-mediated unfolded protein response](http://www.ebi.ac.uk/QuickGO/GTerm?id=GO:0036499) 22. [positive regulation of angiogenesis](http://www.ebi.ac.uk/QuickGO/GTerm?id=GO:0045766) 23. [positive regulation of neutrophil chemotaxis](http://www.ebi.ac.uk/QuickGO/GTerm?id=GO:0090023) 24. [receptor internalization](http://www.ebi.ac.uk/QuickGO/GTerm?id=GO:0031623) 25. [regulation of cell adhesion](http://www.ebi.ac.uk/QuickGO/GTerm?id=GO:0030155) 26. [regulation of single stranded viral RNA replication via double stranded DNA intermediate](http://www.ebi.ac.uk/QuickGO/GTerm?id=GO:0045091) 27. [response to endoplasmic reticulum stress](http://www.ebi.ac.uk/QuickGO/GTerm?id=GO:0034976) 28. [response to molecule of bacterial origin](http://www.ebi.ac.uk/QuickGO/GTerm?id=GO:0002237) 29. [signal transduction](http://www.ebi.ac.uk/QuickGO/GTerm?id=GO:0007165) |
| P08253 | - Torg-Winchester syndrome - Neoplasm Metastasis - Colorectal Neoplasms - Hypertensive disease - Myocardial Infarction - Liver Cirrhosis - Aortic Aneurysm Thoracic - Mammary Neoplasms - Liver carcinoma - Rheumatoid Arthritis | | | **GO - Molecular function**   1. [metalloendopeptidase activity](http://www.ebi.ac.uk/QuickGO/GTerm?id=GO:0004222) 2. [metallopeptidase activity](http://www.ebi.ac.uk/QuickGO/GTerm?id=GO:0008237) 3. [serine-type endopeptidase activity](http://www.ebi.ac.uk/QuickGO/GTerm?id=GO:0004252) 4. [zinc ion binding](http://www.ebi.ac.uk/QuickGO/GTerm?id=GO:0008270)   **GO - Biological process**   1. [angiogenesis](http://www.ebi.ac.uk/QuickGO/GTerm?id=GO:0001525) 2. [blood vessel maturation](http://www.ebi.ac.uk/QuickGO/GTerm?id=GO:0001955) 3. [bone trabecula formation](http://www.ebi.ac.uk/QuickGO/GTerm?id=GO:0060346) 4. [cellular protein metabolic process](http://www.ebi.ac.uk/QuickGO/GTerm?id=GO:0044267) 5. [cellular response to amino acid stimulus](http://www.ebi.ac.uk/QuickGO/GTerm?id=GO:0071230) 6. [collagen catabolic process](http://www.ebi.ac.uk/QuickGO/GTerm?id=GO:0030574) 7. [embryo implantation](http://www.ebi.ac.uk/QuickGO/GTerm?id=GO:0007566) 8. [endodermal cell differentiation](http://www.ebi.ac.uk/QuickGO/GTerm?id=GO:0035987) 9. [ephrin receptor signaling pathway](http://www.ebi.ac.uk/QuickGO/GTerm?id=GO:0048013) 10. [extracellular matrix disassembly](http://www.ebi.ac.uk/QuickGO/GTerm?id=GO:0022617) 11. [face morphogenesis](http://www.ebi.ac.uk/QuickGO/GTerm?id=GO:0060325) 12. [intramembranous ossification](http://www.ebi.ac.uk/QuickGO/GTerm?id=GO:0001957) 13. [positive regulation of innate immune response](http://www.ebi.ac.uk/QuickGO/GTerm?id=GO:0045089) 14. [proteolysis](http://www.ebi.ac.uk/QuickGO/GTerm?id=GO:0006508) 15. [response to hypoxia](http://www.ebi.ac.uk/QuickGO/GTerm?id=GO:0001666) |
| Q9NUW8 | - Spinocerebellar ataxia - Ataxia - Colorectal Neoplasms - Axonal neuropathy - Neurodegenerative Disorders - Carcinoma of lung - Neutropenia - Malignant neoplasm of lung - Leukopenia - Mammary Neoplasms | | | **GO - Molecular function**   1. [3'-tyrosyl-DNA phosphodiesterase activity](http://www.ebi.ac.uk/QuickGO/GTerm?id=GO:0017005) 2. [double-stranded DNA binding](http://www.ebi.ac.uk/QuickGO/GTerm?id=GO:0003690) 3. [exonuclease activity](http://www.ebi.ac.uk/QuickGO/GTerm?id=GO:0004527) 4. [single-stranded DNA binding](http://www.ebi.ac.uk/QuickGO/GTerm?id=GO:0003697)   **GO - Biological process**   1. [DNA repair](http://www.ebi.ac.uk/QuickGO/GTerm?id=GO:0006281) 2. [double-strand break repair](http://www.ebi.ac.uk/QuickGO/GTerm?id=GO:0006302) 3. [single strand break repair](http://www.ebi.ac.uk/QuickGO/GTerm?id=GO:0000012) |

Listed are the 10 most common diseases for each Uniprot entry base using DisGeNet database.

The GO molecular function and biological processes were extracted from Uniprotkb.

**Table S6. PANTHER overrepresentation test of metformin virtual hits**

| **GO Molecular Function*** | **REF^a^** | **#** | **Expected** | **Fold-enrichment** |  | **P value^b^** |
| --- | --- | --- | --- | --- | --- | --- |
| **Transition metal ion binding** | 1387 | 12 | 2.65 | 5.54 |  | 1.76E-02 |
| **Zinc ion binding** | 1125 | 11 | 2.15 | 5.13 |  | 1.48E-02 |
| **Serine hydrolase activity** | 281 | 6 | 0.54 | 11.20 |  | 3.89E-02 |
| **Serine-type peptidase activity** | 278 | 6 | 0.53 | 11.32 |  | 3.66E-02 |
| **Metallopeptidase activity** | 192 | 6 | 0.37 | 16.38 |  | 4.48E-03 |
| **Organic anion transmembrane transporter activity** | 194 | 6 | 0.37 | 16.22 |  | 4.76E-03 |
| **Bile acid transmembrane transporter activity** | 14 | 3 | 0.03 | >100 |  | 7.48E-03 |
| **Secondary active transmembrane transporter activity** | 236 | 6 | 0.45 | 13.33 |  | 1.45E-02 |
| **Secondary active organic cation transmembrane transporter activity** | 3 | 2 | 0.01 | >100 |  | 4.12E-02 |
| **Acetate ester transmembrane transporter activity** | 3 | 2 | 0.01 | >100 |  | 4.12E-02 |
| **Acetylcholine transmembrane transporter activity** | 3 | 2 | 0.01 | >100 |  | 4.12E-02 |
| **Organic hydroxyl compound transmembrane transporter activity** | 38 | 5 | 0.07 | 68.99 |  | 3.16E-05 |
| **Norepinephrine transmembrane transporter activity** | 3 | 2 | 0.01 | >100 |  | 4.12E-02 |
| **Arginase activity** | 2 | 2 | 0.00 | >100 |  | 1.83E-02 |
|  |  |  |  |  |  |  |

* Annotation Data Set: GO molecular function complete

^a^ *Homo sapiens*

^b^ Displaying only results with P<0.05

**Table S7. Key interacting residues of metformin binding to DNA/histone-related virtual targets**

| **Target** | **PDBID** | **Crystal. ligand** | **Key residues^a^** | **Key residues^b^** | **Key residues^c^** |
| --- | --- | --- | --- | --- | --- |
| **O75164 (KDM4A)** | 5FY8 | N81 | K241/216  S196/171  S288/263  L198/173 | N290/265  D191/166  E190/165  K241/216 | N290/265  D191/166  E190/165  K241/216  S196/171 |
| **Q9H3R0 (KDM4C)** | 5KR7 | 6X9 | K208  F187  Y179  N282  Y134 | F187  K243  H190  E192  S198  Y179  N292 | H190  Y179  H278  N292  E192  K243 |
| **Q92830 (GCN5/KAT2A)** | 5H86 | BCO | C85/579  V93/587  G95/589  Y123/Y617  G97/591  Q92/586  L37/531  G126/G620  Y127/Y621  T98/592 | I82  T118  Y119 | T118  Y119  V83  F128  A124 |
| **Q92830 (GCN5/KAT2A)** | 3D7C | None | V757  A762  Y765  C804  N808  Y814 | Y765  M808  I773  P752 | Y765  I773  P752  V757  Y814  F753 |
| **O75495 (GMNN)** | 2WVR | None | E153  Y150  R157  L154  H147  I155  N159  E156 | E153  R157 | E153  R157  Y150 |
| **Q9UNA4 (POLI)** | 1T3N | TTP | K634/239  Y488/93  R491/96  C457/62  T485/90  D546/151  F458/63 | C457/62  T485/90  Y488/93  R491/96  K634/239  D454/59 | Y488/93  R491/96  K634/239  D454/59  D546/151 |
| **O95050 (INMT)** | 2A14 | SAH | Y69  D85  T87  V143  D142  Y25  Y20  L163  G63  F86  A169 | Y20  Y25  Y24  Y204  L164  T198 | F15  G65  L164  G63  L163  A165  T67  Y25  Y20 |
| **P83916 (CBX1)** | 2FMM | None | Y173  S172  H171  W170  T169  L168  R167  E166  R121  E120  K137  D142 | E166  I122  V159  R121 | R121  I122  S162 |
| **P83916 (CBX1)** | 3F2U | None | L40  W42  F45  D49  E53  N57  L58 | D31/49  W24/42 | W24/42  T33/51  D31/49 |
| **P39748 (FEN1)** | 1UL1 | None | Y234  F156  D666  D34  E160  D179  D181  D233 | Y234  A279 | Y234  A279  I228  L282  F283  F185  V224 |
| **Q9NUW8 (TDP1)** | 1QZQ | None | N283  H493  K495  N516 | H493  N283  D288  G458 | H493  N283  N516  K495  S399  S459  Y204 |

^a^ Reference key interacting residues defined using Poseview (Stierand et al., 2010), within the RSC PDB web page, and/or from the literature or databases such as Uniprotkb or Interpro (Finn et al., 2017).

^b^ Key interacting residues using PLIP to show metformin binding

^c^ Key interacting residues using LigPlot^+^ to show metformin binding

The differences in the residues shown by PLIP and LigPlot^+^ are because the interaction distances defined in the two profiling software packages are slightly different; therefore, different interaction and interacting residues are shown. Moreover, in LigPlot^+^, only hydrogen bonds and hydrophobic interactions are shown. The residue number corresponds to the original PDB file numbering. For the PDB ID whose residue number does not correspond to the “actual numbering”, both are indicated separated by a diagonal bar (Aa one letter code + PDB numbering / actual numbering).

**Table S8. Predicted activities of metformin against DNA/histone-related virtual targets**

| **Target** | **Function** | **Similar molecule(s)*** |  | **Phenotype** |  |  |
| --- | --- | --- | --- | --- | --- | --- |
| **O75164 (KDM4A)** | *Epigenetic regulator* | CHEMBL1730756  CHEMBL1735934  CHEMBL1720374 |  | Inhibitor |  |  |
| **Q9H3R0 (KDM4C)** | *Epigenetic regulator* | CHEMBL2071036 |  | Inhibitor |  |  |
| **Q92830 (GCN5/KAT2A)** | *Epigenetic regulator* | CHEMBL1431 |  | Inhibitor |  |  |
| **O75495 (GMNN)** | *Epigenetic regulator* | CHEMBL65567  CHEMBL1431 |  | Activator |  |  |
| **Q9UNA4 (POLI)** | *Enzyme* | CHEMBL6567 |  | Inhibitor |  |  |
| **O95050 (INMT)** | *Enzyme* | CHEMBL167401 |  | Inhibitor |  |  |
| **P83916 (CBX1)** | *Epigenetic regulator* | CHEMBL1720374 |  | Inhibitor |  |  |
| **P39748 (FEN1)** | *Enzyme* | CHEMBL65567 |  | Inhibitor |  |  |
| **Q9NUW8 (TDP1)** | *Enzyme* | CHEMBL1444798 |  | Inhibitor |  |  |

The table lists molecules similar to metformin from which possible therapeutic targets were extrapolated in the VP. The predicted phenotype of metformin is listed accordingly. Both the function of the targets and the identifiers of the molecules extracted from CHEMBL are shown.

* CHEMBL structures of similar molecules can be found in **Table S8**

**Table S9. DNA/histone-related virtual targets of metformin: CHEMBL structures of similar molecules from which the targets were extrapolated in the ligand-based VP**


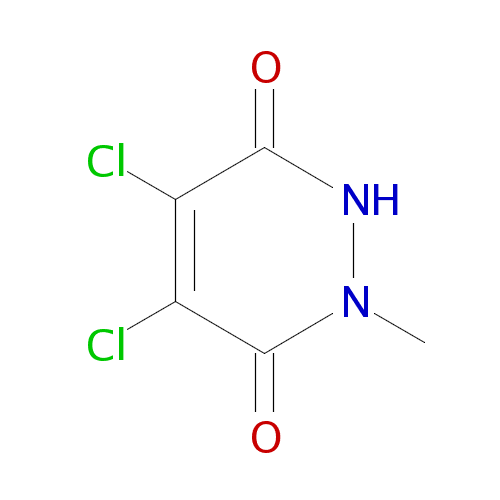


CHEMBL1730756


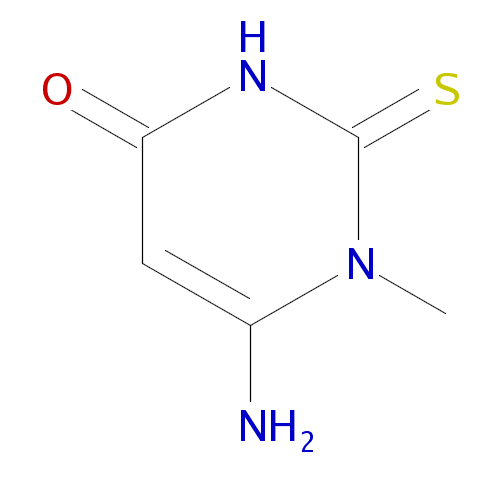


CHEMBL1735934


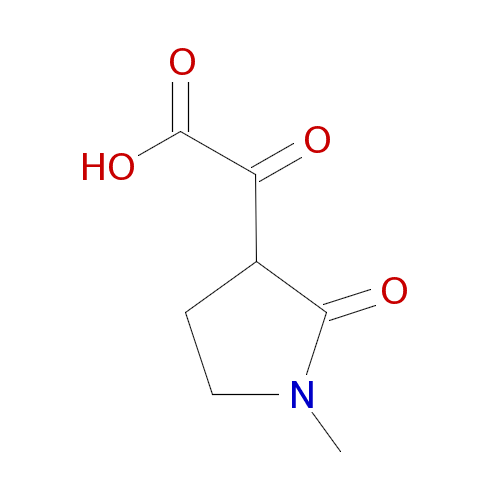


CHEMBL1720374


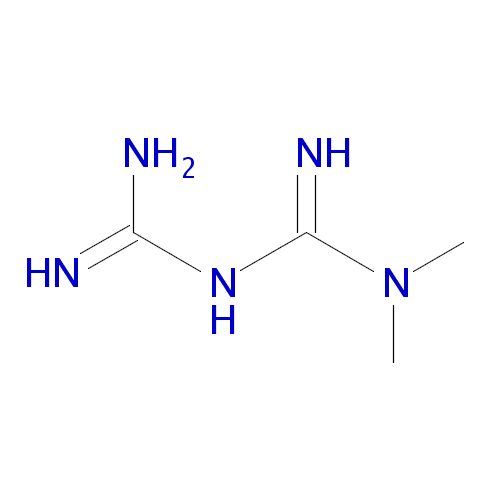


CHEMBL1431


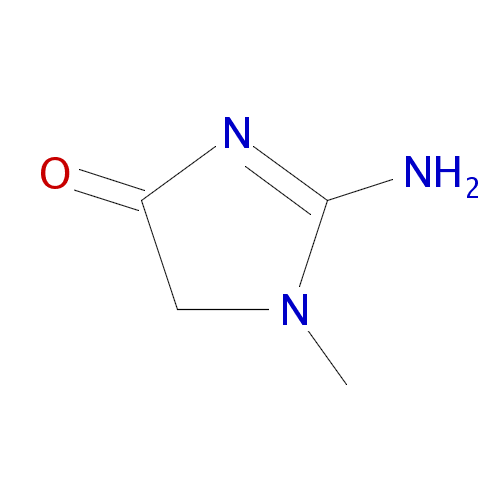


CHEMBL65567


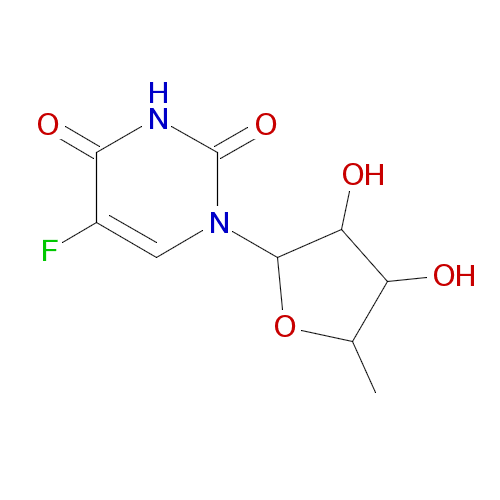


CHEMBL6567


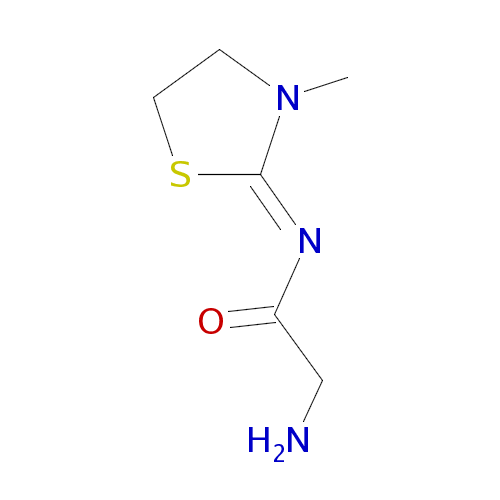


CHEMBL167401


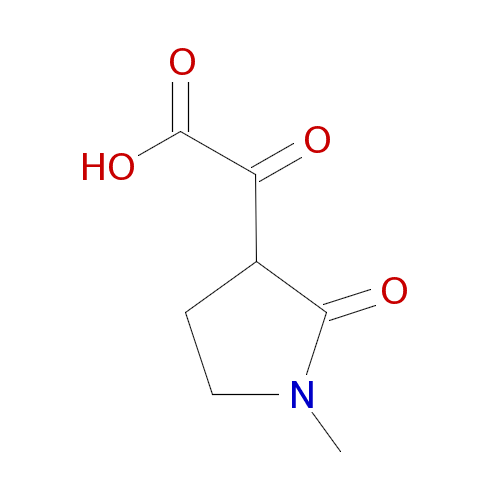


CHEMBL1720374


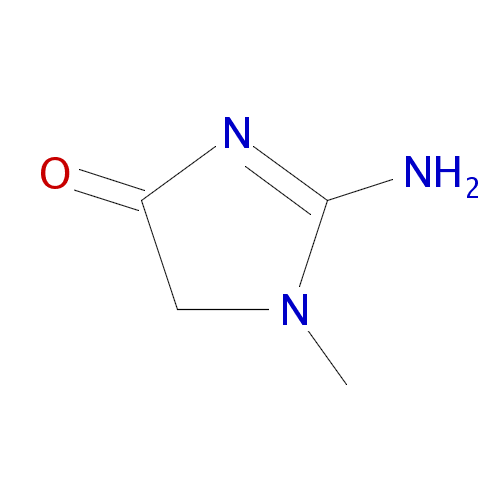


CHEMBL65567

**
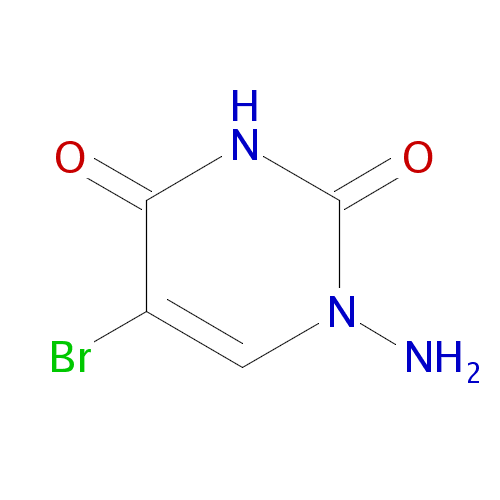
**

CHEMBL1444798
